# Novel Quinoline-Based Thiosemicarbazide Derivatives: Synthesis, DFT Calculations, and Investigation of Antitubercular, Antibacterial, and Antifungal Activities

**DOI:** 10.1021/acsomega.3c03018

**Published:** 2023-10-17

**Authors:** Esma Özcan, Siva Krishna Vagolu, Miyase Gözde Gündüz, Milena Stevanovic, Zülbiye Kökbudak, Tone Tønjum, Jasmina Nikodinovic-Runic, Yasin Çetinkaya, Şengül Dilem Doğan

**Affiliations:** † Department of Chemistry, Faculty of Science, Erciyes University, 38039 Kayseri, Turkey; ‡ Department of Basic Sciences, Faculty of Pharmacy, Erciyes University, 38039 Kayseri, Turkey; § Unit for Genome Dynamics, Department of Microbiology, University of Oslo, 0316 Oslo, Norway; ∥ Department of Pharmaceutical Chemistry, Faculty of Pharmacy, Hacettepe University, Sıhhiye, 06100 Ankara, Turkey; ⊥ Institute of Molecular Genetics and Genetic Engineering, University of Belgrade, 11000 Belgrade, Serbia; # Unit for Genome Dynamics, Department of Microbiology, Oslo University Hospital, 0316 Oslo, Norway; ∇ Department of Chemistry, Faculty of Science, Atatürk University, 25240 Erzurum, Turkey

## Abstract

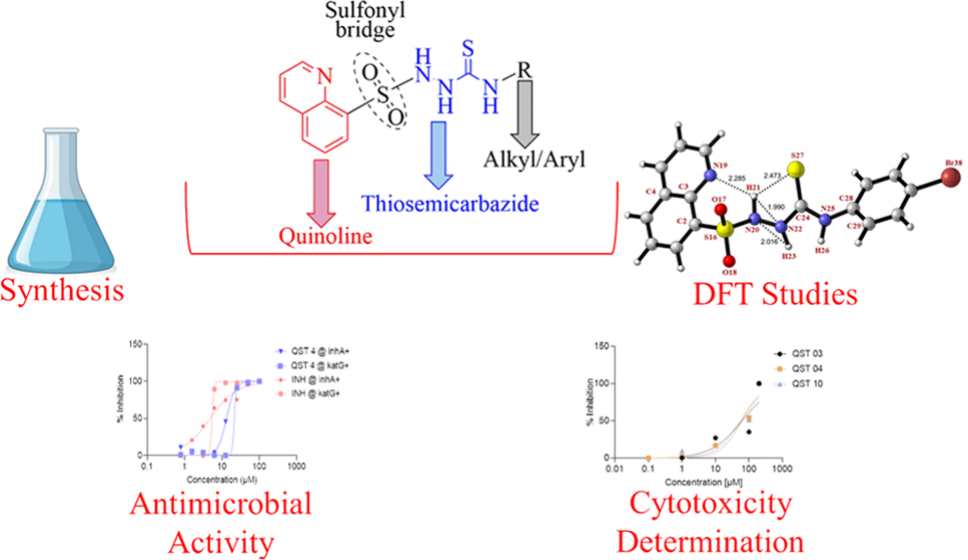

The discovery of new antimicrobial agents as a means of treating drug-resistant microbial pathogens is of utmost significance to overcome their immense risk to human well-being. The current investigation involves the development, synthesis, and assessment of the antimicrobial efficacy of novel quinoline derivatives incorporating a thiosemicarbazide functionality. To design the target compounds (**QST1**–**QST14**), we applied the molecular hybridization approach to link various thiosemicarbazides to the quinoline core with a sulfonyl group. Upon the synthesis and completion of structural characterization via spectroscopic techniques (^1^H NMR, ^13^C NMR, ^15^N NMR, IR, and HRMS), the title molecules were extensively evaluated for their potential antitubercular, antibacterial, and antifungal activities. *N-*(3-Chlorophenyl)-2-(quinolin-8-ylsulfonyl)hydrazine-1-carbothioamide (**QST4**), the most effective compound against *Mycobacterium tuberculosis* H37Rv, was also tested on isoniazid-resistant clinical isolates with *katG* and *inhA* promoter mutations. Based on molecular docking studies, **QST4** was also likely to demonstrate its antimycobacterial activity through inhibition of the InhA enzyme. Furthermore, three derivatives (**QST3**, **QST4**, and **QST10)** with preferable antimicrobial and drug-like profiles were also shown to be nontoxic against human embryonic kidney (HEK) cells. All compounds were optimized by the density functional theory method using B3LYP with the 6-31+G(d,p) basis set. Structural analysis, natural bond orbital calculations of donor–acceptor interactions, molecular electrostatic potential analysis, and frontier molecular orbital analysis were carried out. Quantum chemical descriptors and charges on the atoms were determined to compare the strengths of the intramolecular hydrogen bonds formed and their stabilities. We determined that the sulfur atom forms a stronger intramolecular hydrogen bond than the nitrogen, oxygen, and fluorine atoms in these sulfonyl thiosemicarbazide derivatives.

## 1 Introduction

Despite their century-long existence, infectious diseases caused by bacterial, fungal, and mycobacterial pathogens continue to represent a grave threat to human health.^1^ Bacterial infections, along with cardiovascular diseases and cancer, are one of the main causes of significant morbidity and mortality worldwide.^2^ Tuberculosis (TB), a contagious disease caused by bacteria in the *Mycobacterium tuberculosis* (*Mtb*) complex, has wreaked havoc on humanity for ages.^3^ According to the recent Global TB Report of the World Health Organization (WHO), TB was the leading cause of death from a single infectious agent in 2022.^4^ The WHO goal of gaining successful clinical control of TB by 2030 is frequently put in jeopardy by the emergence of *Mtb* strains resistant to the current chemotherapy regimen, which consists mainly of the four first-line drugs isoniazid, rifampicin, pyrazinamide, and ethambutol.^5^ At the same time, the prevalence of fungal infections is increasing drastically, posing a formidable challenge for the healthcare system.^6^ This alarming increase is directly attributable to the expanding population of immunocompromised patients due to infections such as AIDS, long-term intensive care, organ transplantation, and immunosuppressive drugs.^7^

In spite of ongoing attempts to identify and commercialize new antimicrobial drugs, the prevalence or spread of infectious diseases has never decreased. The rise of multidrug-resistant (MDR) microbial strains is the fundamental hurdle to achieving comprehensive control of infectious diseases.^8^ This situation emphasizes the crucial need for the development of novel antimicrobial agents with distinct chemical structures and modes of action.

**Figure 1:**
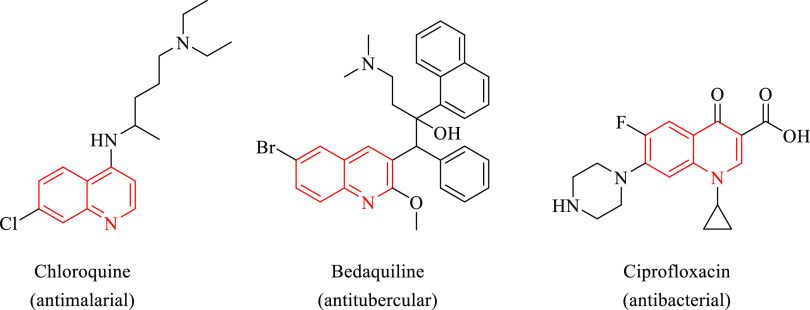
Chemical structures of some quinoline-based antimicrobial drug molecules.

Quinoline or benzo[*b*]pyridine is a nitrogen-containing heteroaromatic ring that attracts significant attention as a core moiety in drug design and development processes endowed with a wide spectrum of biological activities.^9^ Particularly, quinoline-based compounds have been demonstrated to be efficient inhibitors of microbial pathogens (Figure 1).^10^ Among them, chloroquine was developed as a synthetic antimalarial compound, interfering with the digestion of hemoglobin in the blood stages of the malaria life cycle, whose clinical use is still recommended by WHO.^11^ Additionally, bedaquiline is a diarylquinoline derivative that inhibits *Mtb* ATP generation by interfering with the F-ATP synthase activity, which is approved for the treatment of MDR-TB.^12^ Moreover, ciprofloxacin is a representative member of a large group of antibacterial agents known as fluoroquinolones, inhibiting DNA topoisomerase and DNA supercoiling.^13^

**Figure 2:**
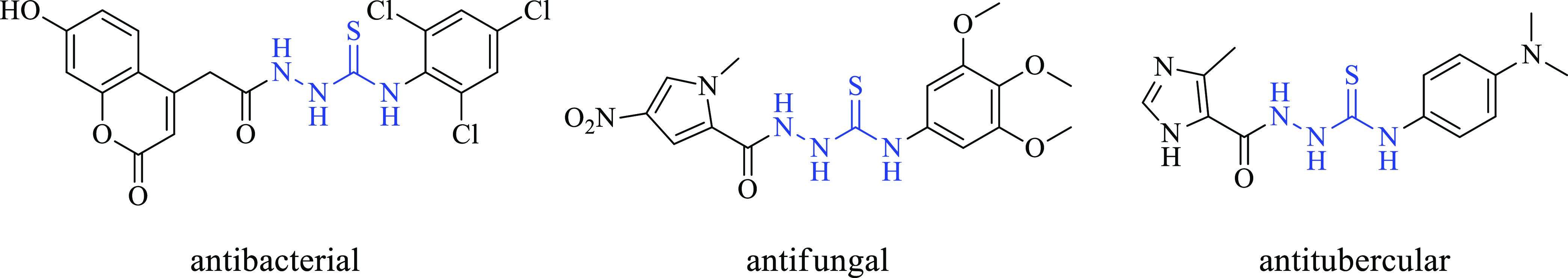
Representative antimicrobial molecules including thiosemicarbazide functionality.

Thiosemicarbazide (NH_2_–NH–(C=S)–NH_2_) functionality offers great chemical versatility and varying biological profiles compared to its oxygen-counterpart semicarbazide due to the presence of the sulfur atom. Besides its vast array of biological activities such as anticonvulsant, anticancer, and antioxidant,^14^ thiosemicarbazide is widely employed as a significant backbone in the antimicrobial drug design and development processes.^15−17^ (Figure 2)

As is commonly known, hydrogen bonds (H-bonds) comprise a combination of a N–H or an O–H proton donor and an O or a N proton acceptor, and depending on the H-bond distance and geometry, the corresponding stabilization energies can be in the range of several to 10 kcal/mol.^18^ However, there are a few but interesting studies on H-bonds containing a S–H proton donor or a S proton acceptor. Recent findings supported by quantum chemical calculations and including spectroscopic evidence have revealed that sulfur-containing H-bonds can be as strong as conventional H-bonds.^19−24^ In another study, to compare amide N–H··S H-bonds with classical σ- and π-type H-bonds, the strength of sulfur-containing H-bonds formed between backbone amides in proteins and methionine or cysteine was found to be as strong as conventional σ-type H-bonds.^25^ What is surprising here is that although the electronegativity of the sulfur atom according to the Pauling scale is lower than that of the fluorine, oxygen, and nitrogen atoms,^26,27^ it can form stronger hydrogen bonds.

**Figure 3:**
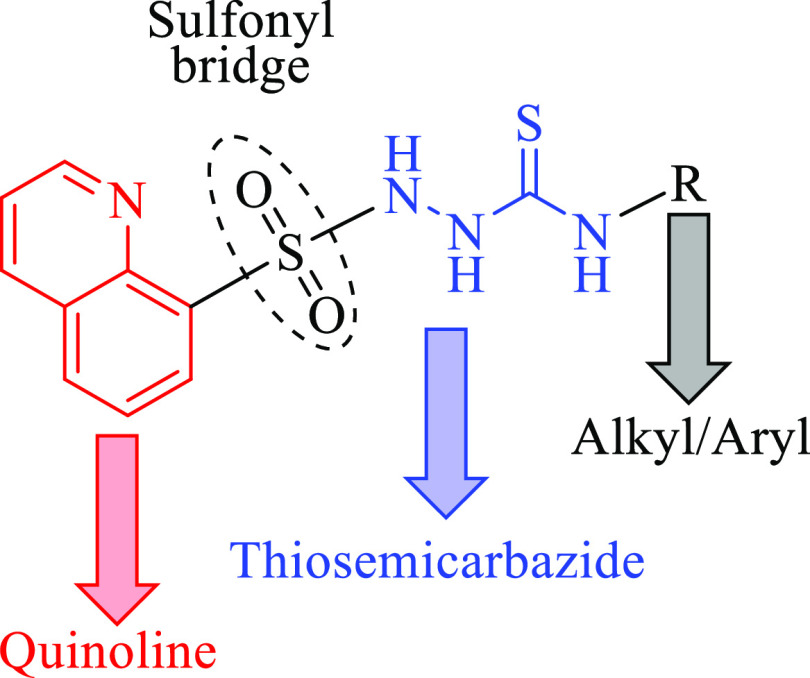
Design strategy and the general chemical structure of the target molecules.

In line with these considerations, we aimed to link quinoline and thiosemicarbazide pharmacophores with a sulfonyl bridge in the same molecule to yield new antimicrobial drug candidates (Figure 3). In this way, we followed the principles of the molecular hybridization approach, a rational method for drug design that entails the merging of at least two pharmacophores derived from diverse bioactive substances into one chemical entity. The primary goal of this approach is to enhance the biological activity profile of the resulting molecule as compared to the parent compounds.^28^

In addition, to examine the structural properties, chemical stability, and reactivity of target molecules, all compounds were optimized by density functional theory (DFT) and also investigated to understand the stability of the most active derivatives. To examine the existence and nature of intramolecular sulfur hydrogen bond interactions in all compounds, we performed DFT calculations, including structural analysis, natural bond orbital analysis (NBO), frontier molecular orbital analysis (FMO), stabilities of compounds with structure isomers, and molecular electrostatic potential (MEP).

## 2 Results and Discussion

### 2.1 Chemistry

**Scheme 1:**
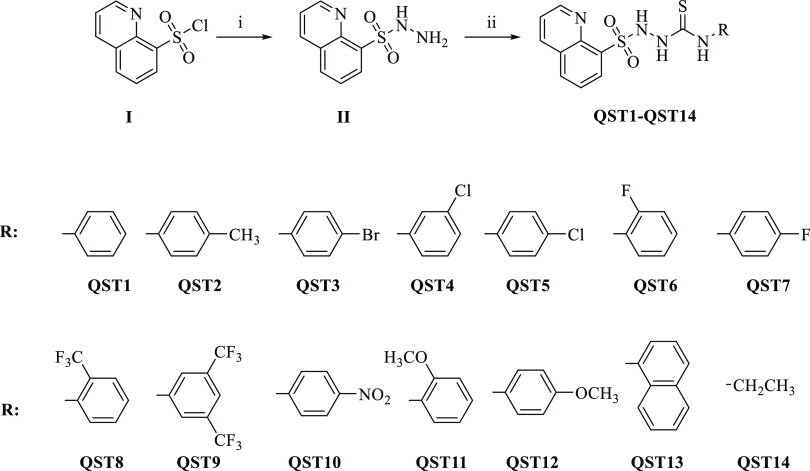
Synthesis and Chemical Structures of **QST1**–**QST14** Reagents and conditions: (**i**) NH_2_NH_2_.H_2_O/reflux and (**ii**) various phenyl/naphthyl/alkyl isothiocyanates (RNCS), dry toluene, and reflux

The general route for the synthesis of the quinoline-thiosemicarbazide hybrid derivatives (**QST1**–**QST14**) is depicted in Scheme 1. Initially, quinoline-8-sulfonohydrazide (**II**) was obtained via the reaction of quinoline-8-sulfonyl chloride (**I**) with hydrazine hydrate. Then, the target molecules were synthesized by the reaction of quinoline-8-sulfonohydrazide (**II**) with various isothiocyanates.

The chemical structures of all compounds were analyzed by ^1^H NMR, ^13^C NMR, IR, and HRMS. In addition to these analyses, a 1D ^15^N NMR analysis was performed only for **QST3**. The IR spectra of **QST1**–**QST14** showed two bands at 1353–1319 and 1170–1143 cm^–1^ corresponding to the SO_2_ group. Besides, characteristic C=S bands as well as the presence of the two NH bands were observed at 1220–1209, 3340–3291, and 3143–2953 cm^–1^, respectively. The ^1^H NMR spectra of **QST1**–**QST14** revealed the presence of three D_2_O-exchangeable signals attributable to the NH groups of the thiosemicarbazide in the region δ 10.52–9.46 ppm. These signals appeared as singlets, with the exception of **QST14**, for which the N^4^–H proton of the thiosemicarbazide moiety appeared at δ 8.21 ppm as a triplet (*J* = 5.6 Hz) because of the electron donor alkyl group. The formation of thiosemicarbazides was also confirmed by ^13^C NMR studies. The C=S signal in thiosemicarbazides **QST1–QST14** was detected between 180.3 and 180.0. The remaining carbon signals were in accordance with the expected ^13^C NMR spectra. The chemical shifts δ (^15^N) have been measured for **QST3** only. The different types of nitrogen N_quinoline_ and three N_thiosemicarbazides_ were characterized using ^15^N NMR measurement of **QST3**. Nitrogen signals of **QST3** were observed at 300.4, 300.2, 128.8, and 126.5 ppm, as expected. The detailed reaction procedures and spectral data of the target compounds are provided in the Experimental and Supporting Information sections, respectively.

### 2.2 Evaluation of Antimicrobial Activity and Cytotoxicity

#### 2.2.1 Antitubercular Activity

We initially tested all of the newly synthesized molecules (**QST1**–**QST14**) for their antitubercular activity against *Mtb* H37Rv by employing the Microplate Alamar Blue Assay (MABA) method and reported the screening results as minimum inhibitory concentration (MIC) values in Table 1.

**Table 1:**
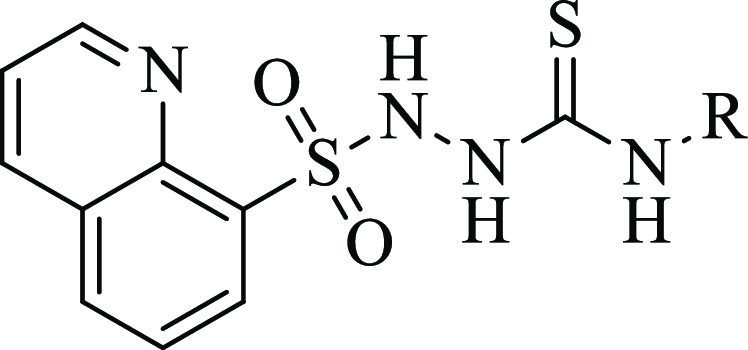
MIC Values of the Synthesized Compounds against *Mtb* H37Rv

compound	R	MIC (μM)
**QST1**	phenyl	50
**QST2**	4-methylphenyl	25
**QST3**	4-bromophenyl	**12.5**
**QST4**	3-chlorophenyl	**6.25**
**QST5**	4-chlorophenyl	25
**QST6**	2-fluorophenyl	25
**QST7**	4-fluorophenyl	50
**QST8**	2-(trifluoromethyl)phenyl	200
**QST9**	3,5-bis(trifluoromethyl)phenyl	200
**QST10**	4-nitrophenyl	**12.5**
**QST11**	2-methoxyphenyl	50
**QST12**	4-methoxyphenyl	25
**QST13**	1-naphthyl	100
**QST14**	ethyl	200
**Isoniazid (INH)**		0.19

Based on the obtained data, the compounds inhibited the growth of *Mtb* H37Rv with MIC values of 6.25–200 μM. The most active derivative in this series appeared to be **QST4**, with an MIC value of 6.25 μM. Additionally, **QST3** and **QST10** were noteworthy compounds with an anti-*Mtb* MIC value of 12.5 μM.

When the correlation between the chemical structure of the compounds and their antitubercular activity was analyzed, it was obvious that the type of substituent on the thiosemicarbazide group directly determined the biological activity profile. In general, electron-withdrawing substituents on the phenyl ring were most influential on antimycobacterial activity. The most active three derivatives (**QST3**, **QST4**, and **QST10**) carried bromine, chlorine, and nitro groups on the phenyl moiety. However, introducing trifluoromethyl onto the phenyl ring resulted in a decrease in antitubercular activity. Additionally, swapping the (substituted)-phenyl ring with a bulky naphthyl moiety (**QST13**) or a small alkyl group (**QST14**) resulted in high MIC values compared to the phenyl-carrying derivatives.

**Figure 4:**
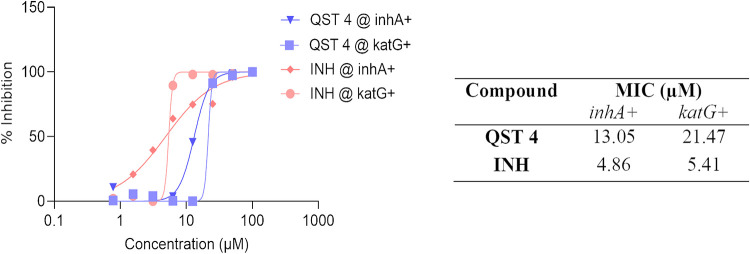
MIC_50_ values of **QST4** and **INH** against INH-resistant *Mtb* clinical isolates carrying the *inhA* promoter or *katG* mutations.

Isoniazid (INH) marked a significant milestone in clinical research, as it was the first drug synthesized to exhibit bactericidal activity against *Mtb*, hence revolutionizing TB chemotherapy. Years after its introduction as a TB drug, INH continues to be an essential element of modern TB treatment. However, the efficacy of this cornerstone drug has notably declined owing to the emergence of INH-resistant strains of *Mtb*, and this resistance is primarily associated with the gene encoding the activator (*KatG*) and the promoter of the target gene (*InhA*) of INH.^29^ Therefore, we additionally assessed the antitubercular activity of **QST4** and INH against INH-resistant *Mtb* clinical isolates with mutations in *katG* and the *inhA* promoter (Figure 4). Based on the MIC values obtained, **QST4** also has inhibitory activity against the drug-resistant clinical isolates of *Mtb*.

#### 2.2.2 Antibacterial and Antifungal Activity

The synthesized compounds were also evaluated for their antibacterial activity against strains of two Gram-positive (*Staphylococcus aureus* and *Enterococcus faecium*) and two Gram-negative (*Pseudomonas aeruginosa* and *Klebsiella pneumonie*) bacterial species as well as for their antifungal activity against an isolate of the most common *Candida* species, *Candida albicans*. The minimum inhibitory concentration (MIC) values of the molecules tested are provided in Supporting Information Table S7. Based on the obtained MIC values, most of the compounds exhibited only limited antimicrobial activity against bacterial and *Candida* species. The most attractive antifungal molecule in this series was found to be **QST10**, which was effective against *C. albicans* at an MIC value of 31.25 μg/mL. **QST8** and **QST9** inhibited the growth of *S. aureus* with the MIC value of 250 μg/mL. Additionally, **QST2** was found to exert moderate antifungal activity toward *C. albicans* with an MIC value of 250 μg/mL.

#### 2.2.3 Cytotoxicity Determination

**Figure 5:**
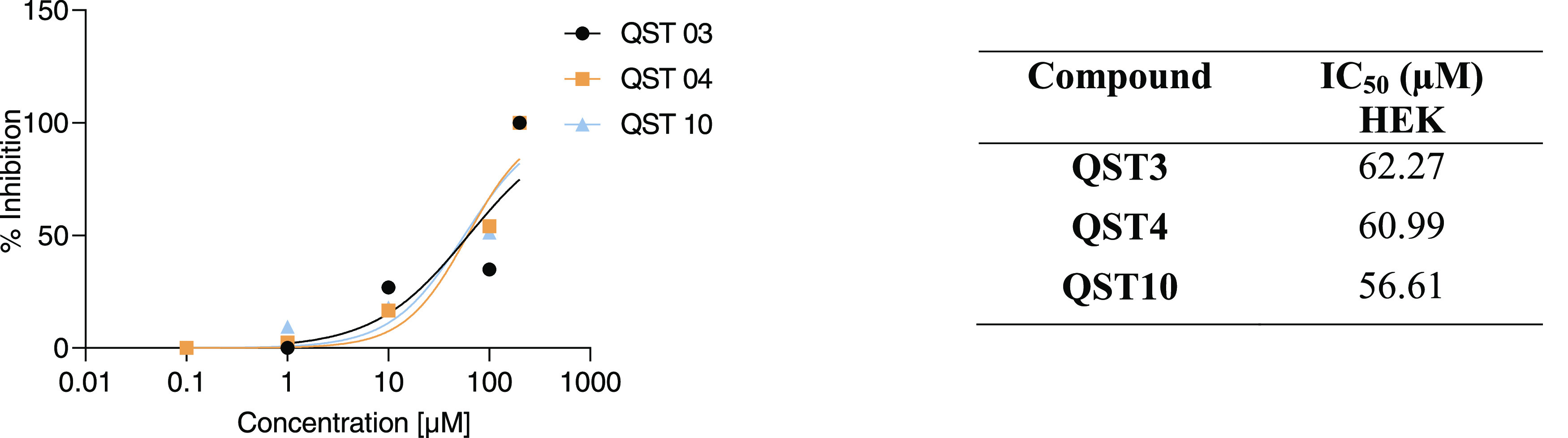
IC_50_ values of the selected compounds against host HEK cell lines.

The development of novel antimicrobial agents poses a significant challenge due to the potential harm they may inflict upon healthy eukaryotic host cells. Therefore, we conducted a colorimetric MTT assay on three selected compounds (**QST3**, **QST4**, and **QST10**) from initial antimicrobial testing to evaluate their cytotoxicity against human embryonic kidney (HEK) cells. Our findings indicate that these bioactive compounds exhibited low toxicity on HEK cells with considerably high IC_50_ values (Figure 5).

### 2.3 Molecular Modeling Studies

#### 2.3.1 Prediction of Drug-Likeness

Certain bioactive compounds may not be effective as drug molecules due to their unfavorable physicochemical characteristics. Thus, it is beneficial to forecast the molecular properties of potential drug candidates for enhancing drug development processes. In the present study, we utilized computational calculations to anticipate key parameters for the most potent antimicrobial agents in this series (**QST3**, **QST4**, and **QST10**) and evaluated their suitability as pharmaceuticals based on “drug-likeness” criteria. The application of Lipinski’s rule of five^30^ serves to estimate the drug-likeness and elucidates the fundamental molecular descriptors that play a crucial role in forecasting the oral bioavailability of novel bioactive compounds. Furthermore, we computed two more descriptors; NRB (number of rotatable bonds) and TPSA (topological polar surface area), which are acknowledged as significant parameters in drug discovery pathways.^31^ (Table 2)

**Table 2:** Calculated Molecular Characteristics of Selected Compounds

compound	M.W.^a^	Log *P*^b^	HBA^c^	HBD^d^	Lipinski’s violation	TPSA^e^	NRB^f^
**QST3**	437.34	2.78	6	3	0	83.11	6
**QST4**	392.89	2.63	6	3	0	83.11	6
**QST10**	403.44	1.93	9	3	0	128.94	7

a MW: molecular weight.

b Log *P*: logarithm of the n-octanol–water partition coefficient.

c HBA: number of hydrogen bond acceptors.

d HBD: number of hydrogen bond donors.

e TPSA: topological polar surface area.

f NRB: number of rotatable bonds.

According to the calculated parameters, all selected compounds were demonstrated to completely adhere to Lipinski’s rule of five that points to the following requirements for the oral bioavailability of new drug candidates: MW ≤ 500, log *P* ≤ 5, HBD ≤ 5, and HBA ≤ 10. To ensure minimal conformational changes during interactions with biological targets and to optimize oral bioavailability, the number of rotatable bonds needs to be limited to less than 10 as a significant descriptor of molecular flexibility. In adherence to this criterion, all selected molecules possess ≤10 rotatable bonds. Furthermore, our compounds fall within the acceptable limit of TPSA as numerous drug molecules utilized in the clinic have TPSA below 140–150 Å^2^.^32^

#### 2.3.2 Molecular Docking

**QST4** was found to be the most effective antitubercular agent in this series; therefore, we aimed to suggest a potential mechanism of action by applying molecular docking studies. The enoyl-acyl carrier protein (ACP) reductase, also known as InhA, is an enzyme that plays a crucial role in the biosynthesis of mycolic acids in *M. tuberculosis*. Mycolic acids constitute significant components of the cell envelope of mycobacteria. As InhA assumes fundamental importance for sustaining the growth of *M. tuberculosis*, it is regarded as an appealing target for identifying new antitubercular agents.^33^

**Figure 6:**
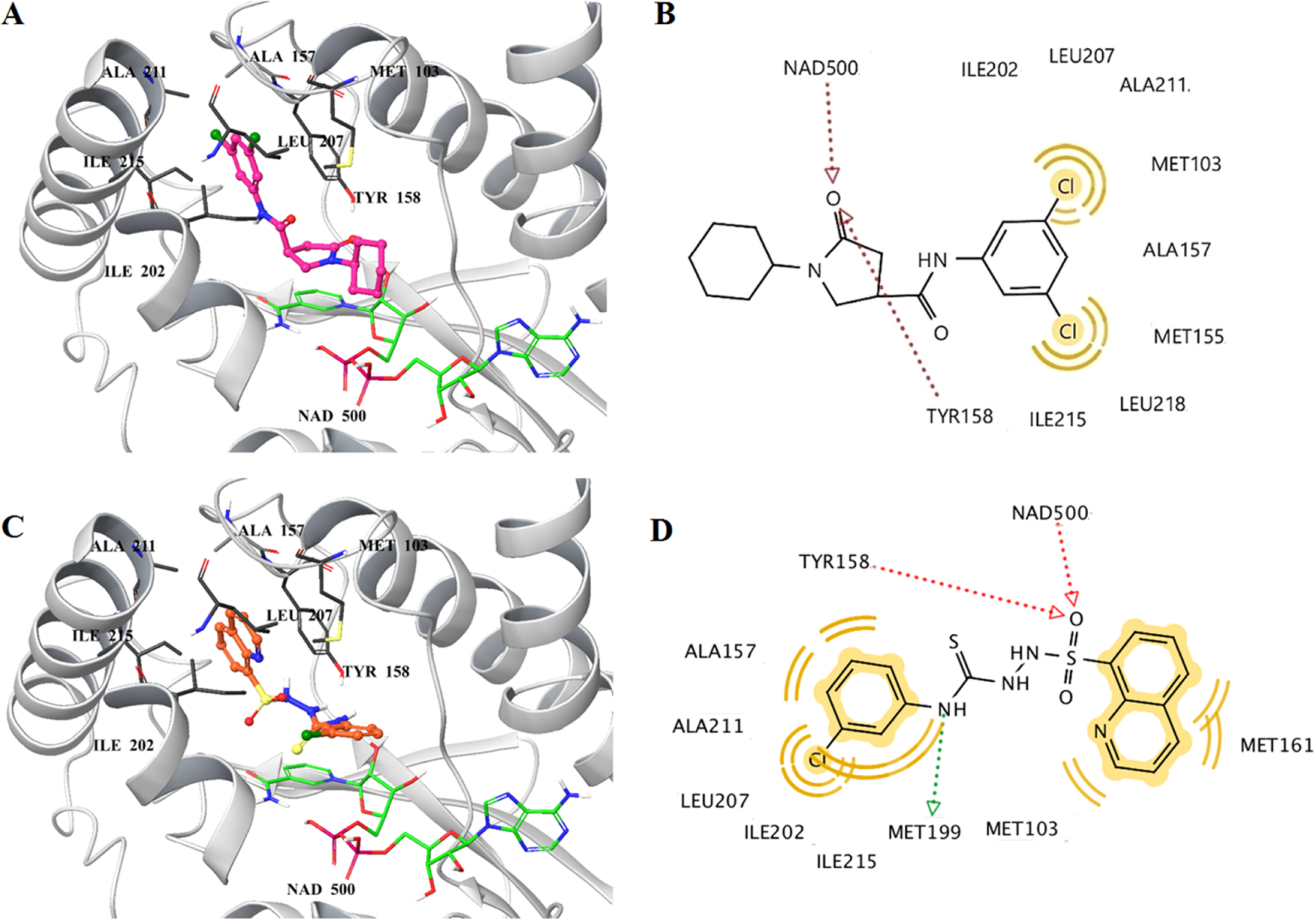
3D view of the cocrystallized inhibitor (pink balls and sticks) in 4TZK^35^ (A) and the most plausible binding mode of **QST4** (orange balls and sticks) in the binding site of InhA (C). The protein backbone and common amino acids participating in the interactions of both ligands with InhA are represented as white cartoons and gray sticks, respectively. (B) and (D) represent 2D depictions of enzyme–ligand interactions: Hydrogen bond acceptors, red arrows; hydrogen bond donor, green arrow; and yellow spheres, hydrophobic interactions.

Despite the availability of chemically diverse molecules that can occupy the binding site of InhA, in most cases, direct inhibitors of InhA interact with both cofactor NADH and a specific tyrosine residue (Tyr158) via hydrogen bonds. Furthermore, terminal hydrophobic groups of such inhibitors also form lipophilic contacts within the binding pocket of InhA.^34^ Prompted by these considerations, we docked **QST4** into the binding pocket of InhA due to pharmacophore similarity (terminal hydrophobic rings and central groups that are capable of forming hydrogen bonds) between known direct InhA inhibitors and **QST4** (Figure 6).

Furthermore, we subjected the cocrystallized ligand in the crystal structure of 4TZK to a redocking process and compared the binding pose with its initial positioning through evaluation of the root-mean-square deviation (RMSD). By obtaining this value as 0.48, we verified the reliability of our docking parameters. Upon analysis of the docking poses for both ligands, it was observed that the cocrystallized ligand in 4TZK binds to the active site of InhA through two hydrogen bonds to NAD and Tyr158. Furthermore, this ligand participates in hydrophobic interactions within the lipophilic pockets of the enzyme. Afterward, we examined the most plausible binding mode of **QST4** within the same pocket. It was observed that **QST4** occupied the binding site in the same way as the native ligand. Consistent with previously reported direct InhA inhibitors in the literature, **QST4** established two critical hydrogen bonds with NAD500 and Tyr158 through its sulfonyl group. Terminal lipophilic rings, quinoline and 3-chlorophenyl, also participated in the formation of hydrophobic contacts, as expected. Furthermore, an additional hydrogen bond was observed between Met199 and the N–H group of the thiosemicarbazide functionality. We additionally compared the binding energies (kcal/mol) of the cocrystallzed inhibitor of 4TZK and **QST4**. The estimated binding energy of **QST4** was calculated as −8.30 kcal/mol, whereas it was found as −10.78 kcal/mol for the original inhibitor of the InhA enzyme. These binding scores indicate that **QST4** can target the active site of InhA, which is occupied by the inhibitor but does not interact with this binding pocket as strongly as the cocrystallized ligand.

Based on molecular docking studies, **QST4** is expected to demonstrate its antimycobacterial activity through the direct inhibition of the InhA enzyme.

### 2.4 DFT Studies

DFT is used as an excellent theoretical method to identify kinetic and thermodynamic stability and for structural computations, mechanistic insights, molecular interactions, as well as optical and electronic properties of atoms and the molecular system.^36−41^ All theoretical calculations were carried out with the software package Gaussian 09.^42^ Geometry optimizations of the compounds have been performed using DFT at the B3LYP/6-31+ G(d,p) level of theory in the gas phase.^43,44^ Gauss View Rev. 5.0^45^ and CYLview visualization software^46^ were used to prepare and visualize the results.

#### 2.4.1 Molecular Structure and Stability

All compounds were optimized at the B3LYP/6-31+G(d,p) level of the DFT method in the gas phase. The optimized structures of the molecules give us the possible structural properties of their most stable states. It can also provide important insights into the strength of intramolecular hydrogen bonds. Using these structural parameters and energy values, we compared the strength of intramolecular hydrogen bonds in the compounds and examined the stability of the structural isomer compounds.

The compounds **QST1**–**QST14** are stabilized by intramolecular interactions and intramolecular hydrogen bonds containing N20–H21···N19, N22–H23···N20, N20–H21···N22, and N20–H21···S27. In addition, **QST6**, **QST8**, and **QST11** are stabilized by intramolecular hydrogen bonds containing N25–H26···F38, N25–H26···F40, and N25–H26···O38, respectively. For **QST6, QST8**, and **QST11**, the distances of F38···H26, F40···H26 and O38···H26 are calculated as 2.136, 2.105, and 2.046 Å, respectively (Figure S2). The optimized geometries and some selected structural parameters of **QST6**, **QST8**, and **QST11** are presented in the Supporting Information (Figure S2).

**Figure 7:**
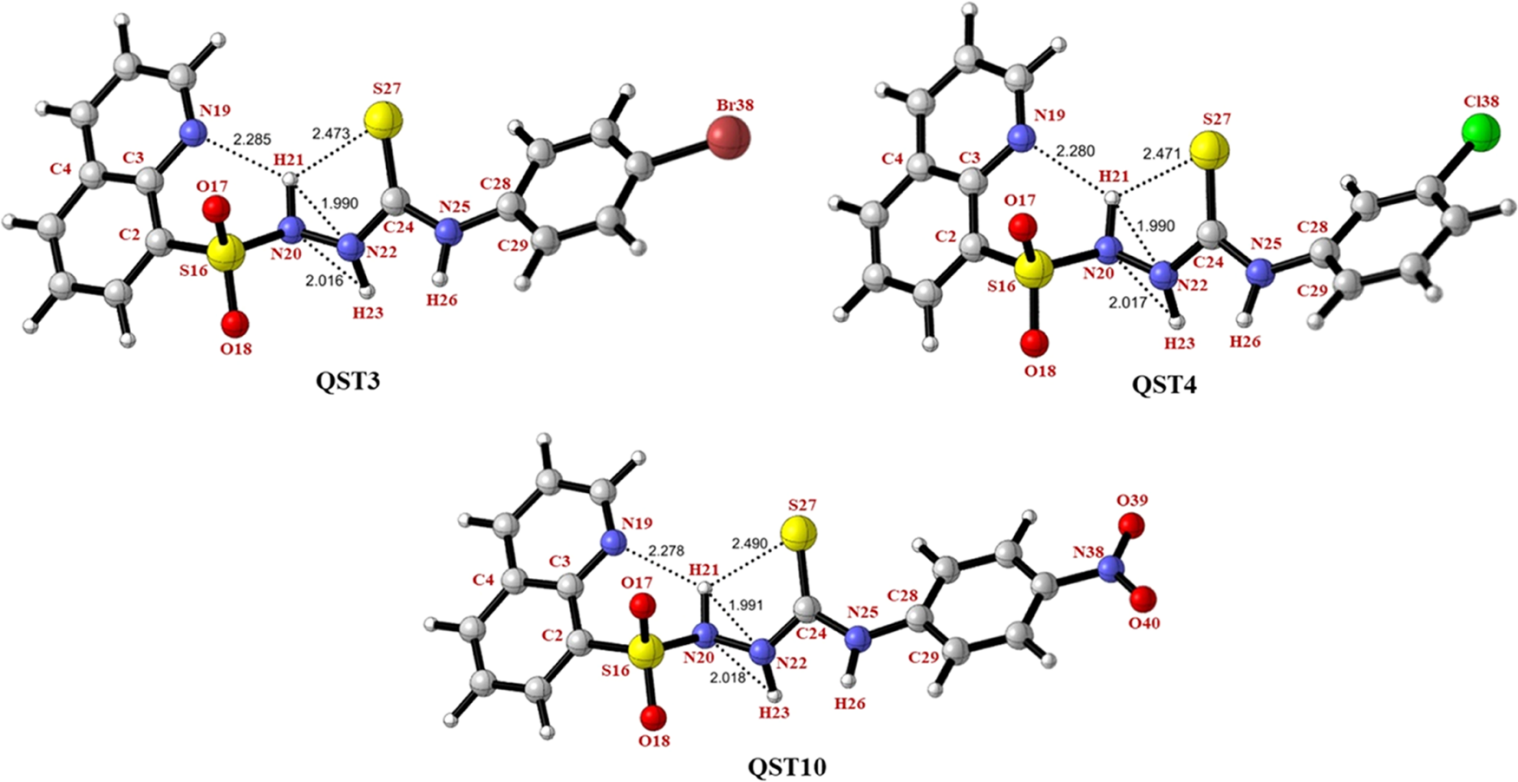
Optimized geometries and some selected structural parameters of **QST3**, **QST4**, and **QST10** using the B3LYP/6-31+G(d,p) basis set.

For **QST3, QST4**, and **QST10**, the distances of H21···S27 and H21···N19 are calculated as 2.473, 2.471, and 2.490 Å and 2.285, 2.280, and 2.278 Å, respectively (Figure 7). Similar hydrogen bond parameters were observed for all compounds (**QST1–QST14)** (Table S2). The strength of H-bonds depends on their geometry as well as their distance. It was determined that although the distance S27···H21 was longer than N19···H21, N20···H23, and N22···H21 for all compounds, the sulfur-containing H-bond was stronger than the H-bonds formed by N19, N20, and N22. Here, it is quite surprising that although the electronegativity of sulfur (2.58) according to the Pauling scale^26,47^ is lower than that of fluorine (3.98), oxygen (3.44), and nitrogen (3.04), it forms stronger H-bonds than these atoms. The strengths of these H-bonds are examined in more detail in the NBO analysis section.

The hydrogen-bonding parameters and Cartesian coordinates of all optimized compounds (**QST1**–**QST14**) are found in the Supporting Information (Tables S2 and S6, respectively). Since compounds **QST4**–**QST5**, **QST6**–**QST7**, and **QST11**–**QST12** are structural isomers, the relative total energies of these compounds were calculated and their stability was examined (Figure S1 and Table S1). These energy values show that compounds **QST5**, **QST6**, and **QST11** are more stable than **QST4**, **QST7**, and **QST12** by 0.028, 0.285, and 1.746 kcal/mol, respectively. The presence of intramolecular hydrogen bonds between the fluorine atom (F38) in **QST6** and the oxygen atom (O38) in **QST11** and the hydrogen atom (H26) attached to the nitrogen atom (N25) makes these compounds more stable than compounds **QST7** and **QST12**, respectively.

In molecular docking studies for **QST4**, it was observed that a hydrogen bond was formed between Met199 and H26 attached to the nitrogen atom (N25) in the thiosemicarbazide functional group. The formation of intramolecular hydrogen bonds is likely to be one of the factors determining the biological profile of this class of compounds. This situation can reduce the probability of the nitrogen atom (N25) of the thiosemicarbazide functionality forming hydrogen bonds with the potential biological targets. The stabilization energies of these hydrogen bonds, which may possibly affect the antimicrobial activity, were calculated and examined in the NBO Calculations section using the B3LYP/6-31+G(d,p) basis set.

#### 2.4.2 NBO Calculations

Mostly involving charge density and transfer on atoms, hydrogen bonds, and hyperconjugative interactions, NBO analysis is a tremendous computational tool for calculating interactions between molecules and atoms.^48−50^ We have investigated intramolecular N–H···N, N–H···S, N–H···F, and N–H···O hydrogen-bonding and hyperconjugative interactions by NBO calculations. The donor–acceptor interaction energies of all compounds (**QST1–QST14)** are found in the Supporting Information (Table S3).

It can be clearly seen in Table S3 that the strongest stabilization energies for hydrogen bonds in **QST1–QST14** have occurred from LP(2) S27 and antibonding orbital σ* N20H21. The intramolecular hydrogen bonds N20–H21···S27 containing the sulfur for **QST1–QST14** are stronger than the hydrogen bonds N20–H21···N19, N22–H23···N20, N20–H21···N22, N25–H26···F38 (for **QST6)**, N25–H26···F40 (for **QST8)**, and N25–H26···O38 (for **QST11)** formed by nitrogen, oxygen, and fluorine.

The strongest stabilization energies for the hydrogen bonds in **QST3**, **QST4**, and **QST10** have occurred from the LP(2) S27 to antibonding orbital σ* N20H21 with energies of 6.25, 6.28, and 6.56 kcal/mol, respectively. In Table 3, the strongest stabilization energy for intramolecular interactions in **QST3**, **QST4**, and **QST10** has occurred from the LP(3) O17 to antibonding orbital σ* S16N20 with an energy of 30.65, 30.64, and 30.72 kcal/mol, respectively. All charges on the atoms by NBO analysis calculated at the B3LYP/6-31+G(d,p) level of theory are given in the Supporting Information (Table S4). The most electronegative atoms O17, O18, N19, N20, N22, N25, and S27 for **QST3** have charged a value of −0.897, −0.951, −0.459, −0.710, −0.497, −0.626, and −0.162, respectively. Although sulfur (S27) has the lowest charge distribution (−0.162) among these electronegative atoms, the strongest stabilization energy has occurred from the LP(2) S27 to antibonding orbital σ* N20H21 for all of the compounds (**QST1–QST14**).

**Table 3:** Selected NBO Donor–Acceptor Interactions for the H-Bonds and Intramolecular Interactions of **QST3**, **QST4**, and **QST10** Using the B3LYP/6-31+G(d,p) Basis Set

donor NBO (i)	acceptor NBO (*j*)	E^(2)^^a^ kcal/mol	*E*(*j*) – *E*(*i*)^b^ a.u.	*F*(*i*,*j*)^c^ a.u.
**QST3**				
LP(2) O17	σ* C2S16	17.94	0.43	0.078
LP(2) O17	σ* S16O18	15.71	0.55	0.084
LP(3) O17	σ* S16N20	30.65	0.35	0.094
LP(2) O18	σ* S16O17	22.50	0.56	0.101
LP(3) O18	σ* C2S16	14.12	0.44	0.070
LP(3) O18	σ* S16N20	19.18	0.36	0.076
LP(1) N19	σ* C3C4	10.49	0.87	0.086
LP(1) N19	σ* C11C12	9.95	0.88	0.085
LP(1) N19	σ* N20H21	4.59	0.78	0.054
LP(1) N20	σ* S16O17	7.52	0.66	0.064
LP(1) N20	σ* N22H23	1.19	0.79	0.028
LP(1) N22	σ* N20H21	1.39	0.72	0.030
LP(1) N25	π* C28C29	27.29	0.29	0.082
LP(1) S27	σ* N20H21	0.93	1.12	0.029
LP(2) S27	σ* N20H21	6.25	0.62	0.057
LP(2) S27	σ* C24N25	12.43	0.62	0.080
**QST4**				
LP(2) O17	σ* C2S16	17.85	0.43	0.078
LP(2) O17	σ* S16O18	15.86	0.55	0.085
LP(3) O17	σ* S16N20	30.64	0.35	0.094
LP(2) O18	σ* S16O17	22.49	0.56	0.101
LP(3) O18	σ* C2S16	14.29	0.44	0.071
LP(3) O18	σ* S16N20	18.93	0.36	0.076
LP(1) N19	σ* C3C4	10.49	0.87	0.086
LP(1) N19	σ* C11C12	9.94	0.88	0.085
LP(1) N19	σ* N20H21	4.69	0.78	0.054
LP(1) N20	σ* S16O17	7.56	0.66	0.064
LP(1) N20	σ* N22H23	1.16	0.79	0.027
LP(1) N22	σ* N20H21	1.30	0.72	0.029
LP(1) N25	π* C28C29	24.12	0.30	0.077
LP(1) S27	σ* N20H21	0.93	1.12	0.029
LP(2) S27	σ* N20H21	6.28	0.62	0.057
LP(2) S27	σ* C24N25	12.66	0.62	0.081
**QST10**				
LP(2) O17	σ* C2S16	17.88	0.43	0.078
LP(2) O17	σ* S16O18	15.66	0.55	0.084
LP(3) O17	σ* S16N20	30.72	0.35	0.094
LP(2) O18	σ* S16O17	22.49	0.56	0.101
LP(3) O18	σ* C2S16	14.20	0.43	0.070
LP(3) O18	σ* S16N20	19.08	0.36	0.076
LP(1) N19	σ* C3C4	10.48	0.87	0.086
LP(1) N19	σ* C11C12	9.94	0.88	0.085
LP(1) N19	σ* N20H21	4.55	0.77	0.053
LP(1) N20	σ* S16O17	7.57	0.66	0.064
LP(1) N20	σ* N22H23	1.21	0.79	0.028
LP(1) N22	σ* N20H21	1.38	0.72	0.030
LP(1) N25	π* C28C29	24.48	0.29	0.077
LP(1) S27	σ* N20H21	0.99	1.12	0.030
LP(2) S27	σ* N20H21	6.56	0.62	0.058
LP(2) S27	σ* C24N25	12.57	0.62	0.081

a *E*^(2)^ means energy of hyperconjugative interactions (stabilization energy).

b Energy difference between donor and acceptor *i* and *j* NBO orbitals.

c *F*(*i*, *j*) is the Fork matrix element between *i* and *j* NBO orbitals.

#### 2.4.3 Frontier Molecular Orbital (FMO) Analysis

**Figure 8:**
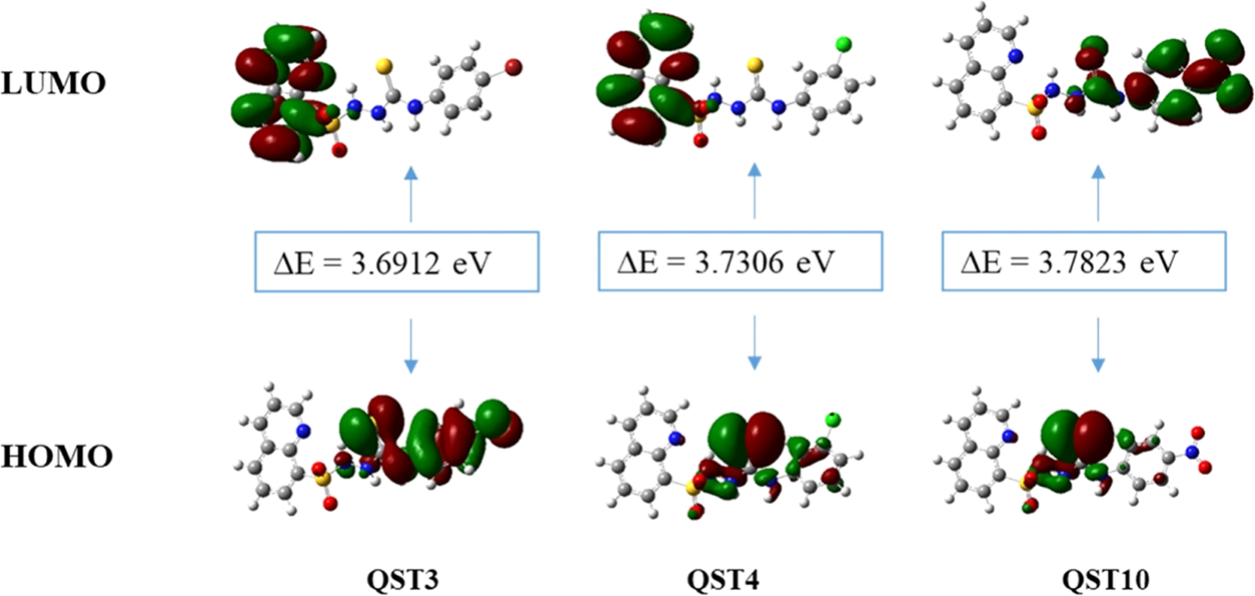
Frontier molecular orbitals of **QST3**, **QST4**, and **QST10** using the B3LYP/6-31+G(d,p) basis set.

The energy of frontier molecular orbitals is often used to make comparisons with the chemical stability of molecules. A molecule with a large HOMO–LUMO energy gap (Δ*E*) is associated with a low chemical reactivity and high kinetic stability. The smaller the energy gap between the HOMO and the LUMO, the easier the electronic charge transfer from the donor groups to the withdrawing groups, and the molecule is called a soft molecule, as it is highly polarized. When the energy gap is high, it is called a hard molecule; its ability to react is low, and it is more stable. The frontier energy gaps of **QST3**, **QST4**, and **QST10** are calculated as 3.6912, 3.7306, and 3.7823 eV, respectively (Figure 8).

In the case of HOMO, while the charge density for **QST3** is accumulated in the thiourea group and 4-bromophenyl group, for **QST4**, the charge density is mainly accumulated in the thiourea group, and there is a very small contribution on the phenyl ring. However, in the case of LUMO, the charge densities for both **QST3** and **QST4** are spread out only on the quinoline ring. On the other hand, in the case of HOMO, while the charge density for **QST10** is almost completely accumulated on the thiourea group, there is a very small charge on the phenyl ring. In the case of LUMO, unlike **QST3** and **QST4**, the charge density is spread from the thiourea group through the phenyl group, mostly toward the nitro group.

HOMO–LUMO visualizations and energy values for **QST1**–**QST14** are given in the Supporting Information in Figure S3 and Table S5, respectively. While **QST9** has the highest energy gap with a value of 3.9021 eV, **QST13** has the lowest energy gap with a value of 3.3581 eV. When the energy gaps in Table S5 are compared, the electron-donating ethyl group attached to the nitrogen atom in **QST13** or the presence of electron-donating groups at 2 or 4 positions in the phenyl ring in **QST11** (Δ*E* = 3.4852 eV) and **QST12** (Δ*E* = 3.4389 eV) can reduce the energy gap. On the other hand, in **QST9** and **QST6**, the presence of electron-withdrawing groups (–CF_3_ in **QST9**) or atoms (–F in **QST6)** in the phenyl ring also increases the energy gap considerably, reducing its chemical reactivity and making it more stable.

The quantum chemical reactivity descriptors are often used widely to make predictions about the chemical behavior of molecules. According to the Koopmans theorem,^51^ the frontier orbital energies HOMO and LUMO are related to ionization energy (*I* = −HOMO) and electron affinity (*A* = −LUMO). Several quantum chemical reactivity identifiers such as ionization potential (*I*), electron affinity (*A*), global chemical hardness (η), global softness (σ), electronegativity (χ), electrophilicity (ω), and chemical potential (μ) have been calculated by using the following equations^52−54^
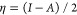

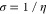

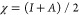

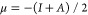

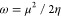


These parameters for all compounds (**QST1–QST14**) are given in Table S5.

#### 2.4.4 Molecular Electrostatic Potential (MEP)

**Figure 9:**
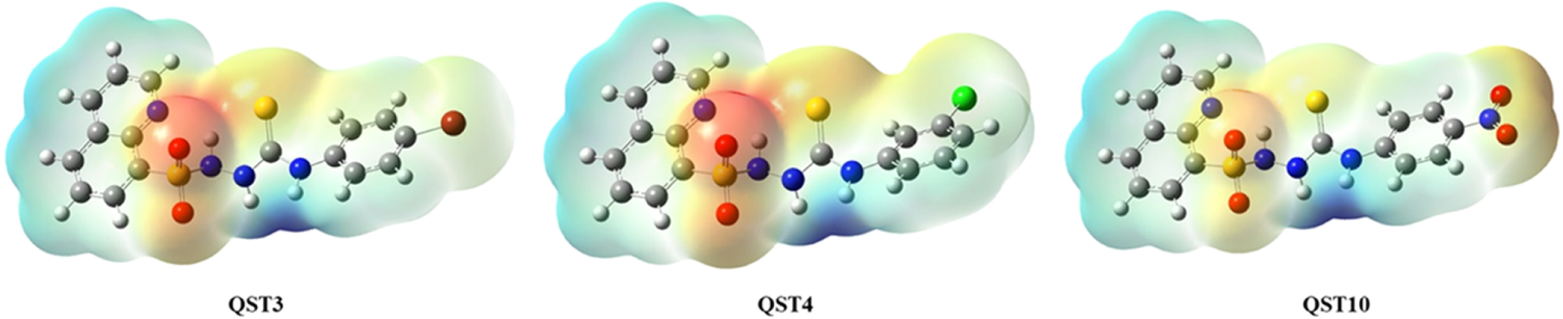
MEP surfaces of **QST3**, **QST4**, and **QST10** using the B3LYP/6-31+G(d,p) basis set.

MEP images provide information about the hydrogen bond interactions and the biological recognition process and are also widely used to make important inferences about the interpretation of electrophilic and nucleophilic reactions.^55,56^ Moreover, MEP surface results help predict the reactivity of a molecule by mapping its surface of positive, negative, and neutral electrostatic potential. Figure 9 shows the MEP surfaces of the most active compounds, **QST3**, **QST4**, and **QST10**. In the MEP plots, the blue color depicts the most positive regions being electron-poor, while the red color shows the most negative regions being electron-rich in the molecule.^57^ The MEP images of **QST1**–**QST14** show that the red-colored regions (negative) are mainly positioned around the sulfonyl group and, to a lesser extent, the thiono group, as well as for **QST10** around the nitro group (Figure S4). The electron-poor blue regions are located on the phenyl and quinoline rings, as well as the region of H23 and H26. When the molecular docking studies for **QST4** given in Figure 6 were examined, two hydrogen bonds were observed with Met199 and Tyr158 via the sulfonyl group, and another hydrogen bond was observed between Met199 and the N–H group of the thiosemicarbazide functional group. In MEP images, dark red and dark blue regions, which are likely to interact, are clearly seen. The MEP surfaces for all compounds (**QST1**–**QST14)** are given in the Supporting Information (Figure S4).

## 3 Conclusions

In the present study, we utilized the molecular hybridization approach to design 14 novel compounds that contain both quinoline scaffold and thiosemicarbazide pharmacophores. The target molecules were synthesized by the reaction of quinoline-8-sulfonohydrazide and various isothiocyanates. Extensive screening of the compounds for their antimicrobial activity against mycobacterial and bacterial strains as well as against the fungal opportunistic pathogen *Candida albicans* presented the antimicrobial potential of some of the molecules in this series. Additionally, DFT studies, including structural calculations, FMO, NBO, MEP, and quantum chemical reactivity descriptors, were performed to examine the presence and nature of intramolecular hydrogen bond interactions and their chemical stability and reactivity in all compounds. In the MEP images, the dark red and dark blue regions, which are highly likely to interact, support the hydrogen bonds observed between the sulfonyl group and the N–H group of the thiosemicarbazide functional group and Met199 and Tyr158 in molecular docking studies. All compounds were optimized by the DFT method using B3LYP with the 6-31+G(d,p) basis set. We found that in all of these synthesized compounds, intramolecular sulfur-containing hydrogen bonds formed had higher stabilization energy than H-bonds formed by nitrogen, oxygen, and fluorine. The unprecedented important findings and bioactivity data obtained thus provide significant insights, encouraging and enabling further studies on quinoline-based thiosemicarbazides.

## 4 Experimental Section

### 4.1 Chemistry

#### 4.1.1 Materials and Methods

The starting materials and reagents were purchased from commercial sources and used without further purification. Toluene was distilled from sodium-benzophenone just prior to use. ^1^H NMR (400 MHz) and ^13^C NMR (100 MHz) spectra were recorded on a Bruker AM, using SiMe_4_ as an internal reference in DMSO-*d*_6_. The ^15^N NMR spectrum was recorded on a Bruker AvanceCore 400 MHz instrument in DMSO-*d*_6_. High-resolution mass spectra were obtained using an Agilent G6530B Q-TOF spectrometer (Atatürk University-East Anatolian High Technology Research and Application Center (DAYTAM)), and are reported for M + H. Reaction time and purity of the products were determined by thin-layer chromatography (TLC) with a fluorescent indicator visualizable at 254 and 365 nm. Infrared (IR) spectra were recorded in the range 4000–600 cm^–1^ via ATR diamond.

#### 4.1.2 Synthetic Procedures

##### 4.1.2.1. Preparation of Quinoline-8-sulfonohydrazide (**II**)

Quinoline-8-sulfonohydrazide (**II**) was synthesized according to the following procedure: A solution of quinoline-8-sulfonyl chloride (1 equiv) in tetrahydrofuran was added dropwise to hydrazine monohydrate (2.5 equiv) at 0 °C. The reaction mixture was then stirred for 30 min at 0 °C and ethyl acetate was added to the reaction mixture. The organic layer was washed with saturated brine (3 × 5 mL) and dried over sodium sulfate. After the concentration of the mixture under a vacuum, the residue was dissolved in chloroform and precipitated in hexane, giving quinoline-8-sulfonohydrazide (**II**). The desired quinoline-8-sulfonohydrazide (**II**) was used without further purification. ^1^H NMR (400 MHz, DMSO-*d*_6_) δ 9.08 (dd, *J* = 4.2, 1.8 Hz, 1H), 8.58 (dd, *J* = 8.3, 1.7 Hz, 1H), 8.35 (dd, *J* = 7.3, 1.3 Hz, 1H), 8.32 (dd, *J* = 8.2, 1.3 Hz, 1H), 8.04 (s, 1H), 7.90–7.59 (m, 2H), 4.49 (s, 2H). The analysis of spectral data (^1^H NMR) of quinoline-8-sulfonohydrazide (**II**) is presented in the Supporting Information.

##### 4.1.2.2. General Procedure for *N*-Substituted-2-(quinolin-8-ylsulfonyl)hydrazine-1-carbothioamid Derivatives (**QST1–QST14**)

Quinoline-8-sulfonohydrazide (**II**) was added to an equimolar amount of corresponding isothiocyanate solutions in 10 mL of dry toluene. The mixture was continuously stirred at 80 °C and then cooled to room temperature after completion of the reaction (TLC monitored).

The solid that was obtained was filtered and washed with toluene. The resulting residue was purified by crystallization from ethanol to afford the target compounds **QST1–QST14.** The analysis of spectral data (^1^H and ^13^C NMR) of **QST1**–**QST14** is presented in the Supporting Information.

##### 4.1.2.3. *N*-Phenyl-2-(quinolin-8-ylsulfonyl)hydrazine-1-carbothioamide (**QST1**)

Yellow solid, yield: 30%. Mp 181–183 °C; *R*_f_ (EtOAc:methanol = 9:1): 0.78. IR (υ, cm^–1^) 3314, 2961 (NH), 1209 (C=S), 1319, 1167 (SO_2_). ^1^H NMR (400 MHz, DMSO-*d*_6_) δ 10.07 (s, 1H), 9.89 (s, 1H), 9.81 (s, 1H), 9.08–8.93 (m,1H), 8.57 (d, *J* = 8.3 Hz, 1H), 8.40 (dd, *J* = 16.1, 7.7 Hz, 2H), 7.85–7.76 (m, 1H), 7.71 (dd, *J* = 8.3, 4.3 Hz, 1H), 7.50–7.26 (m, 4H), 7.15 (t, *J* = 7.1 Hz, 1H). ^13^C NMR (100 MHz, DMSO-*d*_6_) δ 181.5, 151.9, 143.6, 139.2, 137.8, 135.3, 135.2, 133.0, 129.3, 128.5, 126.2, 125.6, 125.4, 123.1. HRMS (EI): *m*/*z*: C_16_H_15_N_4_O_2_S_2_ [M + H]^+^, calculated 359.0636; found 359.0626.

##### 4.1.2.4. 2-(Quinolin-8-ylsulfonyl)-*N*-(p-tolyl)hydrazine-1-carbothioamide (**QST2**)

White solid, yield: 77%. Mp 167–168 °C; *R*_f_ (EtOAc:methanol = 9:1): 0.80. IR (υ, cm^–1^) 3340, 3086 (NH), 1211 (C=S), 1324, 1163 (SO_2_). ^1^H NMR (400 MHz, DMSO-*d*_6_) δ 10.01 (s, 1H), 9.82 (s, 1H), 9.79 (s, 1H), 9.01 (s, 1H), 8.66–8.54 (m, 1H), 8.51–8.27 (m, 2H), 7.92–7.78 (m, 1H), 7.77–7.66 (m, 1H), 7.21 (bs, 2H), 7.12 (bs, 2H), 2.28 (s, 3H). ^13^C NMR (100 MHz, DMSO-*d*_6_) δ 181.5, 151.9, 143.6, 137.8, 136.6, 135.3, 135.1, 134.8, 133.1, 129.3, 129.0, 126.2, 125.5, 123.1, 21.0. HRMS (EI): *m*/*z*: C_17_H_17_N_4_O_2_S_2_ [M + H]^+^, calculated 373.0793; found 373.0770.

##### 4.1.2.5. *N*-(4-Bromophenyl)-2-(quinolin-8-ylsulfonyl)hydrazine-1-carbothioamide (**QST3**)

Pale yellow solid, yield: 57%. Mp 193–194 °C; *R*_f_ (EtOAc:methanol = 9:1): 0.80. IR (υ, cm^–1^) 3296, 3141 (NH), 1215 (C=S), 1336, 1143 (SO_2_). ^1^H NMR (400 MHz, DMSO-*d*_6_) δ 10.14 (s, 1H), 10.04 (s, 1H), 9.78 (s, 1H), 9.04 (s, 1H), 8.63–8.52 (m, 1H), 8.53–8.31 (m, 2H), 7.89–7.66 (m, 2H), 7.50 (d, *J* = 8.3 Hz, 2H), 7.37 (d, *J* = 8.4 Hz, 2H). ^13^C NMR (100 MHz, DMSO-*d*_6_) δ 181.6, 151.9, 143.6, 138.7, 137.8, 135.3, 135.1, 133.0, 131.3, 129.3, 127.6, 126.2, 123.1, 117.9. HRMS (EI): *m*/*z*: C_16_H_14_BrN_4_O_2_S_2_ [M + H]^+^, calculated 436.9742; found 436.9726.

##### 4.1.2.6. *N*-(3-Chlorophenyl)-2-(quinolin-8-ylsulfonyl)hydrazine-1-carbothioamide (**QST4**)

Pale green solid, yield: 40%. Mp 181–182 °C; *R*_f_ (EtOAc:methanol = 9:1): 0.78. IR (υ, cm^–1^) 3335, 3106 (NH), 1215 (C=S), 1327, 1144 (SO_2_). ^1^H NMR (400 MHz, DMSO-*d*_6_) δ 10.20 (s, 1H), 10.04 (s, 1H), 9.80 (s, 1H), 9.05 (s, 1H), 8.58 (d, *J* = 7.5 Hz, 1H), 8.51–8.30 (m, 2H), 7.91–7.77 (m, 1H), 7.73 (dd, *J* = 7.5, 3.6 Hz, 1H), 7.52 (s, 1H), 7.44–7.30 (m, 2H), 7.27–7.16 (m, 1H). ^13^C NMR (100 MHz, DMSO-*d*_6_) δ 181.5, 151.9, 143.6, 140.7, 137.8, 135.3, 135.1, 132.9, 132.5, 130.1, 129.3, 126.2, 125.3, 124.9, 123.9, 123.1. HRMS (EI): *m*/*z*: C_16_H_14_ClN_4_O_2_S_2_ [M + H]^+^, calculated 393.0247; found 393.0223.

##### 4.1.2.7. *N*-(4-Chlorophenyl)-2-(quinolin-8-ylsulfonyl)hydrazine-1-carbothioamide (**QST5**)

Yellow solid, yield: 52%. Mp 188–189 °C; *R*_f_ (EtOAc:methanol = 9:1): 0.82. IR (υ, cm^–1^) 3300, 3136 (NH), 1216 (C=S), 1336, 1164 (SO_2_). ^1^H NMR (400 MHz, DMSO-*d*_6_) δ 10.14 (s, 1H), 10.04 (s, 1H), 9.78 (s, 1H), 9.04 (s, 1H), 8.58 (d, *J* = 8.1 Hz, 1H), 8.50–8.31 (m, 2H), 7.85–7.77 (m, 1H), 7.77–7.69 (m, 1H), 7.55–7.29 (m, 4H). ^13^C NMR (100 MHz, DMSO-*d*_6_) δ 181.6, 151.9, 143.6, 138.3, 137.8, 135.3, 135.1, 133.0, 129.6, 129.3, 128.4, 127.3, 126.2, 123.1. HRMS (EI): *m*/*z*: C_16_H_14_ClN_4_O_2_S_2_ [M + H]^+^, calculated 393.0247; found 393.0230.

##### 4.1.2.8. *N*-(2-Fluorophenyl)-2-(quinolin-8-ylsulfonyl)hydrazine-1-carbothioamide (**QST6**)

Pale yellow solid, yield: 87%. Mp 178–179 °C; *R*_f_ (EtOAc:methanol = 9:1): 0.90. IR (υ, cm^–1^) 3303, 2953 (NH), 1220 (C=S), 1321, 1167 (SO_2_). ^1^H NMR (400 MHz, DMSO-*d*_6_) δ 10.20 (s, 1H), 9.88 (s, 1H), 9.80 (s, 1H), 9.01 (d, *J* = 3.0 Hz, 1H), 8.57 (d, *J* = 8.1 Hz, 1H), 8.40 (dd, *J* = 16.2, 7.3 Hz, 2H), 7.81 (t, *J* = 7.2 Hz, 1H), 7.71 (dd, *J* = 8.3, 4.2 Hz, 1H), 7.44 (t, *J* = 7.6 Hz, 1H), 7.33–7.20 (m, 2H), 7.16 (t, *J* = 7.3 Hz, 1H). ^13^C NMR (100 MHz, DMSO-*d*_6_) δ 182.5, 156.9 (d, *J* = 246.1 Hz), 151.9, 143.6, 137.8, 135.3, 135.0, 133.0, 129.4, 129.3, 128.2 (d, *J* = 7.6 Hz), 127.2 (d, *J* = 11.6 Hz), 126.2, 124.3, 123.1, 116.0 (d, *J* = 19.9 Hz). HRMS (EI): *m*/*z*: C_16_H_14_FN_4_O_2_S_2_ [M + H]^+^, calculated 377.0542; found 377.0539.

##### 4.1.2.9. *N*-(4-Fluorophenyl)-2-(quinolin-8-ylsulfonyl)hydrazine-1-carbothioamide (**QST7**)

White solid, yield: 50%. Mp 196–197 °C; *R*_f_ (EtOAc:methanol = 9:1): 0.78. IR (υ, cm^–1^) 3302, 2958 (NH), 1209 (C=S), 1319, 1169 (SO_2_). ^1^H NMR (400 MHz, DMSO-*d*_6_) δ 10.07 (s, 1H), 9.97 (s, 1H), 9.77 (s, 1H), 9.03 (s, 1H), 8.58 (d, *J* = 8.4 Hz, 1H), 8.40 (dd, *J* = 13.7, 7.5 Hz, 2H), 7.87–7.78 (m, 1H), 7.76–7.66 (m, 1H), 7.44–7.29 (m, 2H), 7.14 (t, *J* = 8.1 Hz, 2H). ^13^C NMR (100 MHz, DMSO-*d*_6_) δ 181.9, 159.9 (d, *J* = 241.8 Hz), 148.6, 143.6, 137.8, 135.6, 135.3, 135.1, 133.0, 129.3, 127.9 (d, *J* = 6.2 Hz), 126.2, 123.1, 115.2 (d, *J* = 22.5 Hz). HRMS (EI): *m*/*z*: C_16_H_14_FN_4_O_2_S_2_ [M + H]^+^, calculated 377.0542, found 377.0527.

##### 4.1.2.10. 2-(Quinolin-8-ylsulfonyl)-*N*-(2-(trifluoromethyl)phenyl)hydrazine-1-carbothioamide (**QST8**)

White solid, yield: 89%. Mp 190–191 °C; *R*_f_ (EtOAc:methanol = 9:1): 0.97. IR (υ, cm^–1^) 3351, 3089 (NH), 1213 (C=S), 1316, 1168 (SO_2_). ^1^H NMR (400 MHz, DMSO-*d*_6_) δ 10.30 (s, 1H), 9.97 (s, 1H), 9.69 (s, 1H), 8.94 (dd, *J* = 4.3, 1.7 Hz, 1H), 8.55 (dd, *J* = 8.4, 1.7 Hz, 1H), 8.47–8.43 (m, 1H), 8.43–8.37 (m, 1H), 7.83 (t, *J* = 7.8 Hz, 1H), 7.74–7.66 (m, 2H), 7.62 (t, *J* = 7.6 Hz, 1H), 7.45 (t, *J* = 7.7 Hz, 1H), 7.34 (d, *J* = 8.0 Hz, 1H). ^13^C NMR (100 MHz, DMSO-*d*_6_) δ 182.9, 151.9, 143.5, 137.8, 137.2, 135.4, 134.8, 133.3, 132.9, 131.5, 129.3, 127.6, 126.5, 126.2, 125.6, 125.3, 123.0. HRMS (EI): *m*/*z*: C_17_H_14_F_3_N_4_O_2_S_2_ [M + H]^+^, calculated 427.0510; found 427.0490.

##### 4.1.2.11. *N*-(3,5-Bis(trifluoromethyl)phenyl)-2-(quinolin-8-ylsulfonyl)hydrazine-1-carbothioamide (**QST9**)

White solid, yield: 90%. Mp 191–192 °C; *R*_f_ (EtOAc:methanol = 9:1): 0.97. IR (υ, cm^–1^) 3257, 3155 (NH), 1212 (C=S), 1353, 1169 (SO_2_).^1^H NMR (400 MHz, DMSO-*d*_6_) δ 10.52 (s, 1H), 10.44 (s, 1H), 9.83 (s, 1H), 9.07 (dd, *J* = 4.3, 1.7 Hz, 1H), 8.56 (dd, *J* = 8.4, 1.7 Hz, 1H), 8.47–8.35 (m, 2H), 8.26 (s, 2H), 7.85 (s, 1H), 7.84–7.78 (m, 1H), 7.71 (dd, *J* = 8.3, 4.3 Hz, 1H). ^13^C NMR (100 MHz, DMSO-*d*_6_) δ 181.7, 151.9, 143.6, 141.3, 137.7, 135.2, 135.1, 132.9, 130.2 (q, *J* = 32.6 Hz), 129.2, 126.1, 125.2, 123.0, 122.3, 118.2. HRMS (EI): *m*/*z*: C_18_H_13_F_6_N_4_O_2_S_2_ [M + H]^+^, calculated 495.0384; found 495.0376.

##### 4.1.2.12. *N*-(4-Nitrophenyl)-2-(quinolin-8-ylsulfonyl)hydrazine-1-carbothioamide (**QST10**)

Pale yellow solid, yield: 47%. Mp 194–195 °C; *R*_f_ (EtOAc:methanol = 9:1): 0.66. IR (υ, cm^–1^) 3318, 3141 (NH), 1210 (C=S), 1329, 1170 (SO_2_).^1^H NMR (400 MHz, DMSO-*d*_6_) δ 10.42 (s, 1H), 10.39 (s, 1H), 9.84 (s, 1H), 9.07 (s, 1H), 8.57 (d, *J* = 8.1 Hz, 1H), 8.53–8.31 (m, 2H), 8.18 (d, *J* = 8.7 Hz, 2H), 7.94–7.53 (m, 4H). ^13^C NMR (100 MHz, DMSO-*d*_6_) δ 181.3, 152.0, 145.7, 143.8, 143.6, 137.8, 135.3, 135.1, 132.9, 129.3, 126.2, 124.7, 124.2, 123.1. HRMS (EI): *m*/*z*: C_16_H_14_N_5_O_4_S_2_ [M + H]^+^, calculated 404.0487; found 404.0470.

##### 4.1.2.13. *N*-(2-Methoxyphenyl)-2-(quinolin-8-ylsulfonyl)hydrazine-1-carbothioamide (**QST11**)

White solid, yield: 52%. Mp 177–178 °C; *R*_f_ (EtOAc:methanol = 9:1): 0.87. IR (υ, cm^–1^) 3329, 3152 (NH), 1218 (C=S), 1337, 1169 (SO_2_). ^1^H NMR (400 MHz, DMSO-*d*_6_) δ 10.06 (s, 1H), 10.00 (s, 1H), 9.46 (s, 1H), 9.02 (s, 1H), 8.54 (d, *J* = 8.3 Hz, 1H), 8.47–8.30 (m, 2H), 8.22 (s, 1H), 7.89–7.75 (m, 1H), 7.76–7.66 (m, 1H), 7.17–7.10 (m, 1H), 7.04 (d, *J* = 8.1 Hz, 1H), 6.89 (t, *J* = 7.4 Hz, 1H), 3.81 (s, 3H). ^13^C NMR (100 MHz, DMSO-*d*_6_) δ 180.0, 152.1, 151.9, 150.8, 143.6, 137.6, 135.3, 134.8, 132.9, 129.2, 127.6, 126.1, 125.9, 123.0, 120.2, 111.6, 56.4. HRMS (EI): *m*/*z*: C_17_H_17_N_4_O_3_S_2_ [M + H]^+^, calculated 389.0742; found 389.0719.

##### 4.1.2.14. *N*-(4-Methoxyphenyl)-2-(quinolin-8-ylsulfonyl)hydrazine-1-carbothioamide (**QST12**)

White solid, yield: 83%. Mp 192–193 °C; *R*_f_ (EtOAc:methanol = 9:1): 0.83. IR (υ, cm^–1^) 3291, 3124 (NH), 1209 (C=S), 1337, 1164 (SO_2_).^1^H NMR (400 MHz, DMSO-*d*_6_) δ 9.97 (s, 1H), 9.81 (s, 1H), 9.78 (s, 1H), 9.00 (d, *J* = 4.0 Hz, 1H), 8.58 (d, *J* = 8.4 Hz, 1H), 8.43 (d, *J* = 7.3 Hz, 1H), 8.39 (d, *J* = 8.1 Hz, 1H), 7.82 (t, *J* = 7.7 Hz,1H), 7.72 (dd, *J* = 8.3, 4.3 Hz, 1H), 7.18 (d, *J* = 8.7 Hz, 2H), 6.87 (d, *J* = 8.7 Hz, 2H), 3.74 (s, 3H). ^13^C NMR (100 MHz, DMSO-*d*_6_) δ 181.8, 157.3, 151.9, 143.5, 137.8, 135.3, 135.2, 133.1, 131.9, 129.3, 127.3, 126.2, 123.1, 113.8, 55.7. HRMS (EI): *m*/*z*: C_17_H_17_N_4_O_3_S_2_ [M + H]^+^, calculated 389.0742; found 389.0735.

##### 4.1.2.15. *N*-(Naphthalen-1-yl)-2-(quinolin-8-ylsulfonyl)hydrazine-1-carbothioamide (**QST13**)

Pale yellow solid, yield: 57%. Mp 199–200 °C; *R*_f_ (EtOAc:methanol = 9:1): 0.84. IR (υ, cm^–1^) 3340, 3037 (NH), 1214 (C=S), 1326, 1166 (SO_2_).^1^H NMR (400 MHz, DMSO-*d*_6_) δ 10.35 (s, 1H), 10.15 (s, 1H), 9.92 (s, 1H), 9.04–8.81 (m, 1H), 8.56 (d, *J* = 7.1 Hz, 1H), 8.51 (d, *J* = 7.0 Hz, 1H), 8.40 (d, *J* = 8.0 Hz, 1H), 8.05–7.93 (m, 2H), 7.85 (t, *J* = 8.3 Hz, 2H), 7.66 (dd, *J* = 8.3, 4.3 Hz, 1H), 7.59–7.46 (m, 3H), 7.34 (d, *J* = 7.2 Hz, 1H). ^13^C NMR (100 MHz, DMSO-*d*_6_) δ 183.3, 151.8, 143.6, 137.8, 135.8, 135.3, 135.1, 134.2, 133.2, 130.8, 129.3, 128.3, 127.3, 126.6, 126.5, 126.4, 126.2, 125.8, 123.9, 123.0. HRMS (EI): *m*/*z*: C_20_H_17_N_4_O_2_S_2_ [M + H]^+^, calculated 409.0793; found 409.0772.

##### 4.1.2.16. *N*-Ethyl-2-(quinolin-8-ylsulfonyl)hydrazine-1-carbothioamide (**QST14**)

White solid, yield: 73%. Mp 198–199 °C; *R*_f_ (EtOAc:methanol = 9:1): 0.87. IR (υ, cm^–1^) 3335, 3143 (NH), 1209 (C=S), 1337, 1163 (SO_2_).^1^H NMR (400 MHz, DMSO-*d*_6_) δ 9.53 (s, 2H), 9.29–8.93 (m, 1H), 8.59 (d, *J* = 8.3 Hz, 1H), 8.37 (t, *J* = 7.1 Hz, 2H), 8.21 (t, *J* = 5.3 Hz, 1H), 7.90–7.59 (m, 2H), 3.49–3.39 (overlapped with solvent signal, 2H), 0.97 (t, *J* = 7.0 Hz, 3H). ^13^C NMR (100 MHz, DMSO-*d*_6_) δ 181.8, 151.9, 143.6, 137.7, 135.4, 135.1, 132.8, 129.3, 126.1, 123.1, 38.8, 14.6. HRMS (EI): *m*/*z*: C_12_H_15_N_4_O_2_S_2_ [M + H]^+^, calculated 311.0636; found 311.0617.

### 4.2 Evaluation of Antimicrobial Activity and Cytotoxicity

#### 4.2.1 Antitubercular Screening

*Mycobacterium tuberculosis* H37Rv and multidrug-resistant (*inhA* promoter mutant and *katG* S315T mutant) clinical isolates from Oslo University Hospital were streaked onto 7H10+OADC agar plates and incubated at 37 °C. In OADC-enriched liquid Sauton’s medium, pure colonies from agar plates were grown to the middle of the log phase. Subsequently, cultures were exponentially grown and inoculated into Sauton’s medium on 96-well plates at progressively higher concentrations of the testing chemicals, with approximately 4 × 10^5^ CFU/mL in 200 μL of each well. Before receiving 32.5 μL of a resazurin-Tween mixture (8:5 ratio of 0.6 mM resazurin in 1× PBS to 20% Tween 80), plates were incubated at 37 °C for 1 week. The production of fluorescent resorufin assists in determining the minimum inhibitory concentration (MIC) of the compounds tested.^58^

#### 4.2.2 Antibacterial and Antifungal Activity Determination

The minimum inhibitory concentration (MIC) values were determined according to the standard broth microdilution assays, recommended by the National Committee for Clinical Laboratory Standards for bacteria and by the European Committee on Antimicrobial Susceptibility Testing for *Candida* sp. Antimicrobial activity was tested against four bacterial strains: *Staphylococcus aureus* NCTC 6571, *Enterococcus faecium* ATCC 6057, *Pseudomonas aeruginosa* ATCC 10332, and *Klebsiella pneumonie* ATCC BAA 2146, and one *Candida* strain, *Candida albicans* ATCC 10231. For the cultivation of bacterial test organisms, Luria–Bertani broth was used, while for *C. albicans*, RPMI 1640 was used. Cultures were diluted to a concentration of 5 × 10^5^ colony forming units (CFU)/mL for bacteria and 1 × 10^5^ CFU/mL for *Candida* sp. All compounds were dissolved in DMSO and serially diluted in media to the concentration range of 3.91–500 μg/mL. As a positive control for bacterial strains, vancomycin (Acros Organics, Geel, Belgium) was used, while nystatin (Acros Organics, Geel, Belgium) was used for *C. albicans*. After incubation at 37 °C for 24 h, the minimum inhibitory concentrations were determined by measuring absorbance at 625 nm for bacterial species and 530 nm for *C. albicans* using a plate reader (Epoch Microplate Spectrophotometer, BioTek Instruments, Inc., SAD).

#### 4.2.3 Cytotoxicity Determination

To determine the toxicity of the compounds against normal host cells, we used the MTT 3-(4,5-dimethylthiazol-2-yl)-2,5-diphenyltetrazolium bromide assay. Cell toxicity was tested by using an inhibition assay with HEK (human embryonic kidney) cell lines. Test compounds at a 50 μM concentration were added to a sterile 96-well microtiter plate having 5 × 10^3^ cells and incubated for 48 h at 37 °C. After the incubation period, 10 μL of 3-(4,5-dimethylthiazol-2-yl)-2,5-diphenyltetrazolium bromide (MTT reagent) (5 mg/mL) was added and then incubated for 3 h. Next, the medium was removed, and 100 μL of DMSO was added to each well. DMSO dissolves the formazan crystals formed in wells. The absorbance was measured at 560 nm using a PerkinElmer Victor X3 microplate reader against the blank. The assay was performed in triplicate, and the cytotoxicity is represented as % inhibition at the test concentration.

### 4.3 Molecular modeling studies

*In silico* prediction of drug-likeness of the selected compounds was carried out in Molinspiration Cheminformatics free web services (www.molinspiration.com).^59^ For molecular docking studies, the crystal structure of *Mycobacterium tuberculosis* enoyl-ACP reductase (InhA) in complex with the inhibitor 1-cyclohexyl-N-(3,5-dichlorophenyl)-5-oxopyrrolidine-3-carboxamide was retrieved from the Protein Data Bank under the PDB code 4TZK.^35^ AutoDock 4.2^60^ integrated into LigandScout 4.4,^61^ using default parameters, was utilized to dock the most active *Mtb* growth inhibitor (**QST4**) into the binding site of InhA. The docking poses were visually analyzed and the figures belonging to the most plausible one were prepared using LigandScout and Maestro.^62^

## Supporting Information Available

^1^H NMR and ^13^C NMR of all synthesized compounds; Cartesian coordinates; HOMO–LUMO energy values; quantum chemical reactivity descriptors the MEPs and NBO charges for all compounds (**QST1**–**QST14**); relative total energies Δ*E* (kcal/mol) of **QST4**–**QST5**, **QST6**–**QST7**, and **QST11**–**QST12**; optimized geometries and some selected structural parameters of **QST6**, **QST8**, and **QST11**; and MIC values of the compounds against bacterial strains and *Candida albicans* (PDF)
