# Supplementary material for: Novel Quinoline-Based Thiosemicarbazide Derivatives: Synthesis, DFT Calculations, and Investigation of Antitubercular, Antibacterial, and Antifungal Activities
Source: ACS Omega. 2023 Oct 17;8(43):40140–52. doi: 10.1021/acsomega.3c03018 (PMC10620885; doi:10.1021/acsomega.3c03018)
Supplement: Supplementary file 1 — ao3c03018_si_001.pdf [file ao3c03018_si_001.pdf]

Novel quinoline-based thiosemicarbazide derivatives: Synthesis, DFT calculations, and investigation of antitubercular, antibacterial and antifungal activities

Esma Özcan,[a,b,1] Siva Krishna Vagolu,[c,1] Miyase Gözde Gündüz,[d] Milena Stevanovic,[e] Zülbiye Kökbudak,[a] Tone Tønjum,[c,f] Jasmina Nikodinovic-Runic,[e] Yasin Çetinkaya,[g*] Şengül Dilem Doğan[b*]

[a] Department of Chemistry, Faculty of Science, Erciyes University, 38039, Kayseri, Turkey

[b] Department of Basic Sciences, Faculty of Pharmacy, Erciyes University, 38039, Kayseri, Turkey

[c] Unit for Genome Dynamics, Department of Microbiology, University of Oslo, Oslo, Norway

[d] Department of Pharmaceutical Chemistry, Faculty of Pharmacy, Hacettepe University, Sıhhiye, 06100, Ankara, Turkey

[e] Institute of Molecular Genetics and Genetic Engineering, University of Belgrade, Belgrade, Serbia

[f] Unit for Genome Dynamics, Department of Microbiology, Oslo University Hospital, Oslo, Norway

[g] Department of Chemistry, Faculty of Science, Atatürk University, 25240, Erzurum, Turkey

*Corresponding authors
Dr. Şengül Dilem Doğan
Erciyes University, Faculty of Pharmacy
Department of Basic Sciences
38039 Kayseri, TURKEY
E-mail address: dogandilem@gmail.com
Phone number: +90 352 2076666-28032

Dr. Yasin Çetinkaya
Department of Chemistry, Faculty of Science,
Atatürk University, 25240 Erzurum, Turkey
E-mail address: yasin.cetinkaya@atauni.edu.tr
Phone: +90 442 2314389

[1] Equal contribution

Contents **Page**

Table S1. Total energies E_t (au) and relative total energies of **QST4-QST5**, **QST6-QST7** and **QST11-QST12** S2

Figure S1. Relative total energy profiles of **QST4-QST5**, **QST6-QST7** and **QST11-QST12** S3

Figure S2. Optimized geometries and some selected structural parameters of **QST6**, **QST8** and **QST11** S3

Figure S3. The frontier molecular orbitals of **QST1- QST14** S4

Figure S4. The MEP surfaces of **QST1- QST14** S5

Table S2. Intramolecular hydrogen bonding parameters of **QST1- QST14** S5-S6

Table S3. Selected NBO donor-acceptor interactions of **QST1- QST14** S7-S12

Table S4. Charges accumulated on all atoms with NBO analysis of **QST1- QST14** S12-S35

Table S5. The quantum chemical reactivity descriptors (eV) of **QST1-QST14** S35

Table S6. Cartesian coordinates for the optimized structures of **QST1- QST14** S36-S50

^{1}H NMR, ^{13}C NMR and IR spectra of quinoline-8-sulfonohydrazide and **QST1- QST14** S51-S65

^{15}N NMR spectrum of QST3 S66

Table S7. Antimicrobial activity (MIC values, μg/mL) of QST compounds on bacterial and *Candida* species. S67

Table S1. Total energies E_t (au) and relative total energies ΔE (kcal/mol) of **QST4-QST5**, **QST6-QST7** and **QST11-QST12** using B3LYP/6-31+G(d,p) basis set.

Compound	B3LYP/6-31+G(d, p)	
	E_t (au)	ΔE (kcal/mol)
**QST4**	-2243,5314582	0.028
**QST5**	-2243,531503	0.000
**QST6**	-1883,177642	0.000
**QST7**	-1883,177188	0.285

**QST11**	-1898,468691	0.000
**QST12**	-1898,465910	1.746

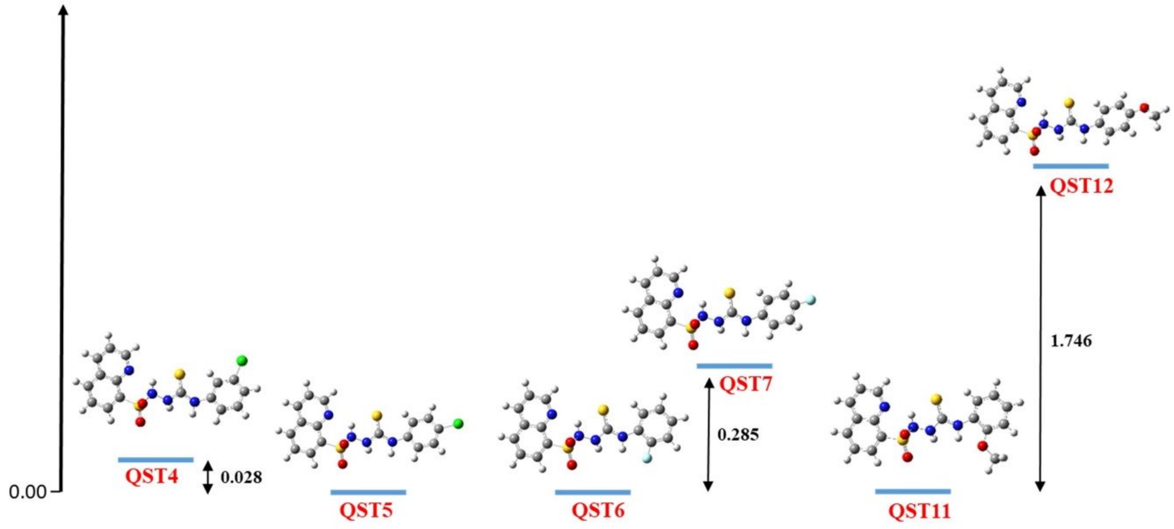

Figure S1. Relative total energy profiles of **QST4-QST5**, **QST6-QST7** and **QST11-QST12** at the B3LYP/6-31+G(d,p) basis set.

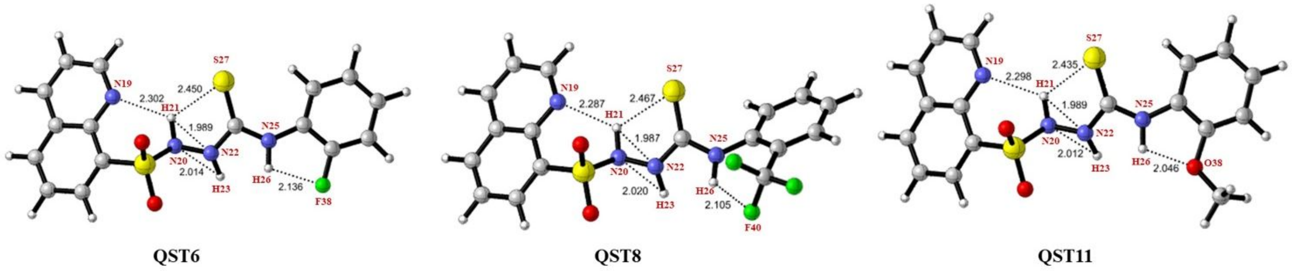

Figure S2. Optimized geometries and some selected structural parameters of **QST6**, **QST8** and **QST11** using B3LYP/6-31+G(d,p) basis set.

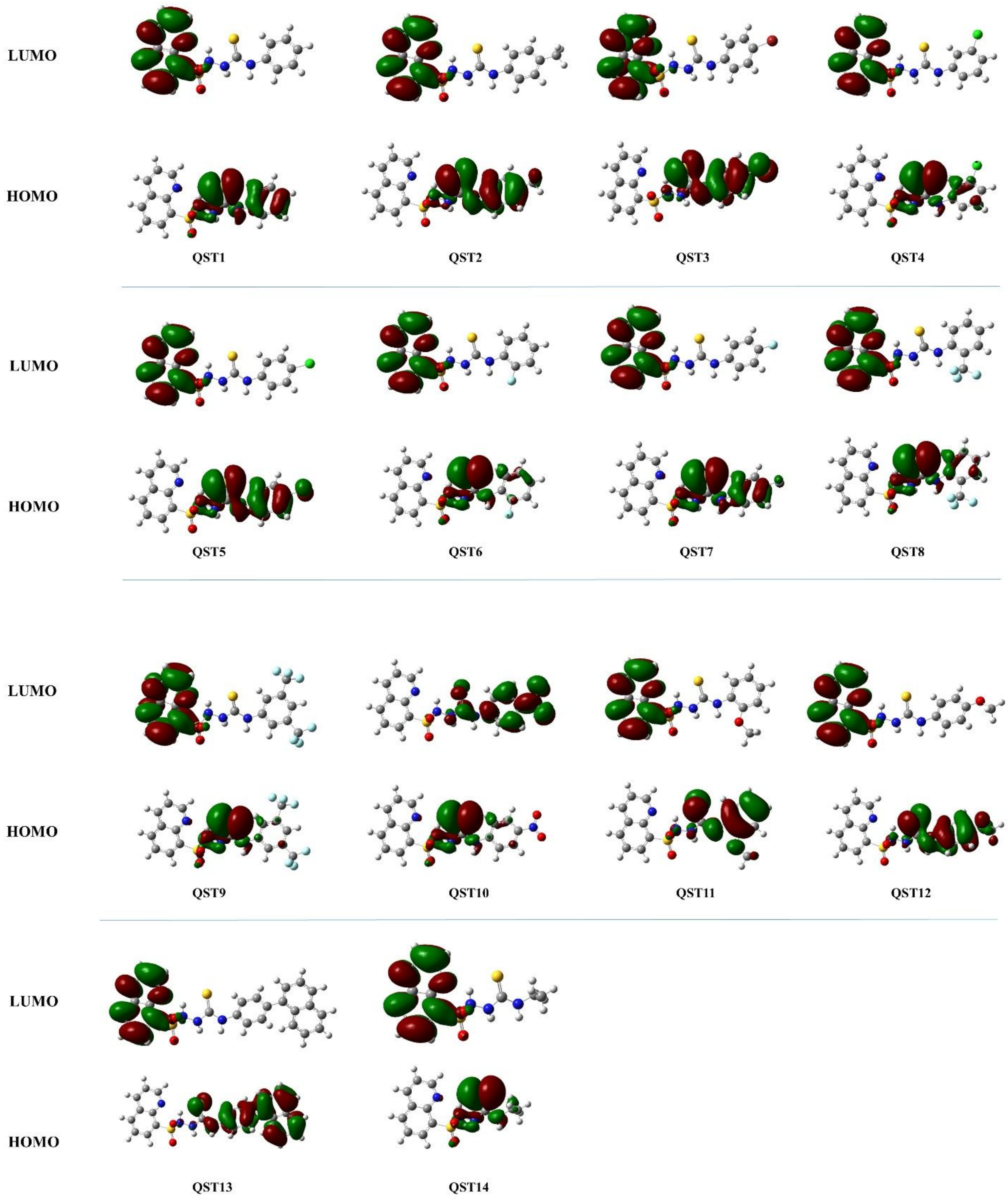

Figure S3. The frontier molecular orbitals of **QST1- QST14** using B3LYP/6-31+G(d,p) basis set

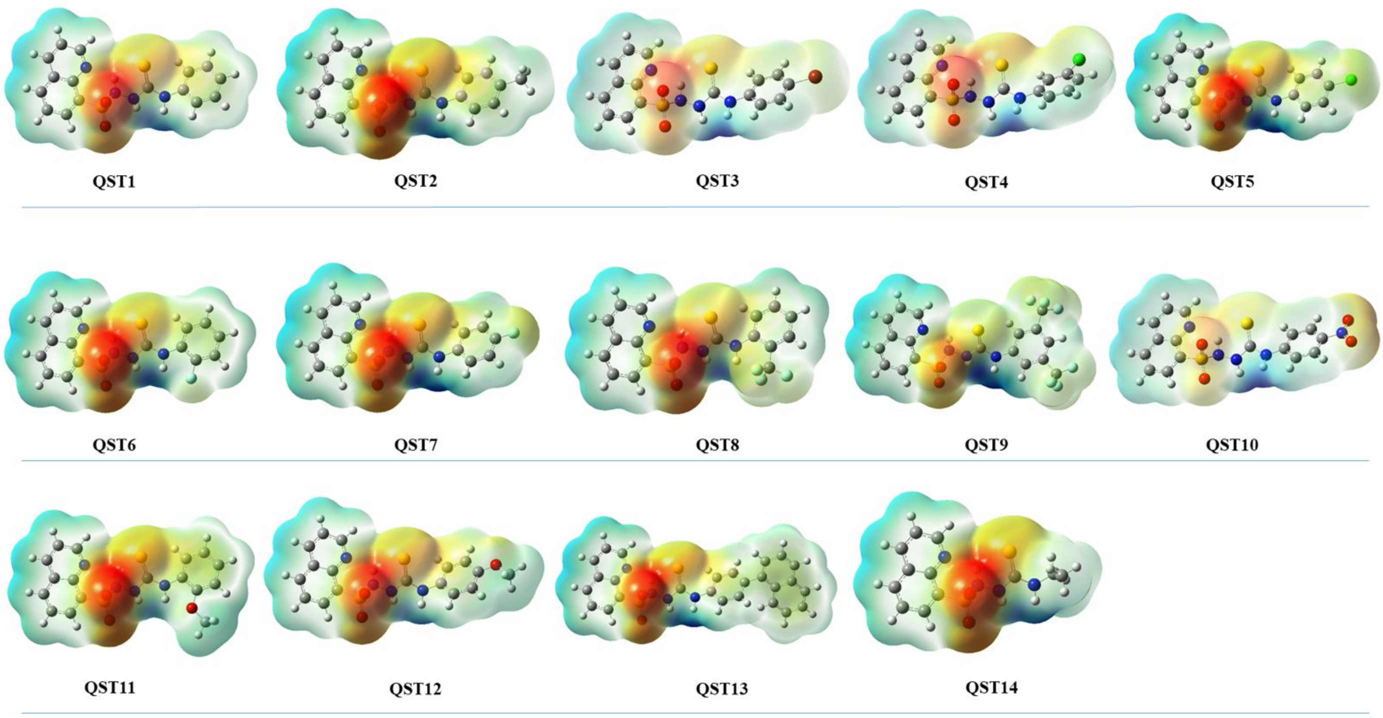

Figure S4. The MEP surfaces of **QST1- QST14** using B3LYP/6-31+G(d,p) basis set.

Table S2. Intramolecular hydrogen bonding parameters of **QST1- QST14** using B3LYP/6-31+G(d,p) basis set.

Compounds	D-H···A	D-H (Å)	H···A (Å)	D-H···A (°)
**QST1**	N20-H21···N19	1.033	2.289	118.20
	N22-H23···N20	1.012	2.014	39.79
	N20-H21···N22	1.033	1.989	4159
	N20-H21···S27	1.033	2.458	114.76
**QST 2**	N20-H21···N19	1.033	2.292	118.27
	N22-H23···N20	1.012	2.015	39.82
	N20-H21···N22	1.033	1.989	41.65
	N20-H21···S27	1.033	2.456	114.95
**QST 3**	N20-H21···N19	1.033	2.285	118.41
	N22-H23···N20	1.012	2.016	39.70
	N20-H21···N22	1.033	1.990	41.67
	N20-H21···S27	1.033	2.473	114.15
**QST 4**	N20-H21···N19	1.033	2.280	119.00
	N22-H23···N20	1.012	2.017	39.66
	N20-H21···N22	1.033	1.990	41.56
	N20-H21···S27	1.033	2.471	114.44
**QST 5**	N20-H21···N19	1.033	2.287	118.34

	N22-H23···N20	1.012	2.017	39.64
	N20-H21···N22	1.033	1.990	41.53
	N20-H21···S27	1.033	2.474	114.27
**QST 6**	N20-H21···N19	1.033	2.302	117.70
	N22-H23···N20	1.012	2.014	39.78
	N20-H21···N22	1.033	1.989	41.55
	N20-H21···S27	1.033	2.450	114.69
	N25-H26···F38	1.014	2.136	109.11
**QST 7**	N20-H21···N19	1.033	2.286	118.62
	N22-H23···N20	1.012	2.017	39.69
	N20-H21···N22	1.033	1.989	41.65
	N20-H21···S27	1.033	2.467	114.69
**QST 8**	N20-H21···N19	1.033	2.287	118.30
	N22-H23···N20	1.012	2.020	39.48
	N20-H21···N22	1.033	1.987	41.68
	N20-H21···S27	1.033	2.467	114.51
	N25-H26···F40	1.013	2.105	128.61
**QST 9**	N20-H21···N19	1.032	2.271	119.06
	N22-H23···N20	1.012	2.020	39.45
	N20-H21···N22	1.033	1.990	41.45
	N20-H21···S27	1.032	2.493	113.67
**QST 10**	N20-H21···N19	1.032	2.278	118.54
	N22-H23···N20	1.012	2.018	39.48
	N20-H21···N22	1.032	1.991	41.37
	N20-H21···S27	1.032	2.490	113.49
**QST 11**	N20-H21···N19	1.033	2.298	118.02
	N22-H23···N20	1.012	2.012	39.94
	N20-H21···N22	1.033	1.989	41.63
	N20-H21···S27	1.033	2.435	115.27
	N25-H26···O38	1.015	2.046	111.07
**QST 12**	N20-H21···N19	1.034	2.291	118.55
	N22-H23···N20	1.012	2.015	39.90
	N20-H21···N22	1.034	1.988	41.75
	N20-H21···S27	1.034	2.452	115.29
**QST 13**	N20-H21···N19	1.034	2.282	119.03
	N22-H23···N20	1.012	2.016	39.86
	N20-H21···N22	1.034	1.988	41.76
	N20-H21···S27	1.034	2.455	115.21
**QST 14**	N20-H21···N19	1.034	2.281	119.09
	N22-H23···N20	1.012	2.013	40.08
	N20-H21···N22	1.034	1.990	41.72
	N20-H21···S27	1.034	2.449	115.39

Table S3. Selected NBO donor-acceptor interactions for the hydrogen bondings and intramolecular interactions of **QST1-14** using B3LYP/6-31+G(d,p) basis set.

Donor NBO (i)	Acceptor NBO (j)	$E^{(2)a}$ kcal/mol	E(j)-E(i)[b] a.u.	F(i,j)[c] a.u.
**QST1**				
LP(2) O17	σ*C2S16	17.94	0.43	0.078
LP(2) O17	σ*S16O18	15.71	0.55	0.084
LP(3) O17	σ*S16N20	30.52	0.35	0.094
LP(2) O18	σ*S16O17	22.48	0.56	0.101
LP(3) O18	σ*C2S16	14.49	0.43	0.071
LP(3) O18	σ*S16N20	18.66	0.36	0.075
LP(1) N19	σ*C3C4	10.50	0.87	0.086
LP(1) N19	σ*C11C12	9.97	0.88	0.085
LP(1) N19	σ*N20H21	4.52	0.78	0.053
LP(1) N20	σ*S16O17	7.60	0.66	0.064
LP(1) N20	σ*N22H23	1.20	0.79	0.028
LP(1) N20	σ*N22C24	5.29	0.79	0.058
LP(1) N22	σ*N20H21	1.39	0.72	0.030
LP(1) N22	σ*C24S27	43.79	0.28	0.103
LP(1) N25	σ*C24S27	33.89	0.25	0.084
LP(1) N25	π*C28C29	24.68	0.30	0.078
LP(1) S27	σ*N20H21	0.98	1.12	0.030
LP(2) S27	σ*N20H21	6.64	0.61	0.058
LP(2) S27	σ*C24N25	12.49	0.62	0.081
**QST2**				
LP(2) O17	σ*C2S16	17.87	0.43	0.078
LP(2) O17	σ*S16O18	15.80	0.55	0.084
LP(3) O17	σ*S16N20	30.49	0.35	0.094
LP(2) O18	σ*S16O17	22.47	0.56	0.101
LP(3) O18	σ*C2S16	14.60	0.43	0.071
LP(3) O18	σ*S16N20	18.49	0.36	0.075
LP(1) N19	σ*C3C4	10.51	0.87	0.086
LP(1) N19	σ*C11C12	9.98	0.88	0.085
LP(1) N19	σ*N20H21	4.50	0.78	0.053
LP(1) N20	σ*S16O17	7.62	0.66	0.064
LP(1) N20	σ*N22H23	1.19	0.79	0.028
LP(1) N22	σ*N20H21	1.38	0.72	0.030
LP(1) N22	σ*C24S27	45.96	0.27	0.104
LP(1) N25	σ*C24S27	39.59	0.24	0.089
LP(1) N25	π*C28C29	22.40	0.30	0.075
LP(1) S27	σ*N20H21	0.98	1.12	0.030
LP(2) S27	σ*N20H21	6.69	0.61	0.059
LP(2) S27	σ*C24N25	12.46	0.63	0.081
**QST3**				
LP(2) O17	σ*C2S16	17.94	0.43	0.078
LP(2) O17	σ*S16O18	15.71	0.55	0.084
LP(3) O17	σ*S16N20	30.65	0.35	0.094

LP(2) O18	σ*S16O17	22.50	0.56	0.101
LP(3) O18	σ*C2S16	14.12	0.44	0.070
LP(3) O18	σ*S16N20	19.18	0.36	0.076
LP(1) N19	σ*C3C4	10.49	0.87	0.086
LP(1) N19	σ*C11C12	9.95	0.88	0.085
LP(1) N19	σ*N20H21	4.59	0.78	0.054
LP(1) N20	σ*S16O17	7.52	0.66	0.064
LP(1) N20	σ*N22H23	1.19	0.79	0.028
LP(1) N22	σ*N20H21	1.39	0.72	0.030
LP(1) N25	π*C28C29	27.29	0.29	0.082
LP(1) S27	σ*N20H21	0.93	1.12	0.029
LP(2) S27	σ*N20H21	6.25	0.62	0.057
LP(2) S27	σ*C24N25	12.43	0.62	0.080
**QST4**				
LP(2) O17	σ*C2S16	17.85	0.43	0.078
LP(2) O17	σ*S16O18	15.86	0.55	0.085
LP(3) O17	σ*S16N20	30.64	0.35	0.094
LP(2) O18	σ*S16O17	22.49	0.56	0.101
LP(3) O18	σ*C2S16	14.29	0.44	0.071
LP(3) O18	σ*S16N20	18.93	0.36	0.076
LP(1) N19	σ*C3C4	10.49	0.87	0.086
LP(1) N19	σ*C11C12	9.94	0.88	0.085
LP(1) N19	σ*N20H21	4.69	0.78	0.054
LP(1) N20	σ*S16O17	7.56	0.66	0.064
LP(1) N20	σ*N22H23	1.16	0.79	0.027
LP(1) N22	σ*N20H21	1.30	0.72	0.029
LP(1) N25	π*C28C29	24.12	0.30	0.077
LP(1) S27	σ*N20H21	0.93	1.12	0.029
LP(2) S27	σ*N20H21	6.28	0.62	0.057
LP(2) S27	σ*C24N25	12.66	0.62	0.081
**QST5**				
LP(2) O17	σ*C2S16	17.94	0.43	0.078
LP(2) O17	σ*S16O18	15.70	0.55	0.084
LP(3) O17	σ*S16N20	30.64	0.35	0.094
LP(2) O18	σ*S16O17	22.50	0.56	0.101
LP(3) O18	σ*C2S16	14.18	0.44	0.070
LP(3) O18	σ*S16N20	19.09	0.36	0.076
LP(1) N19	σ*C3C4	10.49	0.87	0.086
LP(1) N19	σ*C11C12	9.95	0.88	0.085
LP(1) N19	σ*N20H21	4.56	0.78	0.054
LP(1) N20	σ*S16O17	7.53	0.66	0.064
LP(1) N20	σ*N22H23	1.17	0.79	0.028
LP(1) N22	σ*N20H21	1.35	0.72	0.029
LP(1) N25	π*C28C29	24.32	0.30	0.077
LP(1) S27	σ*N20H21	0.92	1.12	0.029
LP(2) S27	σ*N20H21	6.23	0.62	0.057
LP(2) S27	σ*C24N25	12.54	0.62	0.081

**QST6**				
LP(2) O17	σ*C2S16	18.09	0.43	0.078
LP(2) O17	σ*S16O18	15.51	0.55	0.084
LP(3) O17	σ*S16N20	30.70	0.35	0.094
LP(2) O18	σ*S16O17	22.51	0.56	0.101
LP(3) O18	σ*C2S16	14.36	0.43	0.071
LP(3) O18	σ*S16N20	18.93	0.36	0.076
LP(1) N19	σ*C3C4	10.49	0.87	0.086
LP(1) N19	σ*C11C12	9.97	0.88	0.085
LP(1) N19	σ*N20H21	4.32	0.78	0.052
LP(1) N20	σ*S16O17	7.53	0.66	0.064
LP(1) N20	σ*N22H23	1.17	0.79	0.027
LP(1) N22	σ*N20H21	1.31	0.72	0.029
LP(1) N25	π*C28C29	34.00	0.30	0.091
LP(1) S27	σ*N20H21	1.07	1.12	0.031
LP(2) S27	σ*N20H21	6.79	0.62	0.059
LP(2) S27	σ*C24N25	12.04	0.63	0.079
LP(1) F38	σ*N25H26	0.61	1.45	0.027
LP(2) F38	σ*N25H26	2.07	0.84	0.037
**QST7**				
LP(2) O17	σ*C2S16	17.84	0.43	0.078
LP(2) O17	σ*S16O18	15.86	0.55	0.085
LP(3) O17	σ*S16N20	30.61	0.35	0.094
LP(2) O18	σ*S16O17	22.48	0.56	0.101
LP(3) O18	σ*C2S16	14.37	0.43	0.071
LP(3) O18	σ*S16N20	18.80	0.36	0.076
LP(1) N19	σ*C3C4	10.50	0.87	0.086
LP(1) N19	σ*C11C12	9.95	0.88	0.085
LP(1) N19	σ*N20H21	4.59	0.78	0.054
LP(1) N20	σ*S16O17	7.56	0.66	0.064
LP(1) N20	σ*N22H23	1.15	0.79	0.027
LP(1) N22	σ*N20H21	1.30	0.72	0.029
LP(1) N25	π*C28C29	19.85	0.30	0.070
LP(1) S27	σ*N20H21	0.94	1.12	0.029
LP(2) S27	σ*N20H21	6.38	0.61	0.073
LP(2) S27	σ*C24N25	12.55	0.62	0.081
LP(2) F38	σ*C31C34	6.36	0.97	0.070
LP(3) F38	π*C31C34	18.06	0.43	0.085
**QST8**				
LP(2) O17	σ*C2S16	18.07	0.43	0.078
LP(2) O17	σ*S16O18	15.61	0.55	0.084
LP(3) O17	σ*S16N20	30.89	0.35	0.094
LP(2) O18	σ*S16O17	22.50	0.56	0.101
LP(3) O18	σ*C2S16	14.42	0.43	0.071
LP(3) O18	σ*S16N20	18.91	0.36	0.076
LP(1) N19	σ*C3C4	10.48	0.87	0.086
LP(1) N19	σ*C11C12	9.95	0.88	0.085
LP(1) N19	σ*N20H21	4.57	0.78	0.054

LP(1) N20	σ*S16O17	7.50	0.66	0.064
LP(1) N20	σ*N22H23	1.06	0.79	0.026
LP(1) N22	σ*N20H21	1.10	0.72	0.027
LP(1) N25	π*C28C29	25.64	0.28	0.079
LP(1) S27	σ*N20H21	0.95	1.12	0.029
LP(2) S27	σ*N20H21	6.35	0.62	0.057
LP(2) S27	σ*C24N25	12.67	0.61	0.080
LP(1) F40	σ*N25H26	1.13	1.51	0.037
LP(2) F40	σ*N25H26	0.53	0.85	0.019
LP(3) F40	σ*N25H26	2.74	0.86	0.044
**QST9**				
LP(2) O17	σ*C2S16	17.99	0.43	0.078
LP(2) O17	σ*S16O18	15.55	0.55	0.084
LP(3) O17	σ*S16N20	30.90	0.35	0.094
LP(2) O18	σ*S16O17	22.55	0.56	0.101
LP(3) O18	σ*C2S16	13.66	0.44	0.069
LP(3) O18	σ*S16N20	19.85	0.36	0.077
LP(1) N19	σ*C3C4	10.48	0.87	0.086
LP(1) N19	σ*C11C12	9.87	0.88	0.084
LP(1) N19	σ*N20H21	4.83	0.78	0.055
LP(1) N20	σ*S16O17	7.42	0.66	0.064
LP(1) N20	σ*N22H23	1.12	0.79	0.027
LP(1) N22	σ*N20H21	1.20	0.72	0.028
LP(1) N25	π*C28C29	25.43	0.29	0.078
LP(1) S27	σ*N20H21	0.86	1.13	0.028
LP(2) S27	σ*N20H21	5.70	0.62	0.054
LP(2) S27	σ*C24N25	12.78	0.60	0.080
**QST10**				
LP(2) O17	σ*) C2S16	17.88	0.43	0.078
LP(2) O17	σ*S16O18	15.66	0.55	0.084
LP(3) O17	σ*S16N20	30.72	0.35	0.094
LP(2) O18	σ*S16O17	22.49	0.56	0.101
LP(3) O18	σ*C2S16	14.20	0.43	0.070
LP(3) O18	σ*S16N20	19.08	0.36	0.076
LP(1) N19	σ*C3C4	10.48	0.87	0.086
LP(1) N19	σ*C11C12	9.94	0.88	0.085
LP(1) N19	σ*N20H21	4.55	0.77	0.053
LP(1) N20	σ*S16O17	7.57	0.66	0.064
LP(1) N20	σ*N22H23	1.21	0.79	0.028
LP(1) N22	σ*N20H21	1.38	0.72	0.030
LP(1) N25	π*C28C29	24.48	0.29	0.077
LP(1) S27	σ*N20H21	0.99	1.12	0.030
LP(2) S27	σ*N20H21	6.56	0.62	0.058
LP(2) S27	σ*C24N25	12.57	0.62	0.081
**QST11**				
LP(2) O17	σ*C2S16	17.89	0.43	0.078
LP(2) O17	σ*S16O18	15.81	0.55	0.084

LP(3) O17	σ*) S16N20	30.51	0.35	0.094
LP(2) O18	σ*S16O17	22.47	0.56	0.101
LP(3) O18	σ*C2S16	14.63	0.43	0.071
LP(3) O18	σ*S16N20	18.50	0.36	0.075
LP(1) N19	σ*C3C4	10.51	0.87	0.086
LP(1) N19	σ*C11C12	9.98	0.88	0.085
LP(1) N19	σ*N20H21	4.49	0.78	0.053
LP(1) N20	σ*S16O17	7.62	0.66	0.064
LP(1) N20	σ*N22H23	1.19	0.79	0.028
LP(1) N22	σ*N20H21	1.38	0.72	0.030
LP(1) N22	σ*C24S27	41.54	0.29	0.102
LP(1) N25	σ*C24S27	31.55	0.49	0.061
LP(1) N25	π*C24S27	8.58	0.26	0.083
LP(1) N25	π*C28C29	22.51	0.29	0.074
LP(1) S27	σ*N20H21	0.99	1.12	0.030
LP(2) S27	σ*N20H21	6.68	0.61	0.059
LP(2) S27	σ*C24N25	12.41	0.63	0.081
LP(1) O38	σ*N25H26	0.88	1.02	0.027
LP(2) O38	π*C28C29	20.23	0.33	0.080
**QST12**				
LP(2) O17	σ*C2S16	17.87	0.43	0.078
LP(2) O17	σ*S16O18	15.78	0.55	0.084
LP(3) O17	σ*S16N20	30.53	0.35	0.094
LP(2) O18	σ*S16O17	22.47	0.56	0.101
LP(3) O18	σ*C2S16	14.53	0.43	0.071
LP(3) O18	σ*S16N20	18.58	0.36	0.075
LP(1) N19	σ*C3C4	10.50	0.87	0.086
LP(1) N19	σ*C11C12	9.97	0.88	0.085
LP(1) N19	σ*N20H21	4.51	0.78	0.053
LP(1) N20	σ*S16O17	7.61	0.66	0.064
LP(1) N20	σ*N22H23	1.20	0.79	0.028
LP(1) N22	σ*N20H21	1.38	0.72	0.030
LP(1) N25	π*C28C29	22.60	0.30	0.075
LP(1) S27	σ*N20H21	0.98	1.12	0.030
LP(2) S27	σ*N20H21	6.67	0.61	0.059
LP(2) S27	σ*C24N25	12.45	0.63	0.081
**QST13**				
LP(2) O17	σ*C2S16	17.69	0.43	0.078
LP(2) O17	σ*S16O18	16.08	0.55	0.085
LP(3) O17	σ*S16N20	30.54	0.35	0.094
LP(2) O18	σ*S16O17	22.45	0.56	0.101
LP(3) O18	σ*C2S16	14.74	0.43	0.072
LP(3) O18	σ*S16N20	18.29	0.36	0.074
LP(1) N19	σ*C3C4	10.51	0.87	0.086
LP(1) N19	σ*C11C12	9.97	0.88	0.085
LP(1) N19	σ*N20H21	4.68	0.78	0.054
LP(1) N20	σ*S16O17	7.64	0.66	0.065
LP(1) N20	σ*N22H23	1.16	0.79	0.027

LP(1) N22	σ*N20H21	1.30	0.72	0.029
LP(1) N22	σ*C24S27	46.45	0.27	0.104
LP(1) N25	σ*C24S27	43.30	0.23	0.093
LP(1) N25	π*C24S27	5.24	0.52	0.049
LP(1) N25	BD*(2) C28C30	14.54	0.31	0.060
LP(1) S27	σ*N20H21	0.97	1.11	0.030
LP(2) S27	σ*N20H21	6.70	0.61	0.059
LP(2) S27	σ*C24N25	12.68	0.63	0.081
**QST14**				
LP(2) O17	σ*C2S16	17.88	0.43	0.078
LP(2) O17	σ*S16O18	15.79	0.55	0.084
LP(3) O17	σ*S16N20	30.30	0.35	0.094
LP(2) O18	σ*S16O17	22.47	0.56	0.101
LP(3) O18	σ*C2S16	14.82	0.43	0.072
LP(3) O18	σ*S16N20	18.16	0.36	0.074
LP(1) N19	σ*C3C4	10.51	0.87	0.086
LP(1) N19	σ*C11C12	9.99	0.88	0.085
LP(1) N19	σ*N20H21	4.48	0.78	0.053
LP(1) N20	σ*S16O17	7.65	0.65	0.065
LP(1) N20	σ*N22H23	1.17	0.79	0.028
LP(1) N22	σ*S16N20	8.06	0.37	0.049
LP(1) N22	σ*N20H21	1.41	0.72	0.030
LP(1) N25	σ*C28C31	5.46	0.63	0.056
LP(1) S27	σ*N20H21	0.97	1.12	0.030
LP(2) S27	σ*N20H21	6.74	0.61	0.059
LP(2) S27	σ*C24N25	12.02	0.63	0.080

[a] $E^{(2)}$ means energy of hyperconjugative interactions (stabilization energy).
[b] Energy difference between donor and acceptor i and j NBO orbitals.
[c] F(i, j) is the Fork matrix element between i and j NBO orbitals.

Table S4. Charges accumulated on all atoms by NBO analysis of **QST1- QST14** using B3LYP/6-31+G (d, p).

QST1

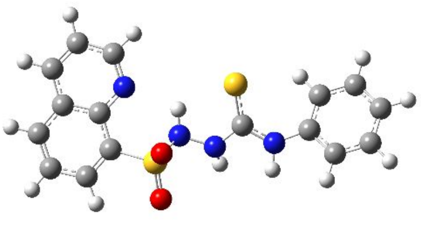

Atom No	Natural Charge
C 1	-0.20075
C 2	-0.29137

C	3	0.17529
C	4	-0.09414
C	5	-0.18966
C	6	-0.23741
H	7	0.28323
C	8	-0.16775
H	9	0.25006
H	10	0.25833
C	11	-0.28165
C	12	0.05212
H	13	0.25158
H	14	0.25834
H	15	0.24467
S	16	2.26401
O	17	-0.89767
O	18	-0.95144
N	19	-0.45780
N	20	-0.71171
H	21	0.44967
N	22	-0.49842
H	23	0.44089
C	24	0.23954
N	25	-0.63040
H	26	0.43287
S	27	-0.16571
C	28	0.14905
C	29	-0.26372
C	30	-0.24482
C	31	-0.23311
H	32	0.24223
C	33	-0.23063
H	34	0.26542
C	35	-0.25517
H	36	0.24888

H	37	0.24932
H	38	0.24779

QST2

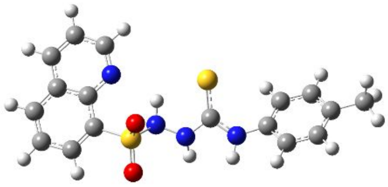

Atom No		Natural Charge
C	1	-0.20094
C	2	-0.29108
C	3	0.17520
C	4	-0.09420
C	5	-0.18993
C	6	-0.23750
H	7	0.28323
C	8	-0.16799
H	9	0.24990
H	10	0.25817
C	11	-0.28177
C	12	0.05202
H	13	0.25142
H	14	0.25817
H	15	0.24470
S	16	2.26398
O	17	-0.89816
O	18	-0.95172
N	19	-0.45724
N	20	-0.71174
H	21	0.44942
N	22	-0.50001

H	23	0.44058
C	24	0.24028
N	25	-0.63235
H	26	0.43296
S	27	-0.17013
C	28	0.13950
C	29	-0.25446
C	30	-0.23354
C	31	-0.22922
H	32	0.24279
C	33	-0.22643
H	34	0.26372
C	35	-0.04833
H	36	0.24369
H	37	0.24413
C	38	-0.70822
H	39	0.25509
H	40	0.24635
H	41	0.24967

QST3

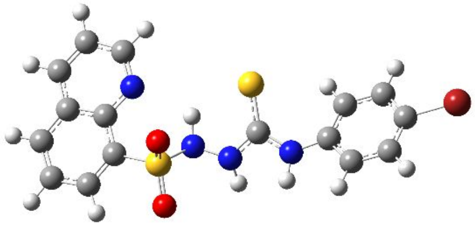

Atom	No	Natural Charge
C	1	-0.19998
C	2	-0.29243

C	3	0.17550
C	4	-0.09402
C	5	-0.18875
C	6	-0.23729
H	7	0.28337
C	8	-0.16709
H	9	0.25055
H	10	0.25883
C	11	-0.28134
C	12	0.05248
H	13	0.25205
H	14	0.25883
H	15	0.24469
S	16	2.26315
O	17	-0.89696
O	18	-0.95108
N	19	-0.45929
N	20	-0.71017
H	21	0.45039
N	22	-0.49657
H	23	0.44158
C	24	0.23853
N	25	-0.62636
H	26	0.43279
S	27	-0.16205
C	28	0.15120
C	29	-0.25145
C	30	-0.23480
C	31	-0.24240
H	32	0.24656

C	33	-0.23927
H	34	0.27234
C	35	-0.13696
H	36	0.26435
H	37	0.26482
Br	38	0.06624

QST4

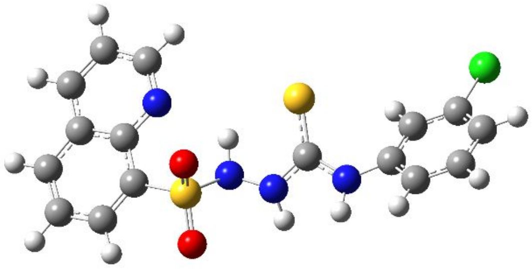

Atom	No	Natural Charge
C	1	-0.19983
C	2	-0.29292
C	3	0.17559
C	4	-0.09408
C	5	-0.18860
C	6	-0.23747
H	7	0.28337
C	8	-0.16723
H	9	0.25052
H	10	0.25875
C	11	-0.28115
C	12	0.05297
H	13	0.25202
H	14	0.25893
H	15	0.24524

S	16	2.26367
O	17	-0.89742
O	18	-0.95132
N	19	-0.45926
N	20	-0.71018
H	21	0.45037
N	22	-0.49628
H	23	0.44267
C	24	0.23611
N	25	-0.63623
H	26	0.43525
S	27	-0.15581
C	28	0.16445
C	29	-0.26708
C	30	-0.25479
C	31	-0.21970
H	32	0.24685
C	33	-0.04247
H	34	0.27837
C	35	-0.26511
H	36	0.25423
H	37	0.26250
Cl	38	0.00505

QST5

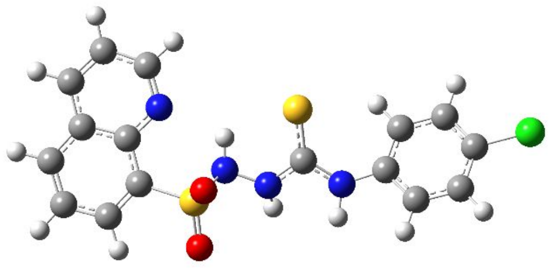

Atom	No	Natural Charge
C	1	-0.20002
C	2	-0.29236
C	3	0.17547
C	4	-0.09404
C	5	-0.18879
C	6	-0.23733
H	7	0.28335
C	8	-0.16717
H	9	0.25051
H	10	0.25880
C	11	-0.28135
C	12	0.05247
H	13	0.25201
H	14	0.25880
H	15	0.24478
S	16	2.26304
O	17	-0.89703
O	18	-0.95126
N	19	-0.45912
N	20	-0.70995
H	21	0.45042
N	22	-0.49626
H	23	0.44197
C	24	0.23807
N	25	-0.63335
H	26	0.43380

S	27	-0.16271
C	28	0.14779
C	29	-0.24935
C	30	-0.22950
C	31	-0.24322
H	32	0.24797
C	33	-0.24104
H	34	0.26998
C	35	-0.06454
H	36	0.26364
H	37	0.26408
Cl	38	0.00142

QST6

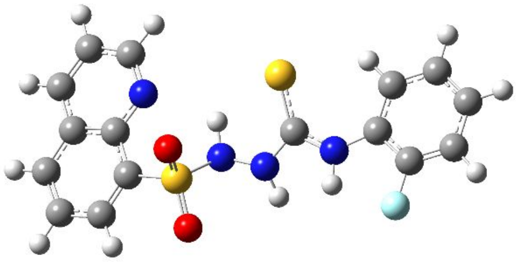

Atom	No	Natural Charge
C	1	-0.20026
C	2	-0.29195
C	3	0.17529
C	4	-0.09410
C	5	-0.18933
C	6	-0.23721
H	7	0.28357
C	8	-0.16744
H	9	0.25026
H	10	0.25863

C	11	-0.28170
C	12	0.05190
H	13	0.25179
H	14	0.25847
H	15	0.24419
S	16	2.26392
O	17	-0.89614
O	18	-0.94994
N	19	-0.45819
N	20	-0.71086
H	21	0.44995
N	22	-0.49253
H	23	0.44202
C	24	0.24264
N	25	-0.61912
H	26	0.44718
S	27	-0.16881
C	28	0.09866
C	29	0.38033
C	30	-0.24768
C	31	-0.29386
C	32	-0.23965
H	33	0.27586
C	34	-0.24632
H	35	0.26366
H	36	0.25286
H	37	0.25170
F	38	-0.35782

QST7

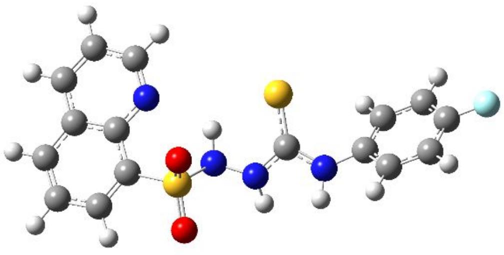

Atom	No	Natural Charge
C	1	-0.20011
C	2	-0.29233
C	3	0.17542
C	4	-0.09413
C	5	-0.18898
C	6	-0.23745
H	7	0.28335
C	8	-0.16742
H	9	0.25037
H	10	0.25864
C	11	-0.28141
C	12	0.05250
H	13	0.25186
H	14	0.25864
H	15	0.24492
S	16	2.26346
O	17	-0.89764
O	18	-0.95158
N	19	-0.45855
N	20	-0.71039
H	21	0.45012
N	22	-0.49768
H	23	0.44174
C	24	0.23928

N	25	-0.64022
H	26	0.43434
S	27	-0.16779
C	28	0.13335
C	29	-0.24346
C	30	-0.22039
C	31	-0.29380
C	32	-0.29184
H	33	0.26718
C	34	0.40192
H	35	0.26365
H	36	0.26405
H	37	0.24968
F	38	-0.34930

QST8

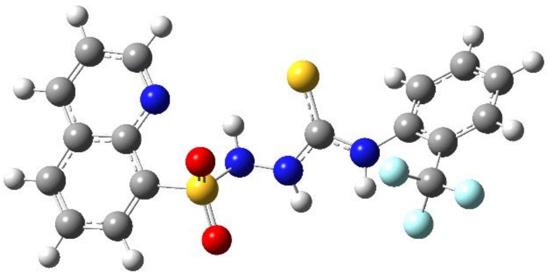

Atom	No	Natural Charge
C	1	-0.19972
C	2	-0.29260
C	3	0.17556
C	4	-0.09407
C	5	-0.18893
C	6	-0.23710
H	7	0.28371

C	8	-0.16723
H	9	0.25043
H	10	0.25887
C	11	-0.28157
C	12	0.05233
H	13	0.25192
H	14	0.25859
H	15	0.24456
S	16	2.26317
O	17	-0.89416
O	18	-0.94950
N	19	-0.45909
N	20	-0.70899
H	21	0.45096
N	22	-0.48700
H	23	0.44434
C	24	0.24117
N	25	-0.64799
H	26	0.44674
S	27	-0.16457
C	28	0.18839
C	29	-0.18662
C	30	-0.23546
C	31	-0.20560
C	32	-0.21354
H	33	0.26897
C	34	-0.25038
H	35	0.26721
H	36	0.25400
H	37	0.25379

C	38	1.09139
F	39	-0.35221
F	40	-0.37655
F	41	-0.35322

QST9

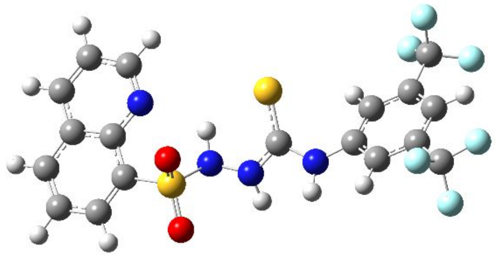

Atom	No	Natural Charge
C	1	-0.19841
C	2	-0.29496
C	3	0.17674
C	4	-0.09297
C	5	-0.18687
C	6	-0.23714
H	7	0.28366
C	8	-0.16596
H	9	0.25148
H	10	0.25976
C	11	-0.28055
C	12	0.05367
H	13	0.25293
H	14	0.25986
H	15	0.24536
S	16	2.26263
O	17	-0.89575
O	18	-0.95012

N	19	-0.46308
N	20	-0.70772
H	21	0.45183
N	22	-0.48854
H	23	0.44515
C	24	0.23259
N	25	-0.64020
H	26	0.43769
S	27	-0.14503
C	28	0.16580
C	29	-0.21759
C	30	-0.19813
C	31	-0.14258
C	32	-0.14214
H	33	0.28561
C	34	-0.19977
H	35	0.28204
H	36	0.26229
C	37	1.09574
C	38	1.09625
F	39	-0.36186
F	40	-0.35624
F	41	-0.35901
F	42	-0.36088
F	43	-0.35769
F	44	-0.35789

QST10

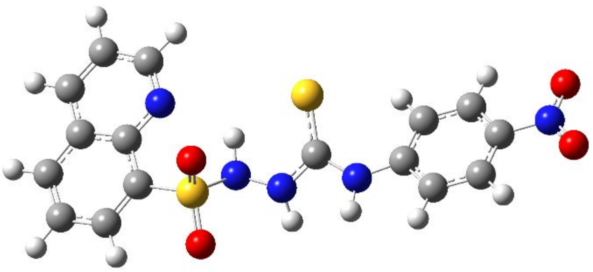

Atom	No	Natural Charge
C	1	-0.19958
C	2	-0.29285
C	3	0.17539
C	4	-0.09390
C	5	-0.18788
C	6	-0.23714
H	7	0.28361
C	8	-0.16656
H	9	0.25112
H	10	0.25940
C	11	-0.28100
C	12	0.05268
H	13	0.25260
H	14	0.25937
H	15	0.24460
S	16	2.26408
O	17	-0.89627
O	18	-0.94993
N	19	-0.46083
N	20	-0.71048
H	21	0.45004
N	22	-0.49482
H	23	0.44229
C	24	0.23670
N	25	-0.63527
H	26	0.43711

S	27	-0.14818
C	28	0.18519
C	29	-0.26114
C	30	-0.24219
C	31	-0.20037
C	32	-0.20168
H	33	0.27406
C	34	0.03970
H	35	0.25228
H	36	0.27573
H	37	0.27645
N	38	0.52044
O	39	-0.38544
O	40	-0.38733

QST11

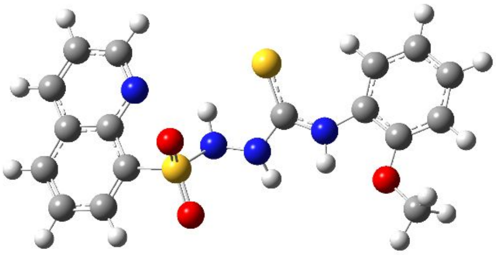

Atom	No	Natural Charge
C	1	-0.20112
C	2	-0.29079
C	3	0.17518
C	4	-0.09426
C	5	-0.19038
C	6	-0.23749
H	7	0.28327
C	8	-0.16824

H	9	0.24964
H	10	0.25792
C	11	-0.28199
C	12	0.05183
H	13	0.25119
H	14	0.25795
H	15	0.24462
S	16	2.26407
O	17	-0.89750
O	18	-0.95096
N	19	-0.45670
N	20	-0.71196
H	21	0.44924
N	22	-0.49792
H	23	0.44100
C	24	0.24234
N	25	-0.63144
H	26	0.44563
S	27	-0.17478
C	28	0.11448
C	29	0.28235
C	30	-0.22963
C	31	-0.30755
C	32	-0.24920
H	33	0.26795
C	34	-0.23188
H	35	0.24657
H	36	0.24571
H	37	0.24797
O	38	-0.54465

C	39	-0.33007
H	40	0.21036
H	41	0.23692
H	42	0.21229

QST12

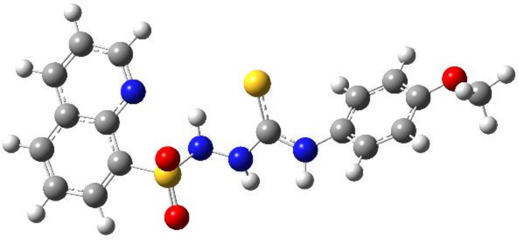

Atom	No	Natural Charge
C	1	-0.20080
C	2	-0.29136
C	3	0.17523
C	4	-0.09416
C	5	-0.18960
C	6	-0.23750
H	7	0.28323
C	8	-0.16779
H	9	0.25008
H	10	0.25832
C	11	-0.28158
C	12	0.05220
H	13	0.25159
H	14	0.25841
H	15	0.24481
S	16	2.26401
O	17	-0.89770
O	18	-0.95165

N	19	-0.45781
N	20	-0.71169
H	21	0.44955
N	22	-0.49945
H	23	0.44059
C	24	0.23934
N	25	-0.63359
H	26	0.43304
S	27	-0.16606
C	28	0.14217
C	29	-0.25351
C	30	-0.23385
C	31	-0.26554
C	32	-0.25980
H	33	0.26674
C	34	0.28542
H	35	0.25035
H	36	0.25625
H	37	0.24391
O	38	-0.57226
C	39	-0.31582
H	40	0.22743
H	41	0.20193
H	42	0.20692

QST13

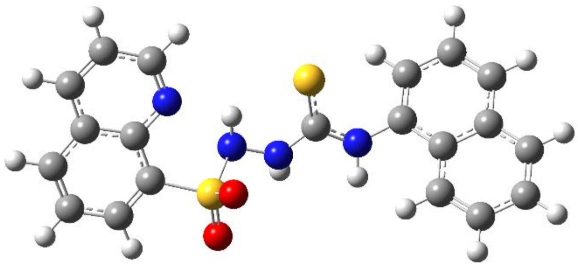

Natural

Atom	No	Charge
C	1	-0.20058
C	2	-0.29166
C	3	0.17534
C	4	-0.09425
C	5	-0.18963
C	6	-0.23761
H	7	0.28327
C	8	-0.16799
H	9	0.24997
H	10	0.25823
C	11	-0.28164
C	12	0.05247
H	13	0.25148
H	14	0.25827
H	15	0.24508
S	16	2.26381
O	17	-0.89853
O	18	-0.95203
N	19	-0.45736
N	20	-0.71119
H	21	0.44941
N	22	-0.50070
H	23	0.44015
C	24	0.24105
N	25	-0.64353
H	26	0.43300
S	27	-0.17089
C	28	0.16555
C	29	-0.06799

C	30	-0.22584
C	31	-0.22681
C	32	-0.05658
C	33	-0.23685
H	34	0.26110
C	35	-0.23424
H	36	0.24463
C	37	-0.20770
C	38	-0.21357
H	39	0.25046
C	40	-0.24419
H	41	0.25006
H	42	0.24446
H	43	0.24461
H	44	0.24896

QST14

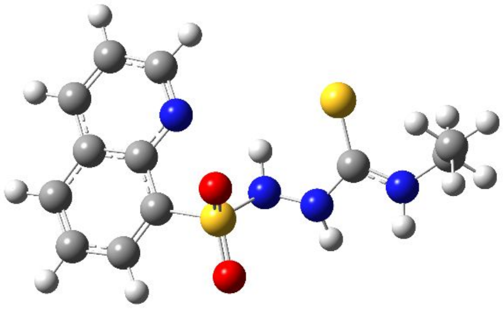

Atom	No	Natural Charge
C	1	-0.20138
C	2	-0.29061
C	3	0.17510

C	4	-0.09428
C	5	-0.19060
C	6	-0.23750
H	7	0.28305
C	8	-0.16831
H	9	0.24961
H	10	0.25783
C	11	-0.28207
C	12	0.05169
H	13	0.25117
H	14	0.25789
H	15	0.24449
S	16	2.26380
O	17	-0.90028
O	18	-0.95240
N	19	-0.45653
N	20	-0.71284
H	21	0.44928
N	22	-0.49980
H	23	0.44072
C	24	0.23758
N	25	-0.66542
H	26	0.43519
S	27	-0.18503
C	28	-0.27638
H	29	0.24069
H	30	0.24485
C	31	-0.70583
H	32	0.22891
H	33	0.25589

H 34 0.25149

Table S5. The quantum chemical reactivity descriptors (eV) of **QST1-QST14** using B3LYP/6-31+G(d,p) basis set.

	E_{HOMO}	E_{LUMO}	ΔE	I	A	η	σ	χ	μ	ω
**QST1**	-6,1075	-2,4514	3,6561	6,1075	2,4514	1,8280	0,5470	4,2795	-4,2795	5,0092
**QST2**	-6,0057	-2,4267	3,5791	6,0057	2,4267	1,7895	0,5588	4,2162	-4,2162	4,9668
**QST3**	-6,2142	-2,5230	3,6912	6,2142	2,5230	1,8456	0,5418	4,3686	-4,3686	5,1703
**QST4**	-6,2392	-2,5086	3,7306	6,2392	2,5086	1,8653	0,5361	4,3739	-4,3739	5,1281
**QST5**	-6,2237	-2,5178	3,7059	6,2237	2,5178	1,8529	0,5397	4,3708	-4,3708	5,1550
**QST6**	-6,2678	-2,4776	3,7902	6,2678	2,4776	1,8951	0,5277	4,3727	-4,3727	5,0446
**QST7**	-6,1802	-2,4963	3,6838	6,1802	2,4963	1,8419	0,5429	4,3383	-4,3383	5,1089
**QST8**	-6,2449	-2,4982	3,7467	6,2449	2,4982	1,8733	0,5338	4,3716	-4,3716	5,1007
**QST9**	-6,5448	-2,6427	3,9021	6,5448	2,6427	1,9510	0,5125	4,5938	-4,5938	5,4081
**QST10**	-6,5815	-2,7992	3,7823	6,5815	2,7992	1,8912	0,5288	4,6904	-4,6904	5,8164
**QST11**	-5,8713	-2,3861	3,4852	5,8713	2,3861	1,7426	0,5739	4,1287	-4,1287	4,8911
**QST12**	-5,8419	-2,4030	3,4389	5,8419	2,4030	1,7195	0,5816	4,1225	-4,1225	4,9419
**QST13**	-5,8242	-2,4661	3,3581	5,8242	2,4661	1,6791	0,5956	4,1452	-4,1452	5,1167
**QST14**	-6,0226	-2,3905	3,6321	6,0226	2,3905	1,8161	0,5506	4,2066	-4,2066	4,8718

Table S6. Cartesian coordinates for the optimized structures of **QST1-QST14**

QST1

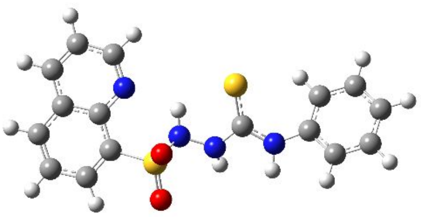

Center Number	Atomic Number	Coordinates (Angstroms) X	Y	Z
1	6	-3.860666	-1.952515	-0.311226
2	6	-2.968881	-0.943556	-0.022372
3	6	-3.379541	0.419871	0.032111
4	6	-4.761386	0.714795	-0.195253
5	6	-5.666106	-0.343349	-0.480030

6	6	-5.224282	-1.646291	-0.543932
7	1	-3.508373	-2.977562	-0.354526
8	6	-5.152589	2.077392	-0.127949
9	1	-6.713013	-0.106857	-0.651098
10	1	-5.917705	-2.450358	-0.768516
11	6	-4.208162	3.041376	0.140622
12	6	-2.860804	2.641788	0.327443
13	1	-6.194051	2.341844	-0.291580
14	1	-4.472878	4.091848	0.201263
15	1	-2.092470	3.386962	0.522671
16	16	-1.287629	-1.422024	0.425851
17	8	-1.042162	-1.094178	1.833489
18	8	-1.070624	-2.801272	-0.036432
19	7	-2.454225	1.388095	0.273993
20	7	-0.376652	-0.425387	-0.694873
21	1	-0.420260	0.551525	-0.361458
22	7	0.957632	-0.836980	-0.723033
23	1	1.148218	-1.485868	-1.476018
24	6	1.987653	-0.037916	-0.259484
25	7	3.187782	-0.694037	-0.379308
26	1	3.118972	-1.704783	-0.403723
27	16	1.735108	1.492507	0.352854
28	6	4.509140	-0.205858	-0.223195
29	6	5.468623	-1.092665	0.286891
30	6	4.895732	1.081948	-0.617723
31	6	6.801201	-0.697168	0.404137
32	1	5.168347	-2.089824	0.600083
33	6	6.229489	1.470259	-0.485030
34	1	4.161629	1.770546	-1.014260
35	6	7.188601	0.589773	0.023414
36	1	7.531829	-1.394801	0.802638
37	1	6.518775	2.471782	-0.789787
38	1	8.223718	0.902281	0.120575

QST2

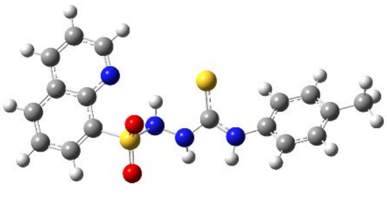

Center Number	Atomic Number	Coordinates (Angstroms) X	Y	Z
1	6	-4.286055	-1.880259	-0.296518
2	6	-3.359884	-0.900589	-0.015217
3	6	-3.726847	0.474892	0.046084
4	6	-5.101065	0.813734	-0.165608
5	6	-6.041705	-0.214785	-0.442481
6	6	-5.641775	-1.530820	-0.513959
7	1	-3.966455	-2.915731	-0.345154

8	6	-5.448107	2.187908	-0.091511
9	1	-7.082476	0.054842	-0.601542
10	1	-6.362548	-2.312152	-0.732634
11	6	-4.470699	3.120995	0.168149
12	6	-3.134678	2.678582	0.339455
13	1	-6.482413	2.485691	-0.242978
14	1	-4.701350	4.179199	0.233404
15	1	-2.340758	3.398424	0.527291
16	16	-1.690214	-1.433381	0.415735
17	8	-1.420742	-1.115067	1.821280
18	8	-1.523953	-2.819022	-0.048689
19	7	-2.768792	1.412769	0.279493
20	7	-0.758058	-0.466809	-0.713439
21	1	-0.768078	0.511466	-0.380703
22	7	0.563182	-0.919734	-0.748191
23	1	0.729435	-1.573633	-1.502569
24	6	1.619834	-0.147579	-0.297562
25	7	2.799988	-0.835683	-0.421501
26	1	2.707557	-1.844673	-0.438699
27	16	1.418779	1.395638	0.303100
28	6	4.130756	-0.370832	-0.256225
29	6	5.059044	-1.233601	0.337683
30	6	4.560231	0.875852	-0.728611
31	6	6.396550	-0.854190	0.460504
32	1	4.735361	-2.202293	0.710922
33	6	5.896484	1.244667	-0.586054
34	1	3.855397	1.550031	-1.197729
35	6	6.841128	0.394935	0.010178
36	1	7.099917	-1.541245	0.923869
37	1	6.211973	2.216839	-0.957046
38	6	8.279729	0.825705	0.177317
39	1	8.392864	1.506065	1.030776
40	1	8.935640	-0.032404	0.351945
41	1	8.644557	1.354686	-0.709506

QST3

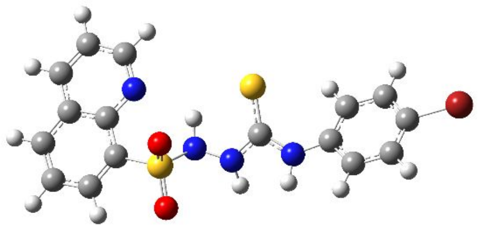

Center Number	Atomic Number	Coordinates (Angstroms) X	Y	Z
1	6	-5.356237	-1.838188	-0.243023
2	6	-4.415103	-0.863625	0.004656
3	6	-4.763490	0.517662	0.036413
4	6	-6.135493	0.868725	-0.168796
5	6	-7.091946	-0.154121	-0.410309
6	6	-6.709645	-1.476609	-0.454301

7	1	-5.050624	-2.878676	-0.269531
8	6	-6.464632	2.248586	-0.124224
9	1	-8.130954	0.124904	-0.564352
10	1	-7.442630	-2.253574	-0.646091
11	6	-5.472869	3.175155	0.102160
12	6	-4.140714	2.720103	0.269558
13	1	-7.496729	2.555764	-0.271680
14	1	-5.689500	4.237448	0.144323
15	1	-3.336117	3.434381	0.431290
16	16	-2.749561	-1.406598	0.434388
17	8	-2.463293	-1.061124	1.829906
18	8	-2.599028	-2.802507	-0.003330
19	7	-3.791036	1.448562	0.236330
20	7	-1.811356	-0.474057	-0.721061
21	1	-1.810209	0.510540	-0.409545
22	7	-0.498467	-0.946110	-0.754794
23	1	-0.346898	-1.627367	-1.487693
24	6	0.573355	-0.203008	-0.298372
25	7	1.732987	-0.932767	-0.402878
26	1	1.598903	-1.936939	-0.425577
27	16	0.408151	1.346442	0.294369
28	6	3.081816	-0.537459	-0.256366
29	6	3.994944	-1.522026	0.149405
30	6	3.549089	0.745627	-0.571493
31	6	5.355058	-1.236527	0.244566
32	1	3.640966	-2.519022	0.399985
33	6	4.909304	1.034737	-0.468369
34	1	2.857532	1.514964	-0.886096
35	6	5.805044	0.045463	-0.065285
36	1	6.051652	-2.003461	0.563287
37	1	5.266102	2.029970	-0.708830
38	35	7.660396	0.453321	0.082208

QST4

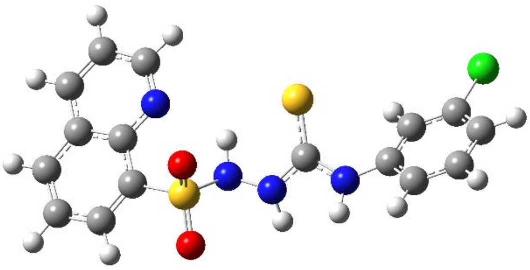

Center Number	Atomic Number	Coordinates (Angstroms) X	Y	Z
1	6	-4.587211	-1.690511	-0.530270
2	6	-3.601339	-0.816600	-0.128891
3	6	-3.865193	0.572025	0.052386
4	6	-5.201194	1.035704	-0.165527
5	6	-6.205565	0.114232	-0.567484
6	6	-5.903495	-1.216718	-0.753569
7	1	-4.345277	-2.738823	-0.668771
8	6	-5.446450	2.419870	0.030978

9	1	-7.216623	0.478142	-0.730312
10	1	-6.672511	-1.914973	-1.067864
11	6	-4.411404	3.245073	0.406757
12	6	-3.119162	2.685527	0.571312
13	1	-6.449431	2.810479	-0.119960
14	1	-4.563870	4.307179	0.567548
15	1	-2.280250	3.320465	0.848412
16	16	-1.993839	-1.514701	0.297287
17	8	-1.746360	-1.347592	1.732479
18	8	-1.911192	-2.860050	-0.291261
19	7	-2.847752	1.405990	0.400502
20	7	-0.951795	-0.523571	-0.709378
21	1	-0.911879	0.421021	-0.293183
22	7	0.335192	-1.065569	-0.734452
23	1	0.483327	-1.687716	-1.518928
24	6	1.421972	-0.422378	-0.177432
25	7	2.551935	-1.199096	-0.306239
26	1	2.379800	-2.195200	-0.375962
27	16	1.319493	1.077334	0.540526
28	6	3.902478	-0.855752	-0.061354
29	6	4.737236	-1.833899	0.496788
30	6	4.429960	0.390514	-0.424609
31	6	6.091460	-1.565917	0.693898
32	1	4.324133	-2.796426	0.785729
33	6	5.783241	0.634173	-0.204523
34	1	3.800159	1.152114	-0.862737
35	6	6.630962	-0.325117	0.351431
36	1	6.730807	-2.327343	1.129806
37	1	7.680163	-0.104542	0.510410
38	17	6.443563	2.199353	-0.660666

QST5

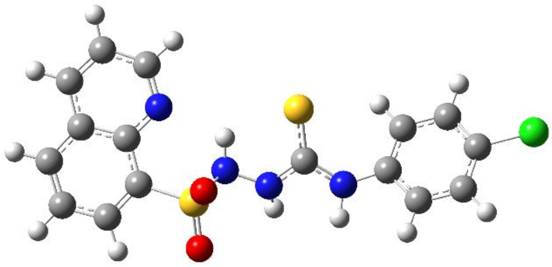

Center Number	Atomic Number	Coordinates (Angstroms) X	Y	Z
1	6	-4.640757	-1.861663	-0.262735
2	6	-3.706742	-0.883220	-0.003656
3	6	-4.065646	0.494816	0.046739
4	6	-5.440654	0.837951	-0.151824
5	6	-6.389572	-0.188954	-0.405514
6	6	-5.997221	-1.507772	-0.467411
7	1	-4.327153	-2.899306	-0.303500
8	6	-5.780277	2.214537	-0.088732
9	1	-7.430907	0.084089	-0.554503

10	1	-6.724493	-2.287772	-0.668409
11	6	-4.795331	3.145632	0.148560
12	6	-3.459475	2.698709	0.308268
13	1	-6.814902	2.515710	-0.230778
14	1	-5.020087	4.205575	0.205000
15	1	-2.660155	3.416922	0.478492
16	16	-2.035923	-1.418883	0.414907
17	8	-1.748665	-1.089674	1.814168
18	8	-1.875917	-2.807857	-0.041338
19	7	-3.100106	1.430445	0.257758
20	7	-1.108067	-0.464544	-0.731089
21	1	-1.112410	0.515896	-0.406547
22	7	0.208866	-0.925490	-0.774006
23	1	0.363688	-1.602676	-1.509903
24	6	1.275145	-0.177290	-0.315734
25	7	2.444852	-0.890776	-0.436582
26	1	2.331477	-1.897688	-0.445890
27	16	1.103602	1.366077	0.290446
28	6	3.781811	-0.455409	-0.273191
29	6	4.699098	-1.358807	0.281054
30	6	4.228468	0.801704	-0.701659
31	6	6.045551	-1.019801	0.408776
32	1	4.360031	-2.332644	0.624942
33	6	5.571585	1.149134	-0.563487
34	1	3.532207	1.507928	-1.133764
35	6	6.473184	0.238716	-0.011077
36	1	6.749436	-1.722675	0.840140
37	1	5.915744	2.123430	-0.892562
38	17	8.166911	0.681248	0.154480

QST6

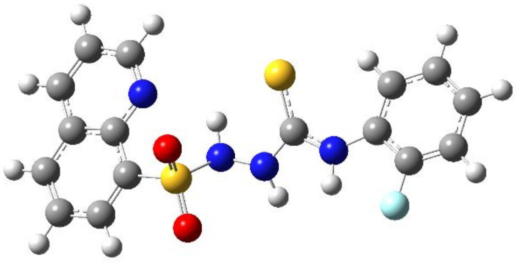

Center Number	Atomic Number	Coordinates (Angstroms) X	Y	Z
1	6	-3.959836	-2.050007	-0.227420
2	6	-3.140703	-0.972864	0.028454
3	6	-3.632303	0.363907	-0.015533
4	6	-5.019676	0.559633	-0.306732
5	6	-5.849127	-0.566928	-0.556199
6	6	-5.329354	-1.842011	-0.524622
7	1	-3.546529	-3.052458	-0.196216
8	6	-5.493204	1.897263	-0.335852
9	1	-6.900972	-0.405364	-0.776864

10	1	-5.965296	-2.698764	-0.722891
11	6	-4.617895	2.931419	-0.095699
12	6	-3.256830	2.626752	0.158512
13	1	-6.541823	2.087323	-0.549469
14	1	-4.946220	3.965486	-0.107452
15	1	-2.541909	3.428357	0.331884
16	16	-1.450430	-1.318981	0.555567
17	8	-1.266987	-0.882183	1.942538
18	8	-1.137519	-2.709044	0.192624
19	7	-2.774706	1.399430	0.195605
20	7	-0.565859	-0.346808	-0.609543
21	1	-0.666957	0.645540	-0.342184
22	7	0.787314	-0.688356	-0.590666
23	1	1.019431	-1.394965	-1.277181
24	6	1.769025	0.188292	-0.167865
25	7	2.999459	-0.415322	-0.233374
26	1	2.981282	-1.427716	-0.289244
27	16	1.416962	1.739247	0.339327
28	6	4.305137	0.100672	-0.160466
29	6	5.341881	-0.846696	-0.160726
30	6	4.675892	1.452930	-0.119094
31	6	6.682450	-0.514880	-0.119680
32	6	6.027257	1.806995	-0.078581
33	1	3.906964	2.211461	-0.105998
34	6	7.032337	0.838502	-0.076391
35	1	7.424369	-1.305954	-0.122204
36	1	6.288825	2.859946	-0.044463
37	1	8.078117	1.125545	-0.040183
38	9	4.983165	-2.168151	-0.215994

QST7

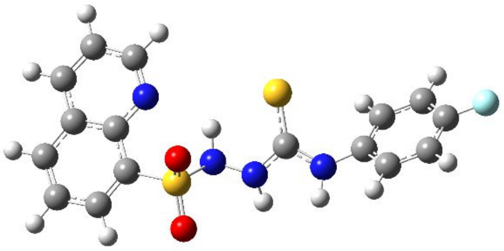

Center Number	Atomic Number	Coordinates (Angstroms) X	Y	Z
1	6	-4.273701	-1.872984	-0.294895
2	6	-3.339947	-0.899188	-0.018095
3	6	-3.699051	0.477767	0.055281
4	6	-5.073940	0.824330	-0.137936
5	6	-6.022526	-0.198050	-0.410468
6	6	-5.629950	-1.515558	-0.495073
7	1	-3.959911	-2.909742	-0.353034
8	6	-5.413517	2.199596	-0.050367
9	1	-7.063763	0.077458	-0.555625
10	1	-6.356978	-2.291924	-0.710543
11	6	-4.428597	3.126290	0.203780

12	6	-3.092647	2.676663	0.355216
13	1	-6.448111	2.503354	-0.187143
14	1	-4.653446	4.185030	0.279338
15	1	-2.293078	3.391577	0.537704
16	16	-1.669199	-1.441397	0.393107
17	8	-1.381190	-1.130698	1.796548
18	8	-1.511172	-2.824942	-0.080303
19	7	-2.733489	1.409487	0.282347
20	7	-0.742320	-0.473610	-0.740980
21	1	-0.750481	0.504035	-0.407057
22	7	0.578169	-0.927997	-0.779456
23	1	0.738986	-1.601529	-1.517445
24	6	1.637136	-0.165757	-0.328273
25	7	2.817422	-0.858939	-0.452878
26	1	2.728487	-1.868260	-0.457233
27	16	1.454021	1.379656	0.270943
28	6	4.139260	-0.376363	-0.262948
29	6	5.040596	-1.170617	0.457011
30	6	4.575018	0.828251	-0.830890
31	6	6.370494	-0.774284	0.611763
32	6	5.896647	1.240424	-0.665779
33	1	3.882757	1.443267	-1.391524
34	6	6.770412	0.430221	0.050119
35	1	7.079655	-1.378101	1.166940
36	1	6.251304	2.172087	-1.092734
37	1	4.701970	-2.098748	0.909128
38	9	8.061307	0.830827	0.203395

QST8

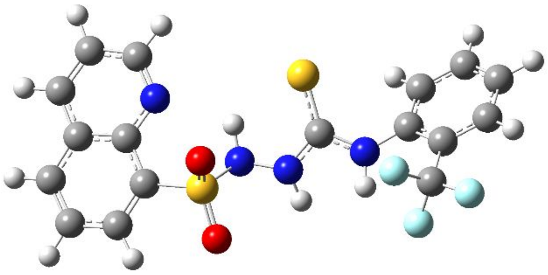

Center Number	Atomic Number	Coordinates (Angstroms) X	Y	Z
1	6	-4.414914	-2.045773	-0.565322
2	6	-3.631878	-0.988744	-0.157566
3	6	-4.190544	0.296621	0.099998
4	6	-5.604917	0.455875	-0.047621
5	6	-6.395500	-0.651674	-0.457226
6	6	-5.812662	-1.872026	-0.718288
7	1	-3.951694	-3.006737	-0.762102
8	6	-6.144029	1.740327	0.224302
9	1	-7.468675	-0.518645	-0.565571
10	1	-6.419837	-2.712748	-1.038056
11	6	-5.303470	2.762058	0.602414
12	6	-3.912909	2.501720	0.694196

13	1	-7.214740	1.900634	0.128260
14	1	-5.681891	3.755443	0.819718
15	1	-3.225786	3.297980	0.972674
16	16	-1.892655	-1.324748	0.182240
17	8	-1.631354	-1.164690	1.615118
18	8	-1.535303	-2.589158	-0.477646
19	7	-3.370308	1.323928	0.452287
20	7	-1.142783	-0.083180	-0.809722
21	1	-1.284817	0.827059	-0.342932
22	7	0.227914	-0.323318	-0.922351
23	1	0.463852	-0.912723	-1.710527
24	6	1.177746	0.516875	-0.390691
25	7	2.444712	0.027810	-0.636069
26	1	2.503142	-0.973032	-0.782011
27	16	0.789452	1.914659	0.432140
28	6	3.680138	0.694873	-0.488934
29	6	4.810302	-0.004518	-0.008811
30	6	3.828616	2.033726	-0.874551
31	6	6.049625	0.640016	0.076036
32	6	5.064323	2.667202	-0.769990
33	1	2.969725	2.571526	-1.253154
34	6	6.181337	1.975702	-0.295965
35	1	6.906686	0.088030	0.443090
36	1	5.153562	3.705860	-1.073604
37	1	7.145426	2.467631	-0.220054
38	6	4.698611	-1.436617	0.441705
39	9	3.887181	-1.583661	1.517089
40	9	4.165957	-2.240475	-0.540933
41	9	5.890591	-1.979039	0.766431

QST9

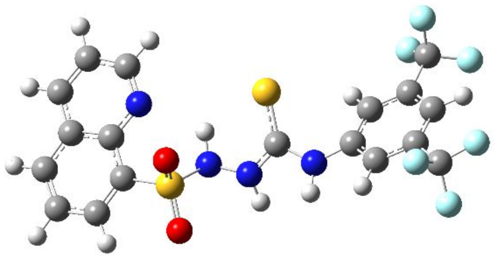

Center Number	Atomic Number	Coordinates (Angstroms) X	Y	Z
1	6	-5.825348	-1.828226	-0.723744
2	6	-4.891318	-0.947687	-0.224400
3	6	-5.251078	0.368574	0.186259
4	6	-6.627824	0.746919	0.094549
5	6	-7.576989	-0.181471	-0.411911
6	6	-7.183539	-1.437050	-0.819410
7	1	-5.511254	-2.818285	-1.036561
8	6	-6.969084	2.057606	0.518969
9	1	-8.619935	0.117117	-0.476372
10	1	-7.911397	-2.139498	-1.212357
11	6	-5.983418	2.897615	0.984001

12	6	-4.645426	2.429862	1.009315
13	1	-8.005359	2.381435	0.469274
14	1	-6.209047	3.904700	1.318566
15	1	-3.845834	3.082022	1.354183
16	16	-3.218045	-1.566244	0.030360
17	8	-2.917044	-1.611120	1.463922
18	8	-3.050104	-2.784602	-0.775658
19	7	-4.284630	1.220148	0.625706
20	7	-2.299218	-0.339919	-0.831324
21	1	-2.316553	0.525207	-0.268469
22	7	-0.978003	-0.761955	-0.985035
23	1	-0.821652	-1.272820	-1.844705
24	6	0.080155	-0.153432	-0.353819
25	7	1.259485	-0.814156	-0.650102
26	1	1.157639	-1.800764	-0.858105
27	16	-0.083320	1.185248	0.621637
28	6	2.581665	-0.401379	-0.387549
29	6	3.518633	-1.374619	-0.030275
30	6	2.993333	0.931609	-0.543532
31	6	4.857235	-1.022351	0.168159
32	6	4.327135	1.265432	-0.326905
33	1	2.280815	1.690938	-0.832732
34	6	5.274055	0.298226	0.030738
35	1	6.310204	0.570133	0.189146
36	1	3.206470	-2.406749	0.100229
37	6	5.835315	-2.109831	0.534991
38	6	4.771934	2.702228	-0.452262
39	9	5.924704	-3.050564	-0.443544
40	9	7.083106	-1.637419	0.747087
41	9	5.456656	-2.766465	1.661579
42	9	5.967515	2.804035	-1.086531
43	9	4.930223	3.284498	0.764882
44	9	3.887671	3.460060	-1.140576

QST10

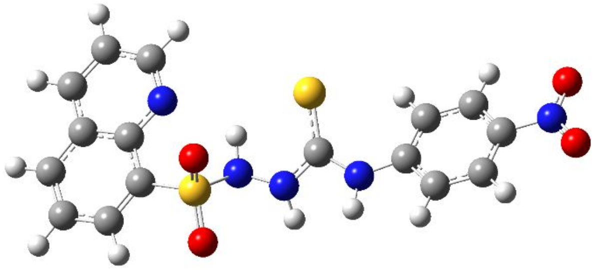

Center Number	Atomic Number	Coordinates (Angstroms) X	Y	Z
1	6	-4.871886	-1.882209	-0.295980
2	6	-3.955266	-0.891791	-0.020360
3	6	-4.330366	0.482304	0.020566
4	6	-5.705225	0.808510	-0.204569
5	6	-6.636673	-0.230075	-0.475050

6	6	-6.228007	-1.544367	-0.527605
7	1	-4.545830	-2.916279	-0.328972
8	6	-6.062694	2.180933	-0.149813
9	1	-7.678042	0.030423	-0.644537
10	1	-6.942036	-2.332989	-0.741656
11	6	-5.093737	3.124114	0.104843
12	6	-3.755841	2.693763	0.289666
13	1	-7.097945	2.469352	-0.311859
14	1	-5.332241	4.181270	0.155849
15	1	-2.969029	3.422311	0.473434
16	16	-2.287629	-1.404532	0.431126
17	8	-2.026665	-1.068592	1.833444
18	8	-2.093166	-2.789570	-0.022934
19	7	-3.379813	1.429742	0.247444
20	7	-1.349220	-0.436258	-0.697739
21	1	-1.376329	0.544003	-0.375934
22	7	-0.027249	-0.879882	-0.716397
23	1	0.144554	-1.583390	-1.423437
24	6	1.021990	-0.136269	-0.225684
25	7	2.195491	-0.863887	-0.308988
26	1	2.066382	-1.869226	-0.303169
27	16	0.834426	1.400268	0.385359
28	6	3.535373	-0.459890	-0.206579
29	6	4.477117	-1.458348	0.111335
30	6	3.976850	0.850977	-0.458207
31	6	5.830960	-1.162577	0.181747
32	6	5.332630	1.149976	-0.384620
33	1	3.265341	1.627202	-0.700046
34	6	6.248371	0.146342	-0.065517
35	1	4.141213	-2.472398	0.311800
36	1	6.560414	-1.923476	0.429626
37	1	5.687870	2.154619	-0.577932
38	7	7.674757	0.469611	0.008769
39	8	8.014129	1.635858	-0.207725
40	8	8.459153	-0.443551	0.281767

QST11

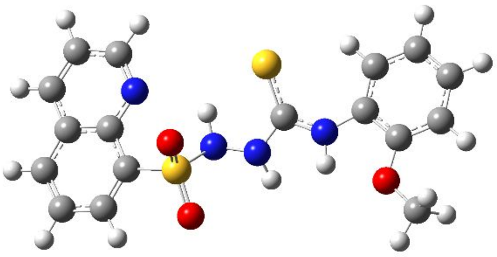

Center Number	Atomic Number	Coordinates (Angstroms) X	Y	Z
1	6	-4.090952	-2.136902	-0.226758
2	6	-3.324683	-1.021860	0.030246
3	6	-3.881940	0.288975	-0.009723
4	6	-5.277499	0.417708	-0.299404

5	6	-6.051339	-0.747481	-0.549940
6	6	-5.469635	-1.995624	-0.521226
7	1	-3.628491	-3.117763	-0.198767
8	6	-5.814957	1.731012	-0.326571
9	1	-7.110100	-0.637262	-0.769289
10	1	-6.063293	-2.882100	-0.720219
11	6	-4.990545	2.806056	-0.085771
12	6	-3.616175	2.567205	0.167970
13	1	-6.871682	1.870571	-0.539428
14	1	-5.368557	3.823047	-0.096616
15	1	-2.940026	3.401627	0.342174
16	16	-1.616698	-1.286450	0.551949
17	8	-1.454257	-0.848320	1.941602
18	8	-1.241653	-2.660663	0.185622
19	7	-3.075935	1.364510	0.203439
20	7	-0.785145	-0.269831	-0.611251
21	1	-0.939835	0.716334	-0.343748
22	7	0.585697	-0.536366	-0.586761
23	1	0.859169	-1.210401	-1.290528
24	6	1.515207	0.410204	-0.180834
25	7	2.774840	-0.115837	-0.242482
26	1	2.823211	-1.128699	-0.289819
27	16	1.061247	1.944365	0.302678
28	6	4.054912	0.466383	-0.160547
29	6	5.132050	-0.456406	-0.126308
30	6	4.337367	1.834455	-0.140329
31	6	6.448746	-0.007899	-0.067460
32	6	5.663327	2.278256	-0.083194
33	1	3.521226	2.542246	-0.155094
34	6	6.714753	1.367426	-0.044336
35	1	7.268434	-0.715726	-0.038674
36	1	5.861351	3.345293	-0.064635
37	1	7.743153	1.711521	0.005325
38	8	4.759192	-1.779150	-0.165541
39	6	5.768260	-2.781916	-0.095140
40	1	6.336141	-2.702792	0.839140
41	1	5.241457	-3.736255	-0.121601
42	1	6.451224	-2.716099	-0.950390

QST12

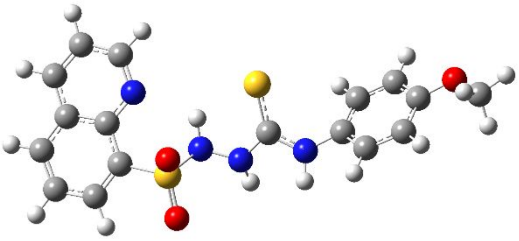

Center Number	Atomic Number	Coordinates (Angstroms) X	Y	Z
1	6	-4.674638	-1.833921	-0.263018
2	6	-3.719141	-0.879727	0.007487
3	6	-4.051475	0.503450	0.089904

4	6	-5.421300	0.877393	-0.088039
5	6	-6.392291	-0.125414	-0.354268
6	6	-6.025863	-1.449859	-0.447477
7	1	-4.381169	-2.876167	-0.328646
8	6	-5.733044	2.258742	0.007568
9	1	-7.429478	0.170641	-0.487720
10	1	-6.769913	-2.211335	-0.658133
11	6	-4.727684	3.165231	0.254546
12	6	-3.399269	2.688985	0.391219
13	1	-6.762889	2.583090	-0.117613
14	1	-4.931018	4.227941	0.335597
15	1	-2.583753	3.387201	0.567857
16	16	-2.053687	-1.456840	0.396602
17	8	-1.744546	-1.156297	1.798031
18	8	-1.933587	-2.843098	-0.080361
19	7	-3.066007	1.415396	0.310788
20	7	-1.124376	-0.505906	-0.746688
21	1	-1.107299	0.471394	-0.410329
22	7	0.188073	-0.985002	-0.803807
23	1	0.327798	-1.639503	-1.563063
24	6	1.265131	-0.223311	-0.385417
25	7	2.437309	-0.916388	-0.539009
26	1	2.349866	-1.925649	-0.560011
27	16	1.105448	1.327925	0.208247
28	6	3.763023	-0.424189	-0.374486
29	6	4.662613	-1.148996	0.406789
30	6	4.204982	0.732190	-1.035119
31	6	5.995262	-0.740152	0.536107
32	6	5.519867	1.153956	-0.897265
33	1	3.514676	1.300865	-1.646530
34	6	6.426145	0.421581	-0.113064
35	1	6.668593	-1.325861	1.149931
36	1	5.871380	2.049881	-1.398170
37	1	4.326161	-2.038998	0.932063
38	8	7.697202	0.920983	-0.051748
39	6	8.659822	0.232256	0.736268
40	1	9.584281	0.803283	0.642750
41	1	8.360348	0.195104	1.791253
42	1	8.823851	-0.787945	0.366400

QST13

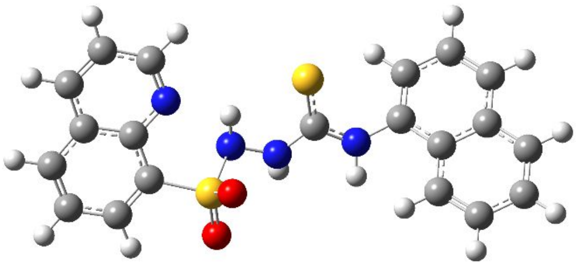

Center Number	Atomic Number	Coordinates (Angstroms) X	Y	Z

1	6	-4.562670	-1.988958	-0.605814
2	6	-3.713306	-0.994731	-0.173564
3	6	-4.202450	0.288896	0.205630
4	6	-5.614948	0.512219	0.153728
5	6	-6.473614	-0.532021	-0.283832
6	6	-5.958319	-1.751986	-0.662504
7	1	-4.152174	-2.950002	-0.896699
8	6	-6.082748	1.793889	0.545119
9	1	-7.544644	-0.350705	-0.318837
10	1	-6.617545	-2.544061	-1.002859
11	6	-5.179603	2.752862	0.942878
12	6	-3.798312	2.433371	0.934044
13	1	-7.149401	2.001701	0.523648
14	1	-5.502663	3.741937	1.250492
15	1	-3.062795	3.180184	1.225600
16	16	-1.971759	-1.420427	0.034049
17	8	-1.619838	-1.376187	1.456705
18	8	-1.713153	-2.652105	-0.727355
19	7	-3.321287	1.255983	0.579299
20	7	-1.227532	-0.144104	-0.911823
21	1	-1.316848	0.738893	-0.381755
22	7	0.130417	-0.424028	-1.092982
23	1	0.314253	-0.902258	-1.965953
24	6	1.123567	0.371370	-0.551215
25	7	2.363672	-0.129166	-0.855505
26	1	2.392113	-1.108164	-1.114593
27	16	0.803108	1.741195	0.344935
28	6	3.627011	0.494346	-0.669590
29	6	4.661463	-0.243539	-0.010309
30	6	3.870095	1.758591	-1.168160
31	6	4.457022	-1.528352	0.566361
32	6	5.966415	0.348961	0.078778
33	6	5.147882	2.346223	-1.036659
34	1	3.071355	2.300105	-1.661230
35	6	5.494533	-2.208954	1.168432
36	1	3.465553	-1.969990	0.565877
37	6	7.013015	-0.384809	0.702706
38	6	6.178778	1.654187	-0.439842
39	1	5.313429	3.344970	-1.428962
40	6	6.788801	-1.637675	1.229543
41	1	5.314122	-3.184287	1.610520
42	1	7.999690	0.067368	0.760915
43	1	7.166845	2.098395	-0.355973
44	1	7.598451	-2.184168	1.703970

QST14

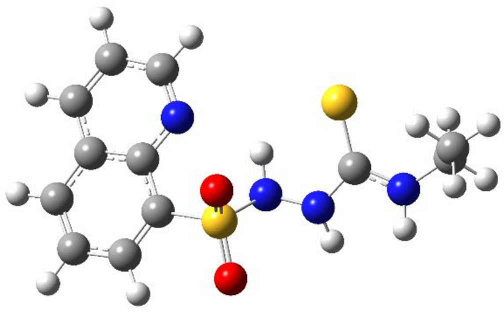

Center Number	Atomic Number	Coordinates (Angstroms) X	Y	Z
1	6	-2.792723	-2.046410	-0.136375
2	6	-1.930240	-0.988045	0.044442
3	6	-2.396456	0.358561	0.047405
4	6	-3.800323	0.584969	-0.113973
5	6	-4.673031	-0.522974	-0.287254
6	6	-4.179210	-1.808625	-0.305728
7	1	-2.399556	-3.057391	-0.144171
8	6	-4.245270	1.932616	-0.097223
9	1	-5.737224	-0.338812	-0.409080
10	1	-4.848741	-2.651288	-0.445460
11	6	-3.329341	2.947011	0.062418
12	6	-1.957397	2.612495	0.189533
13	1	-5.304827	2.145695	-0.212717
14	1	-3.635094	3.987895	0.082135
15	1	-1.211411	3.397187	0.296693
16	16	-0.206996	-1.372675	0.421071
17	8	0.101047	-0.953314	1.792127
18	8	0.039575	-2.768820	0.028931
19	7	-1.501529	1.374944	0.180458
20	7	0.594925	-0.412188	-0.806988
21	1	0.513513	0.582026	-0.536563
22	7	1.950602	-0.755426	-0.865021
23	1	2.139668	-1.445761	-1.580617
24	6	2.948738	0.144990	-0.539371
25	7	4.190580	-0.377088	-0.726745
26	1	4.255621	-1.386649	-0.782669
27	16	2.626942	1.706817	-0.023143
28	6	5.441436	0.337280	-0.470217
29	1	6.185997	-0.059567	-1.169402
30	1	5.270360	1.385871	-0.720897
31	6	5.931626	0.204072	0.974138
32	1	6.873850	0.748540	1.100984
33	1	5.196076	0.619826	1.667770
34	1	6.108758	-0.844128	1.239977

----

^{1}H NMR and ^{13}C NMR spectra of quinoline-8-sulfonohydrazide and **QST1- QST14**

Quinoline-8-sulfonohydrazide (II)

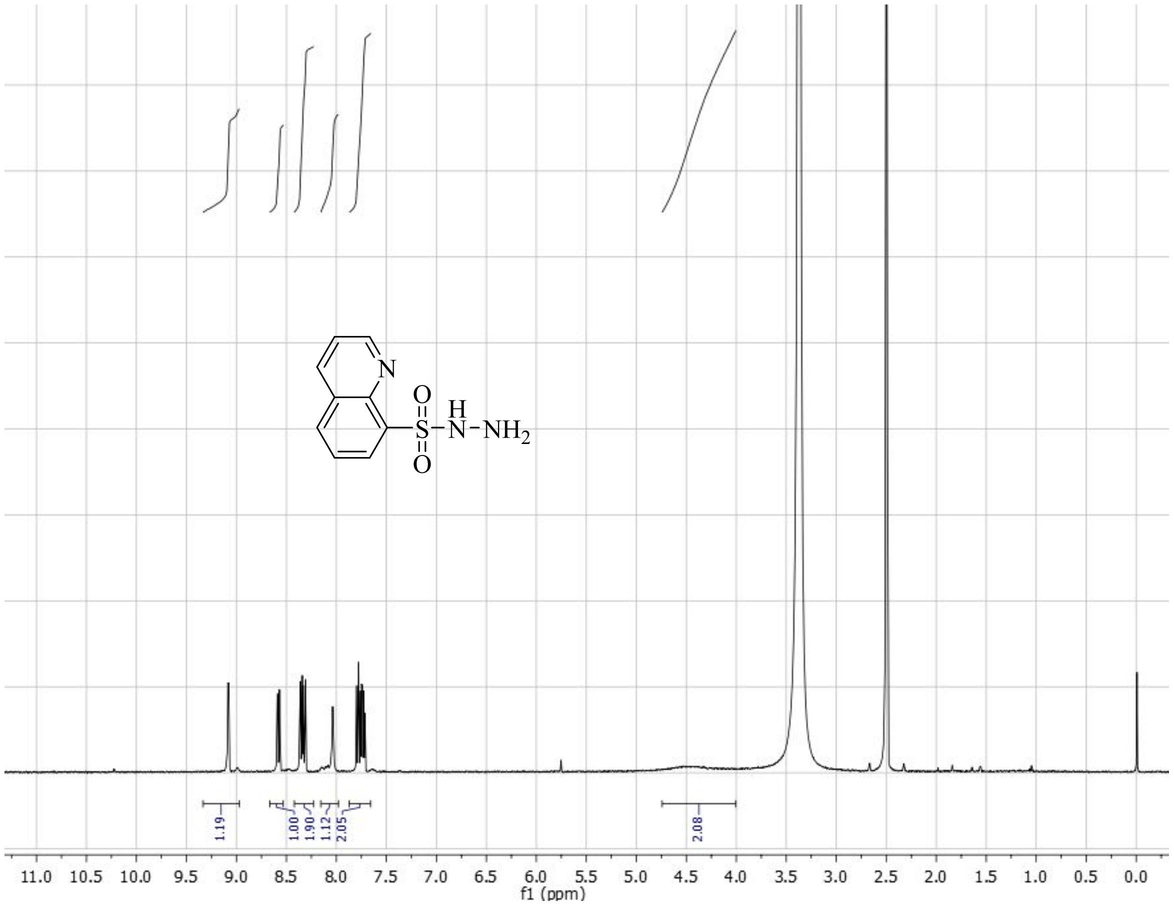
N
O
S
O
H
N
NH2
1.19
1.00
1.90
1.12
2.05
2.08
11.0 10.5 10.0 9.5 9.0 8.5 8.0 7.5 7.0 6.5 6.0 5.5 5.0 4.5 4.0 3.5 3.0 2.5 2.0 1.5 1.0 0.5 0.0
f1 (ppm)

^{1}H NMR, ^{13}C NMR and IR spectra of **QST1**.

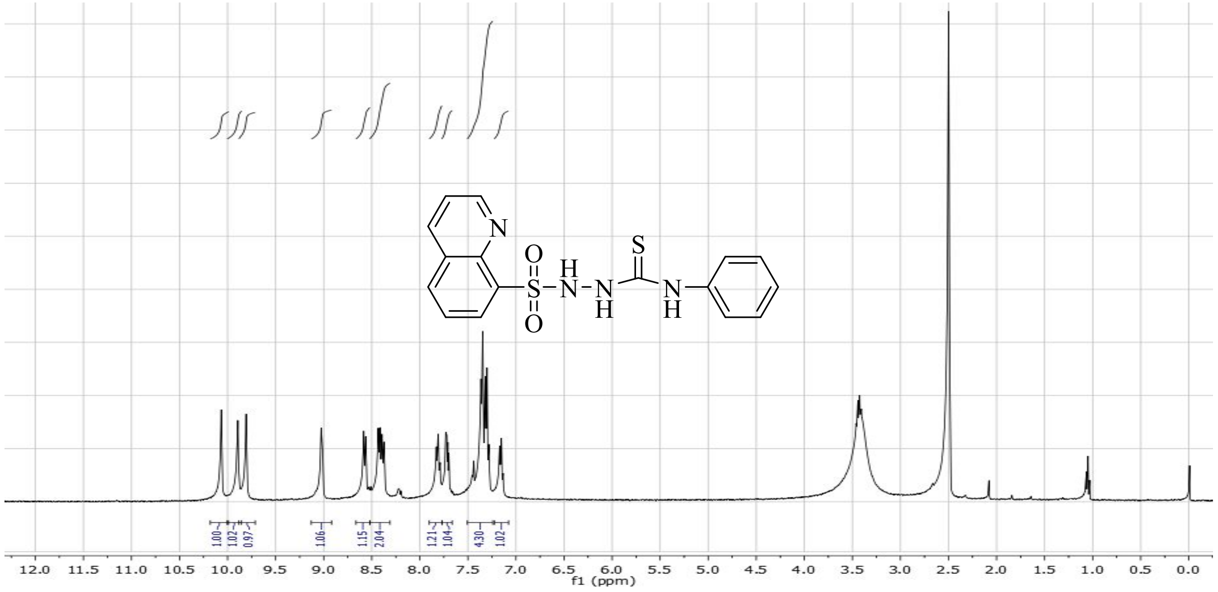

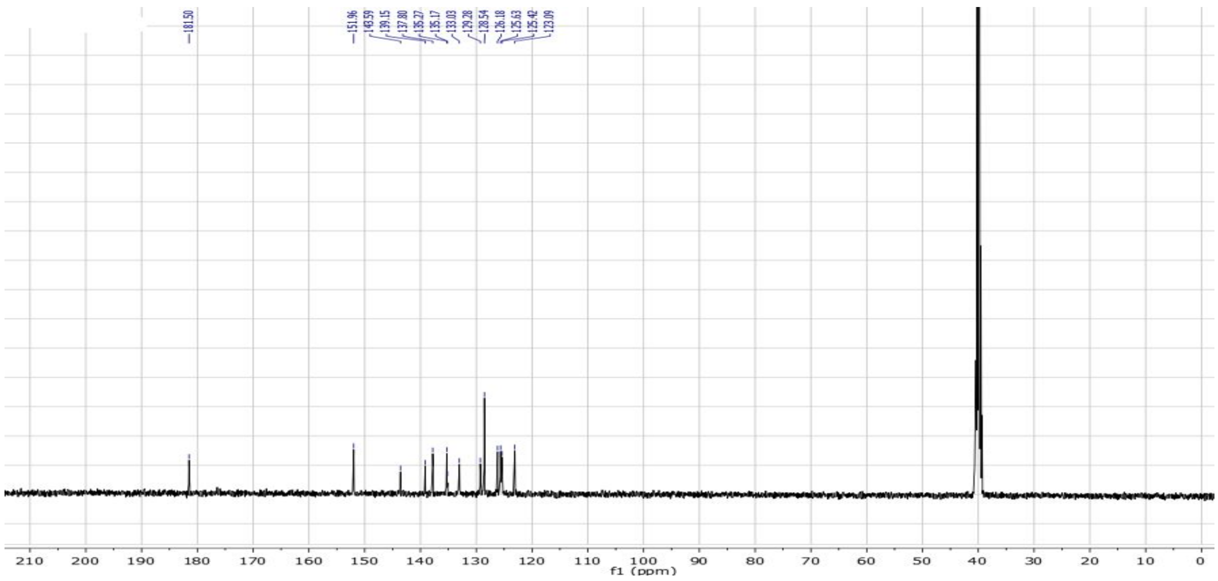

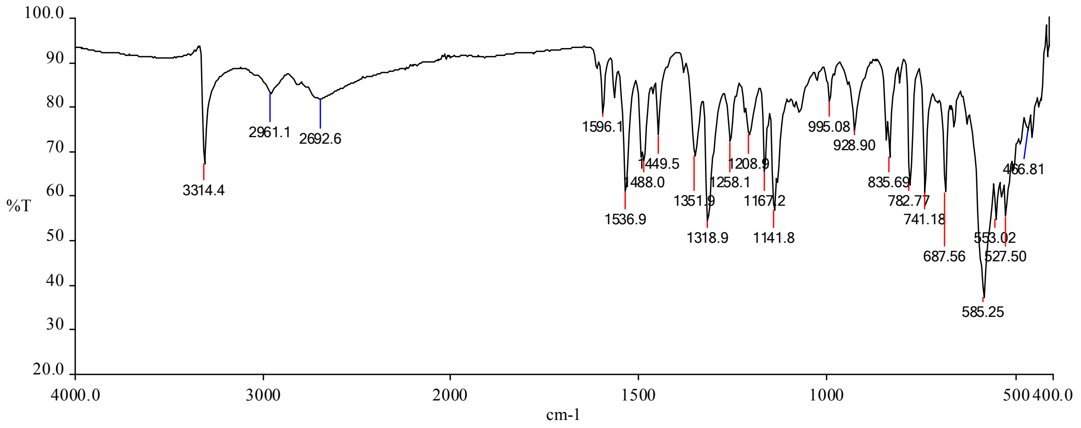

^{1}H NMR, ^{13}C NMR and IR spectra of **QST2**.

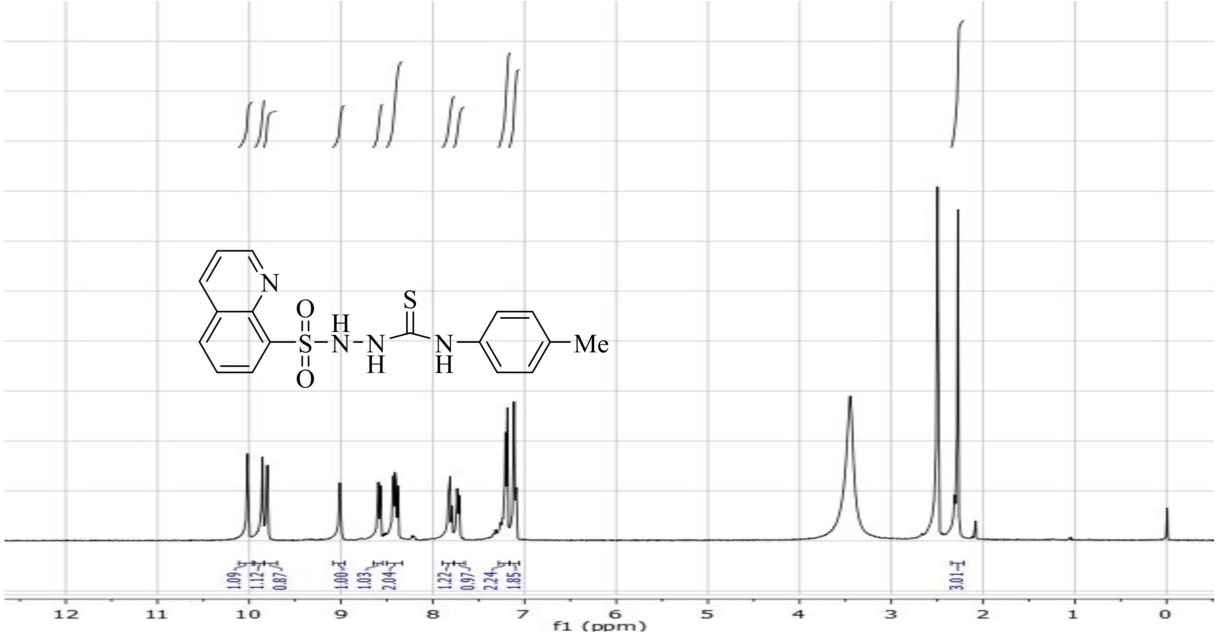

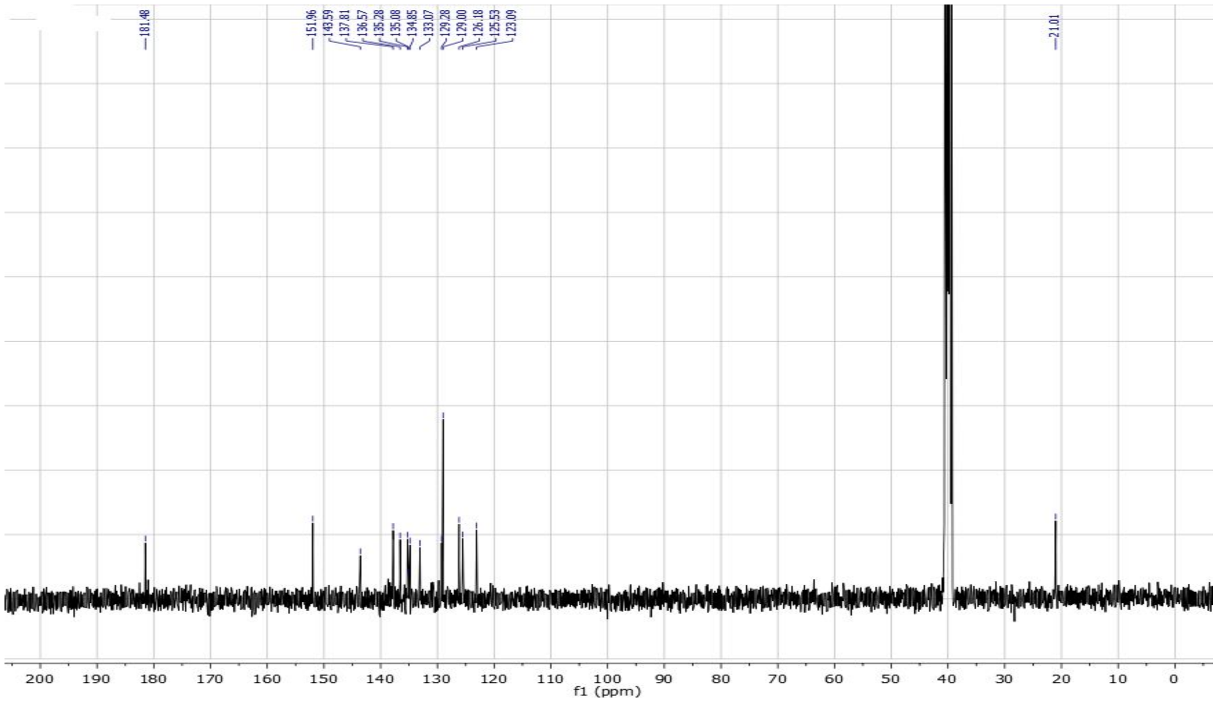

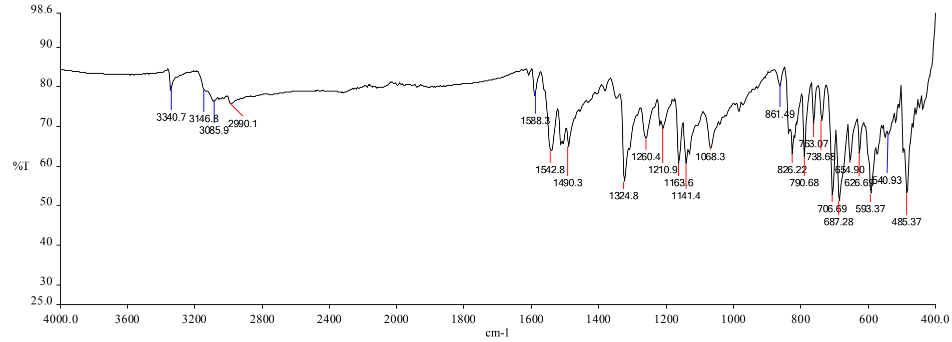

^{1}H NMR, ^{13}C NMR and IR spectra of **QST3**.

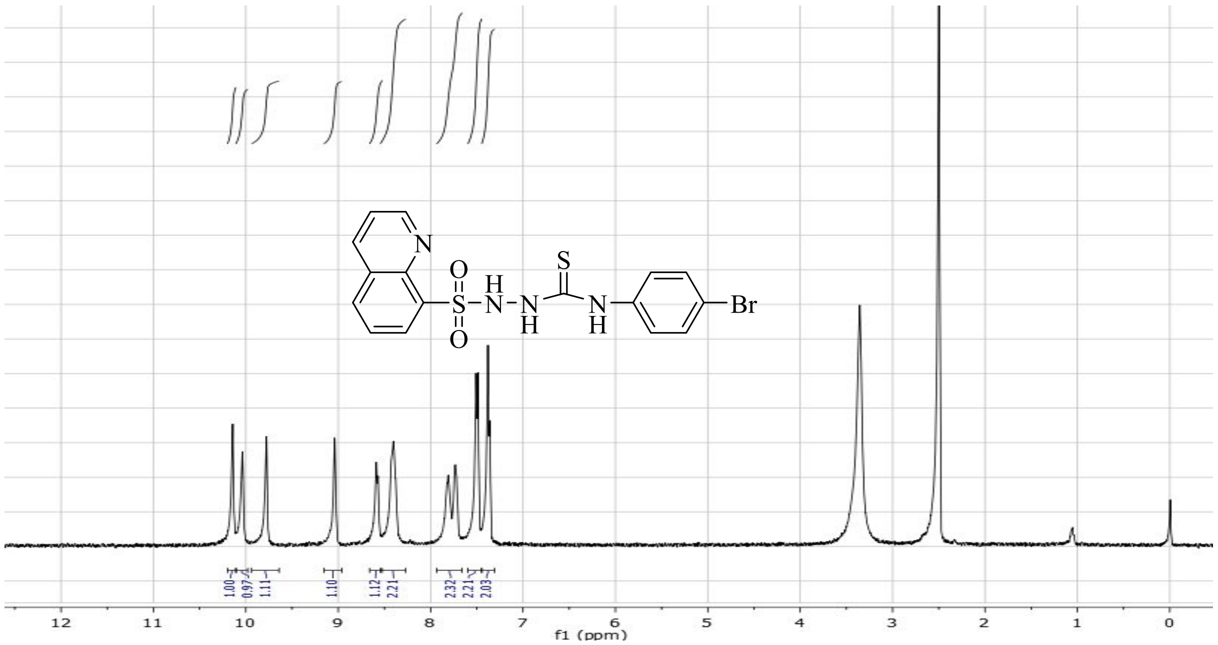

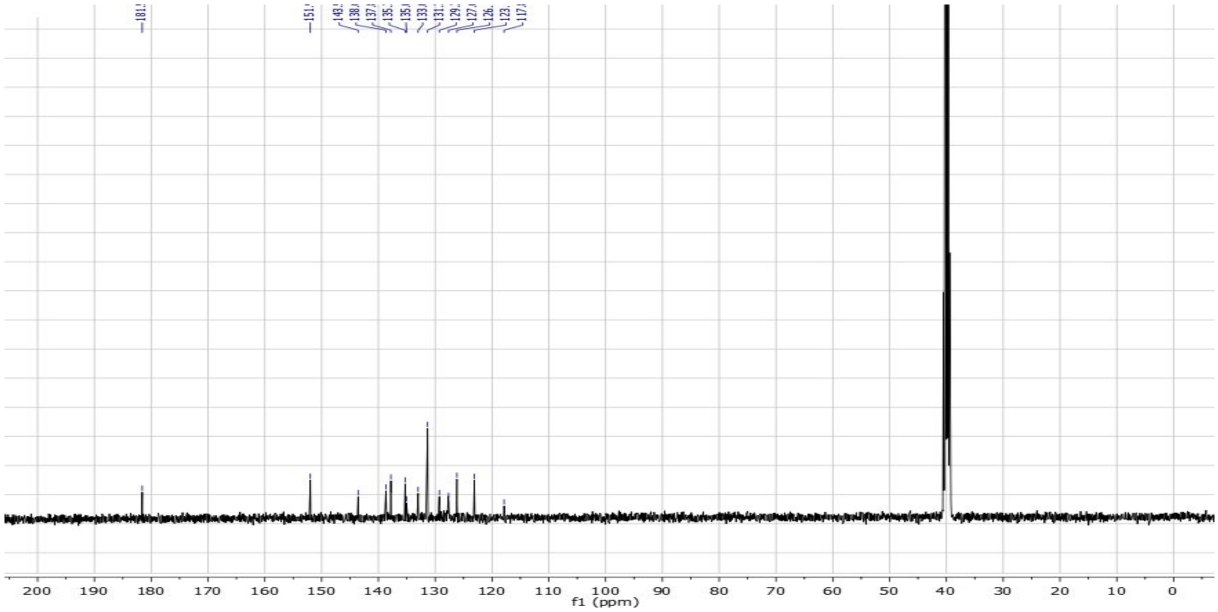

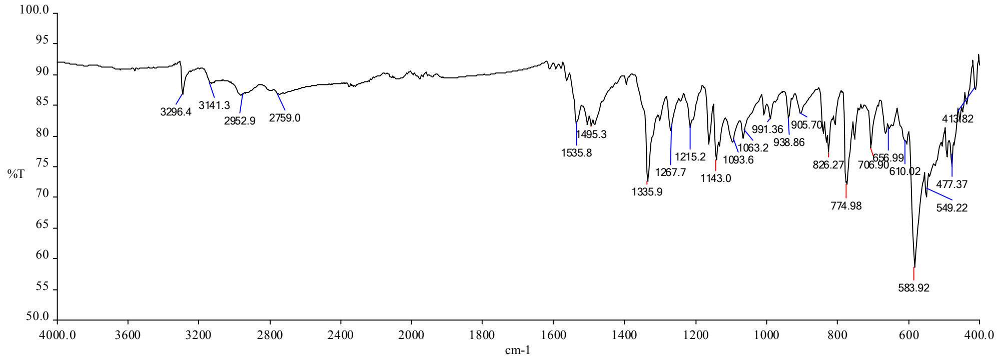

^{1}H NMR, ^{13}C NMR and IR spectra of **QST4**.

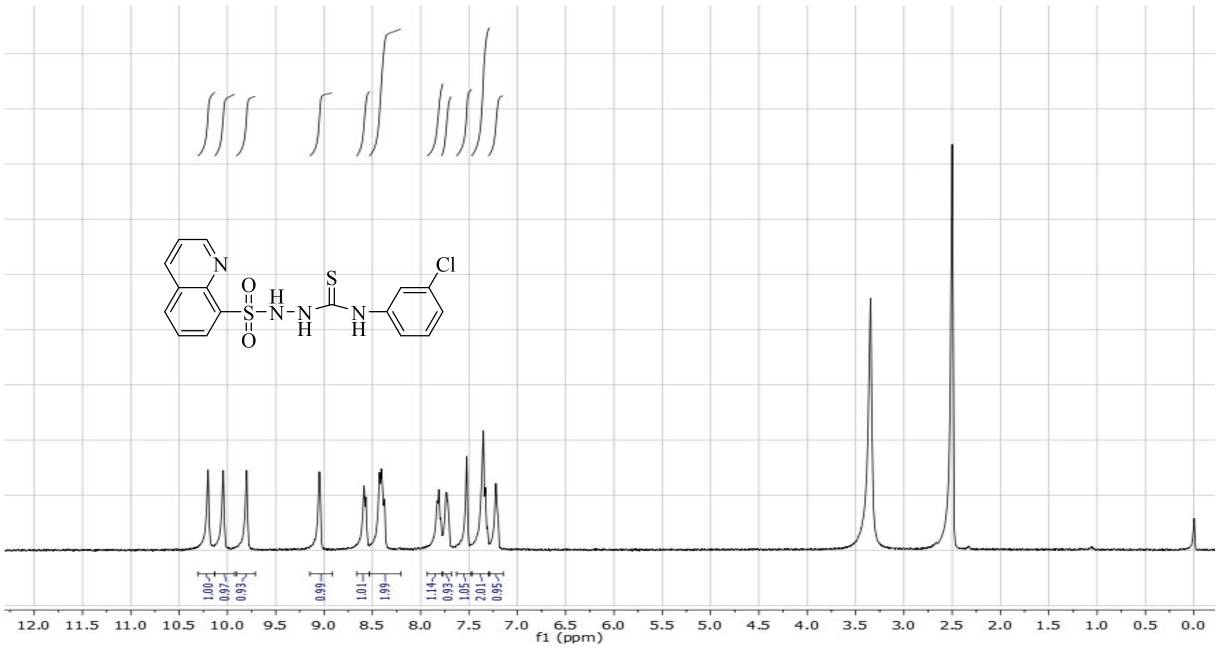

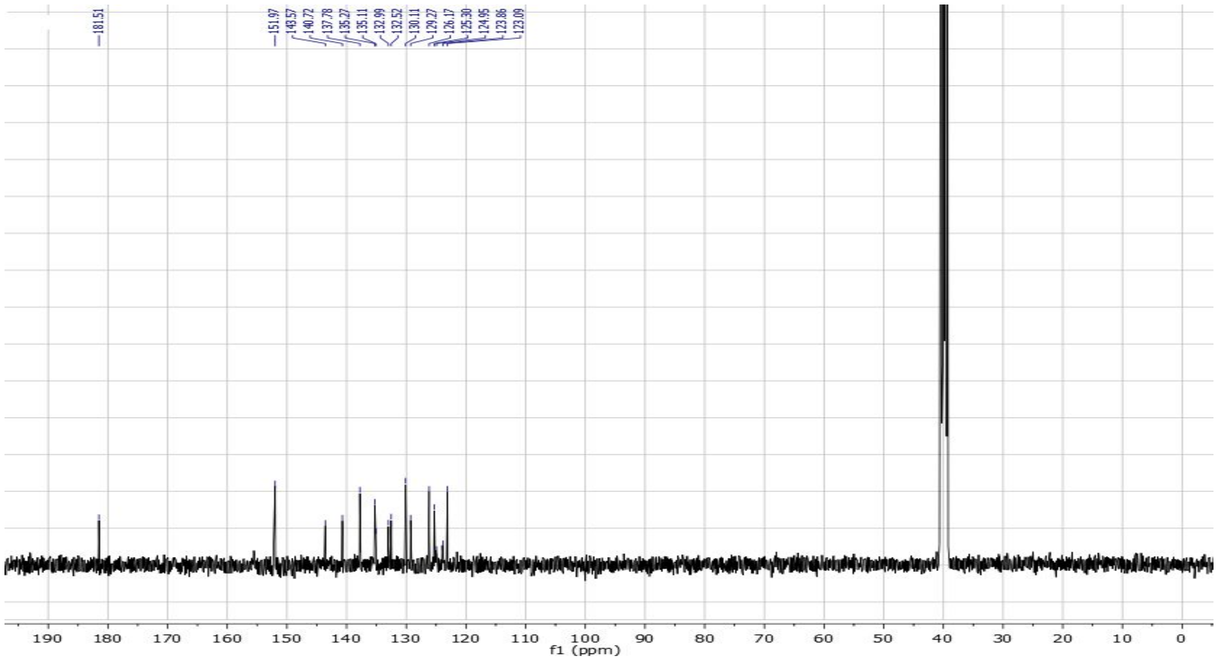

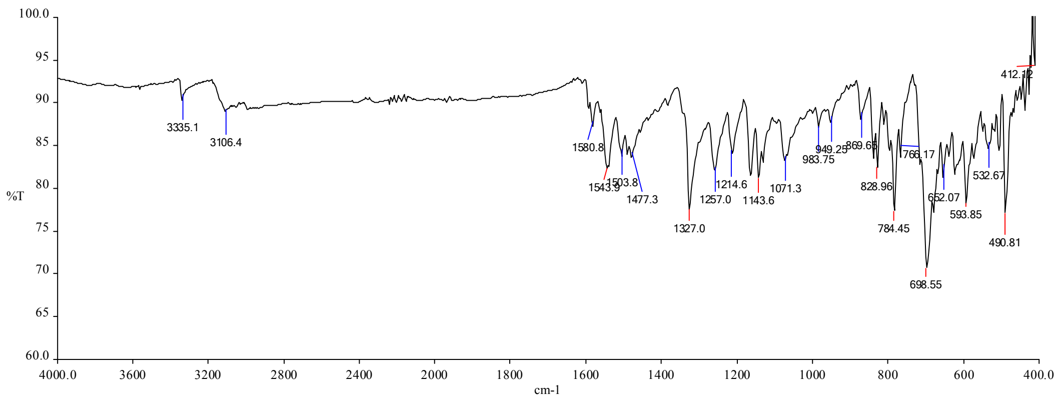

^{1}H NMR, ^{13}C NMR and IR spectra of **QST5**

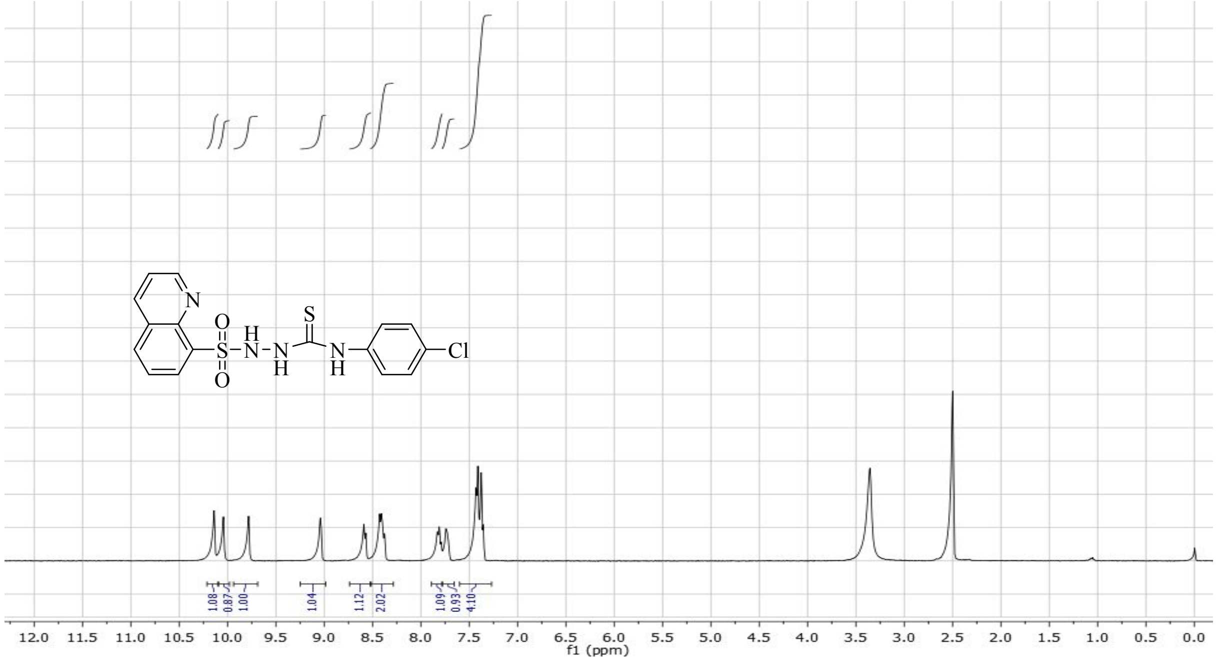

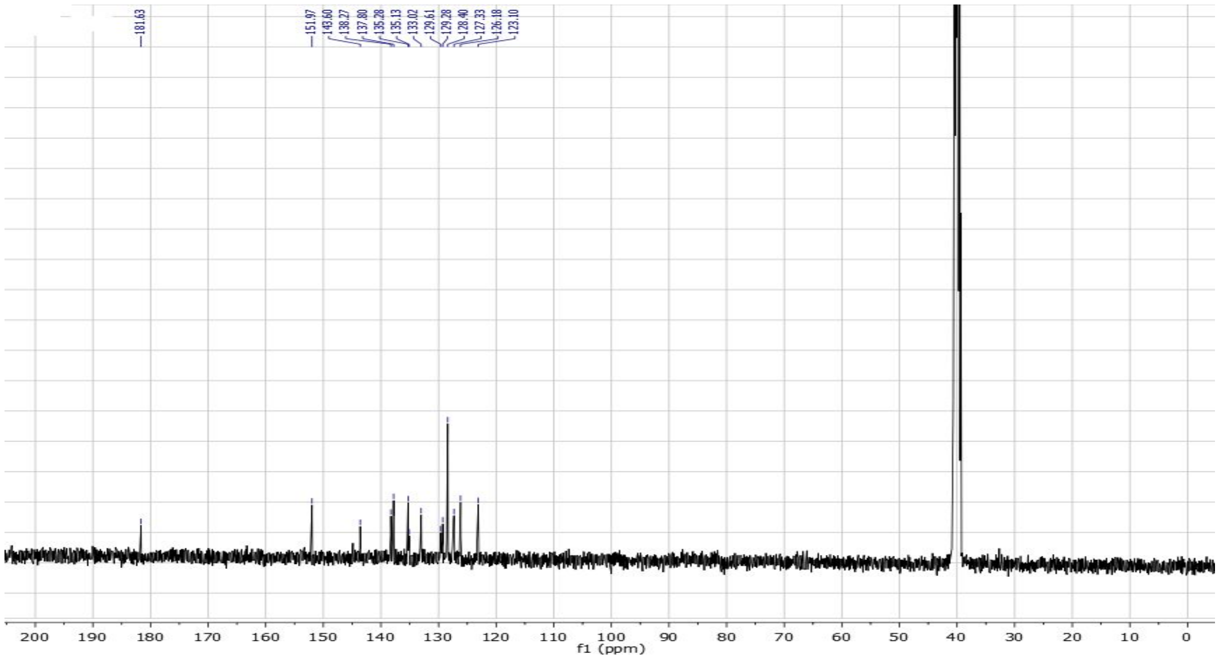

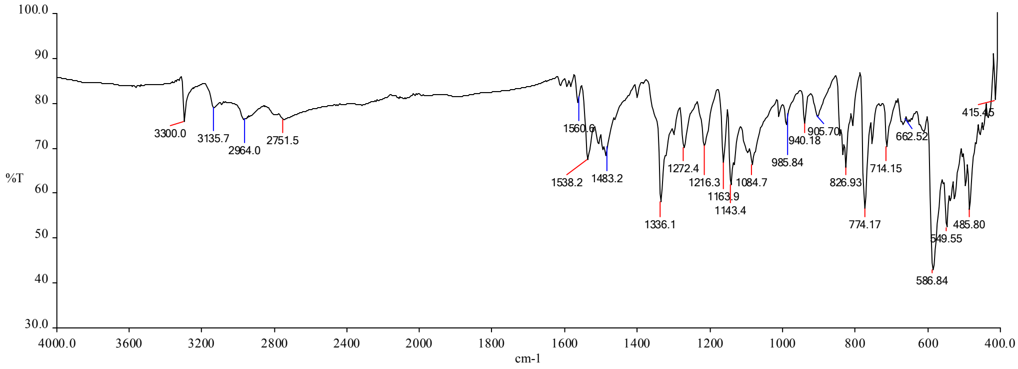

^{1}H NMR, ^{13}C NMR and IR spectra of **QST6**

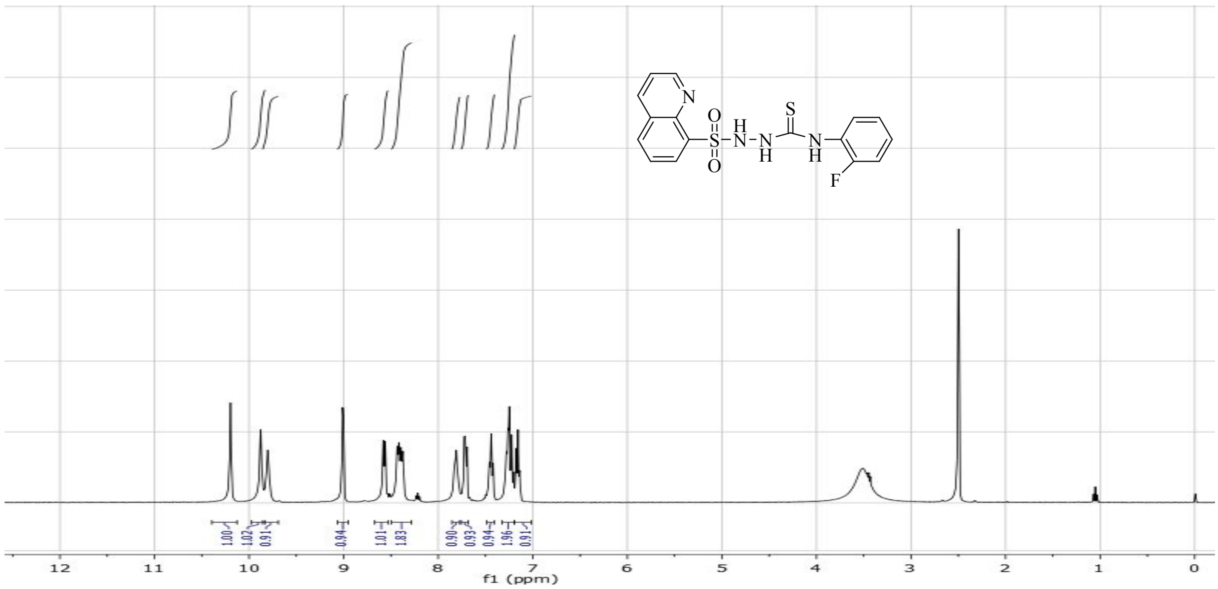

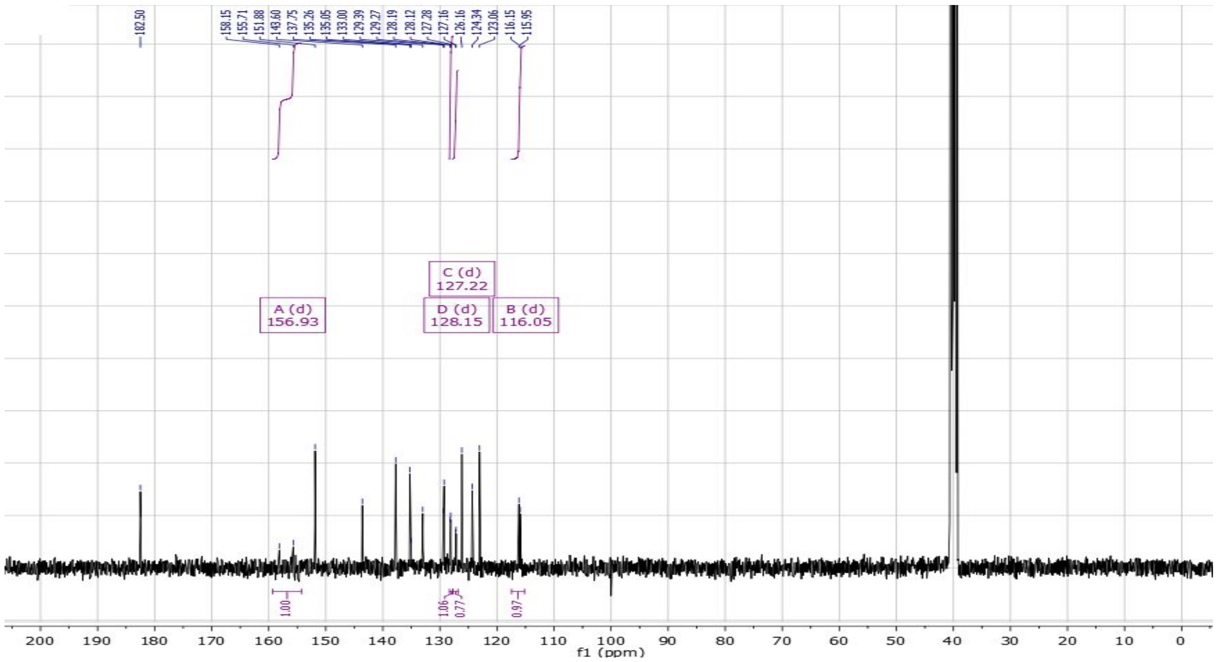

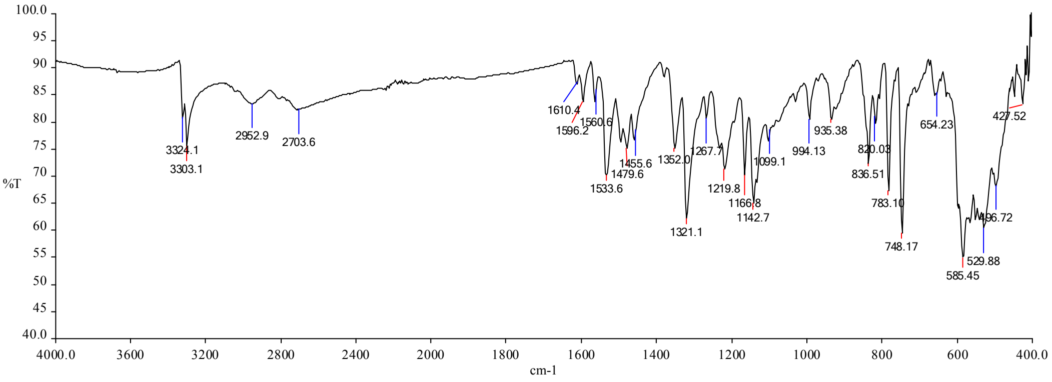

^{1}H NMR, ^{13}C NMR and IR spectra of **QST7**

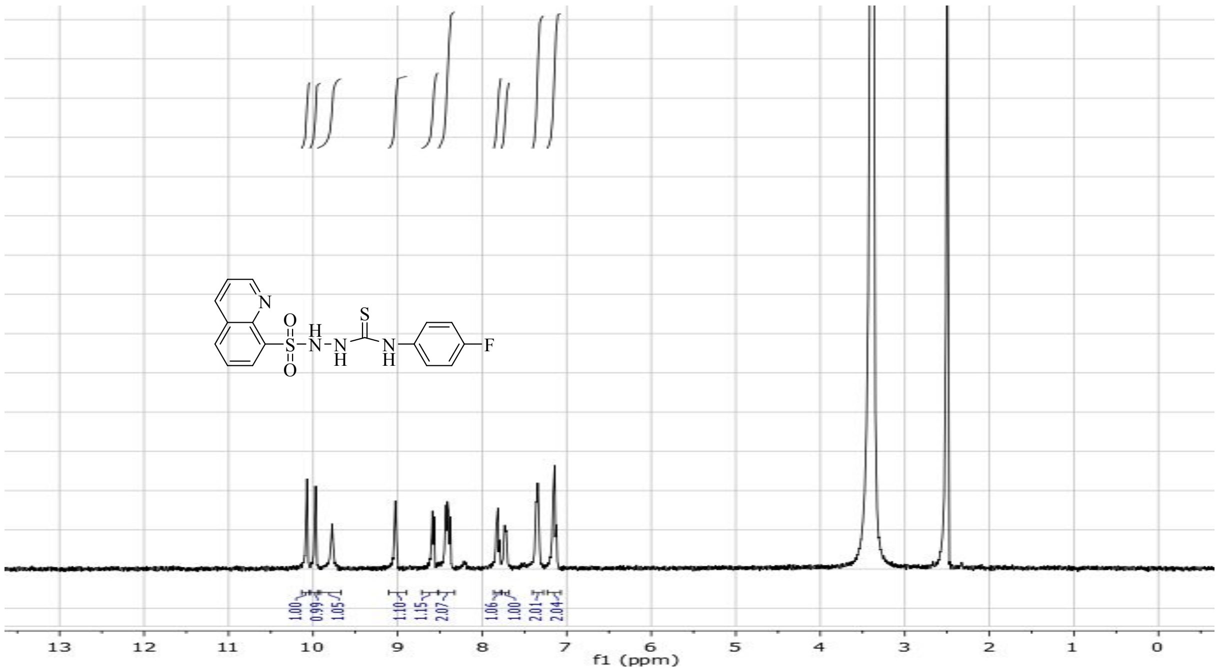

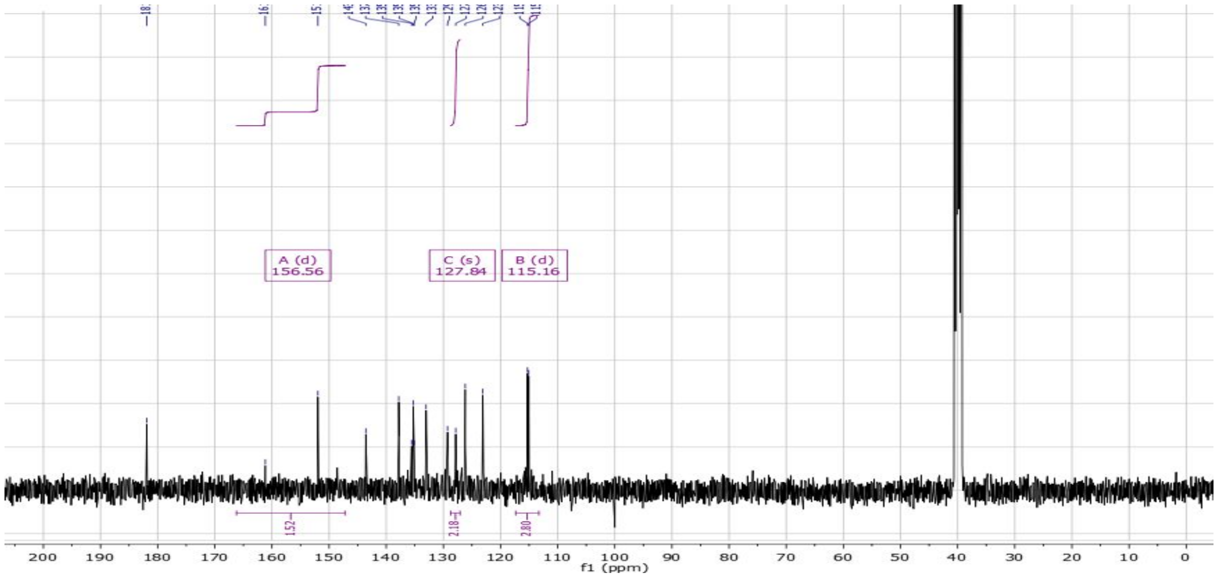

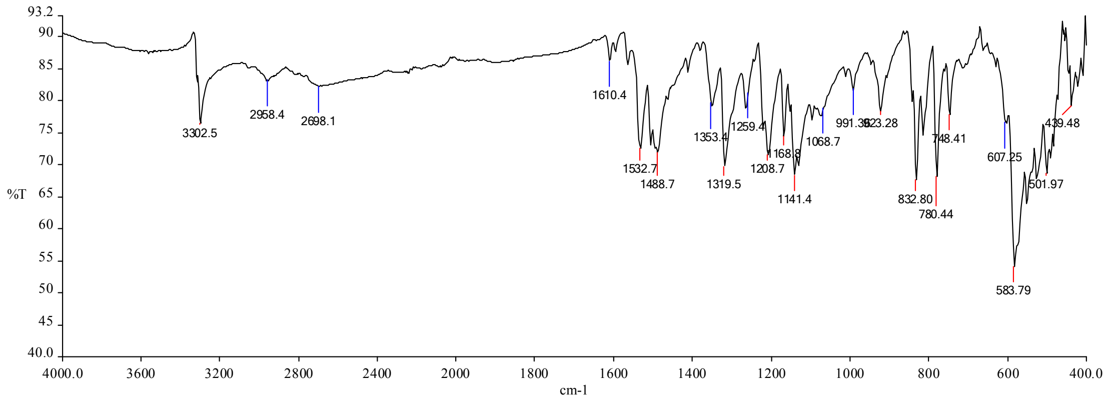

^{1}H NMR, ^{13}C NMR and IR spectra of **QST8**

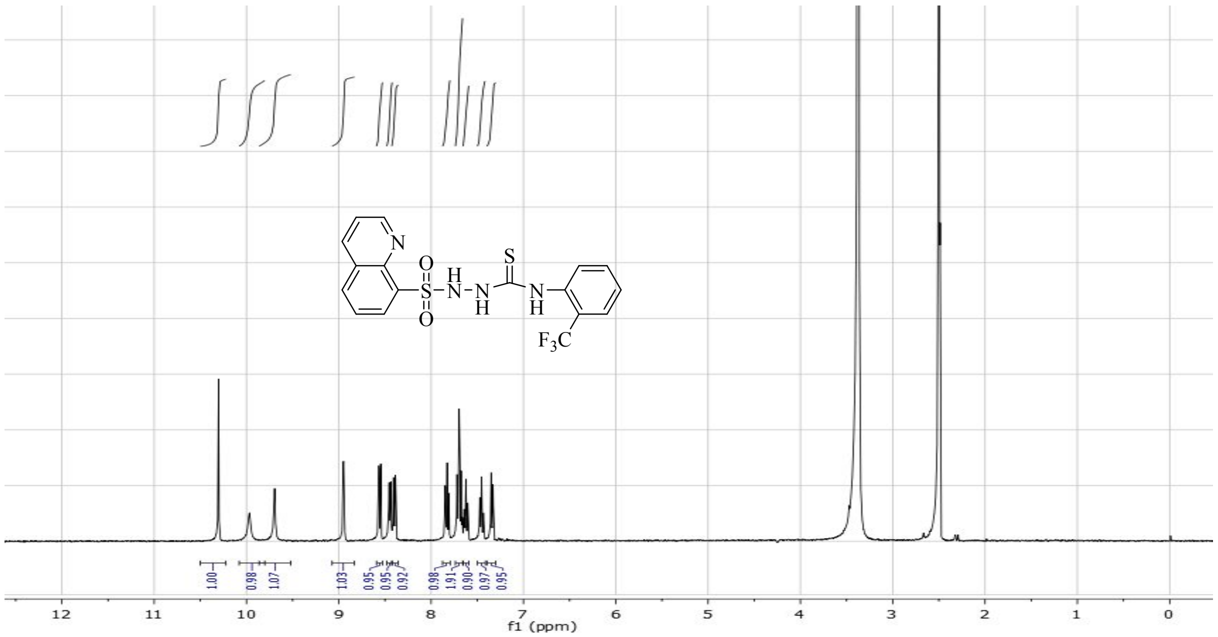

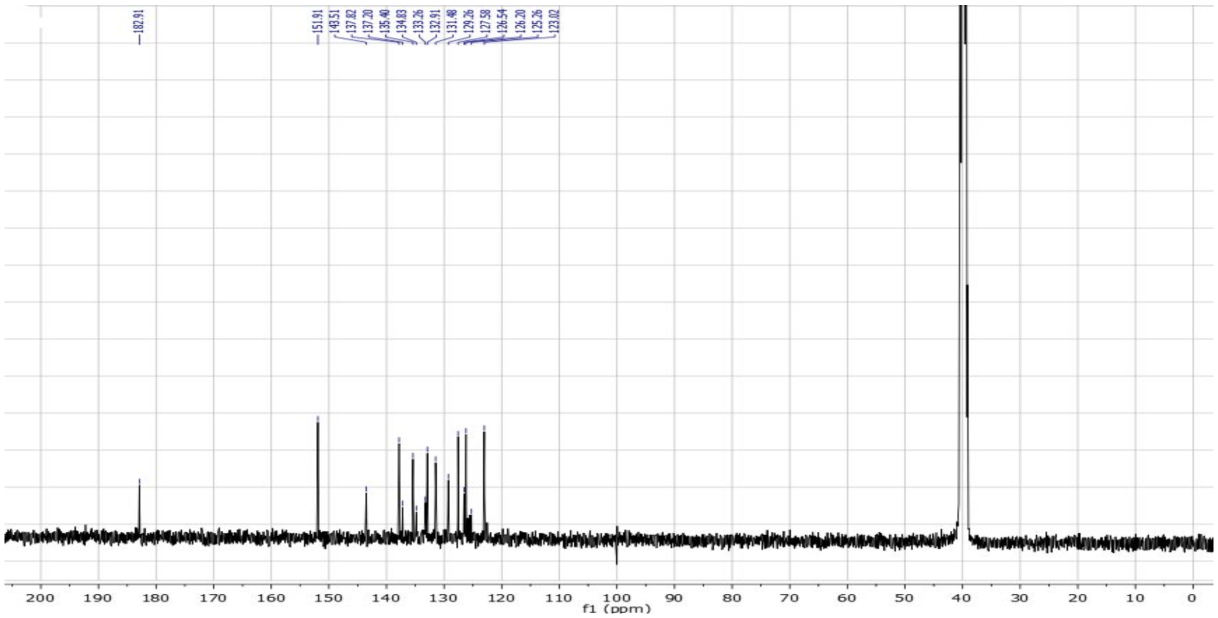

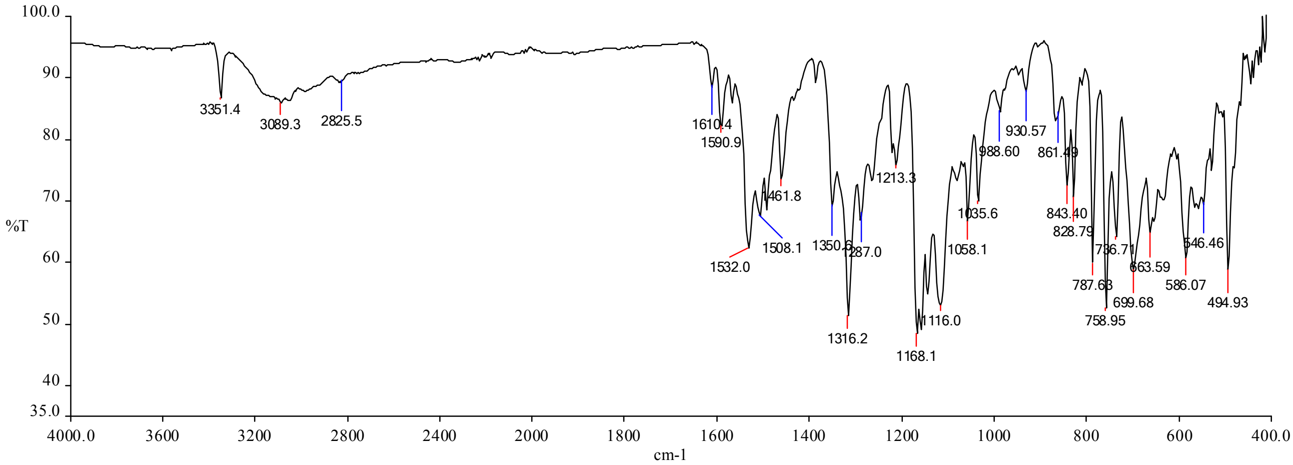

^{1}H NMR, ^{13}C NMR and IR spectra of **QST9**

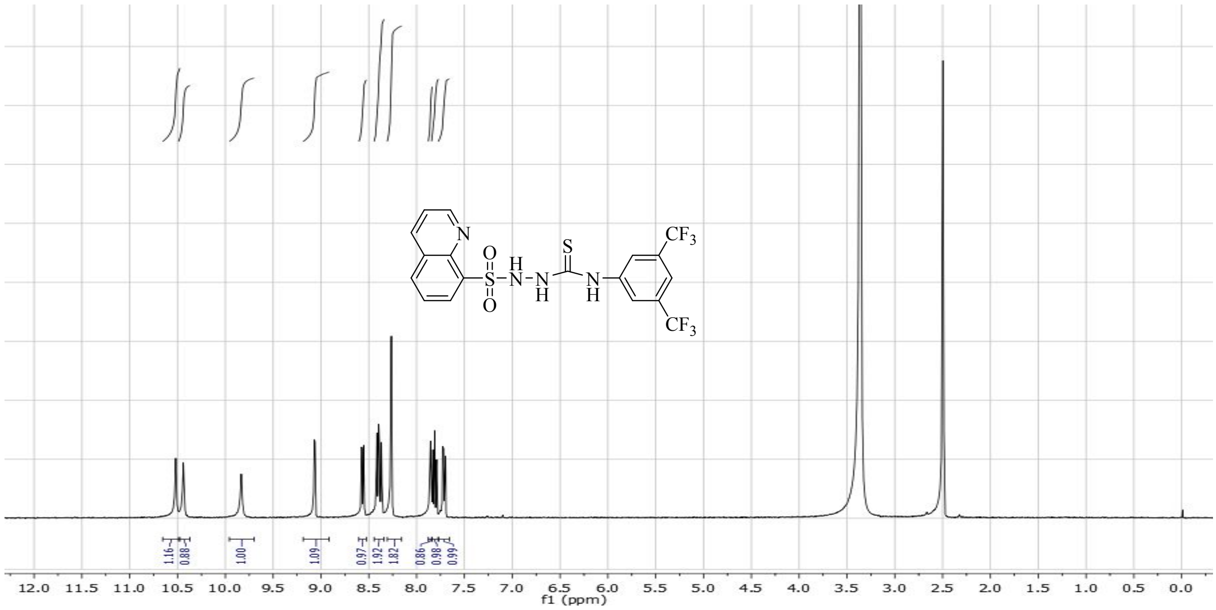

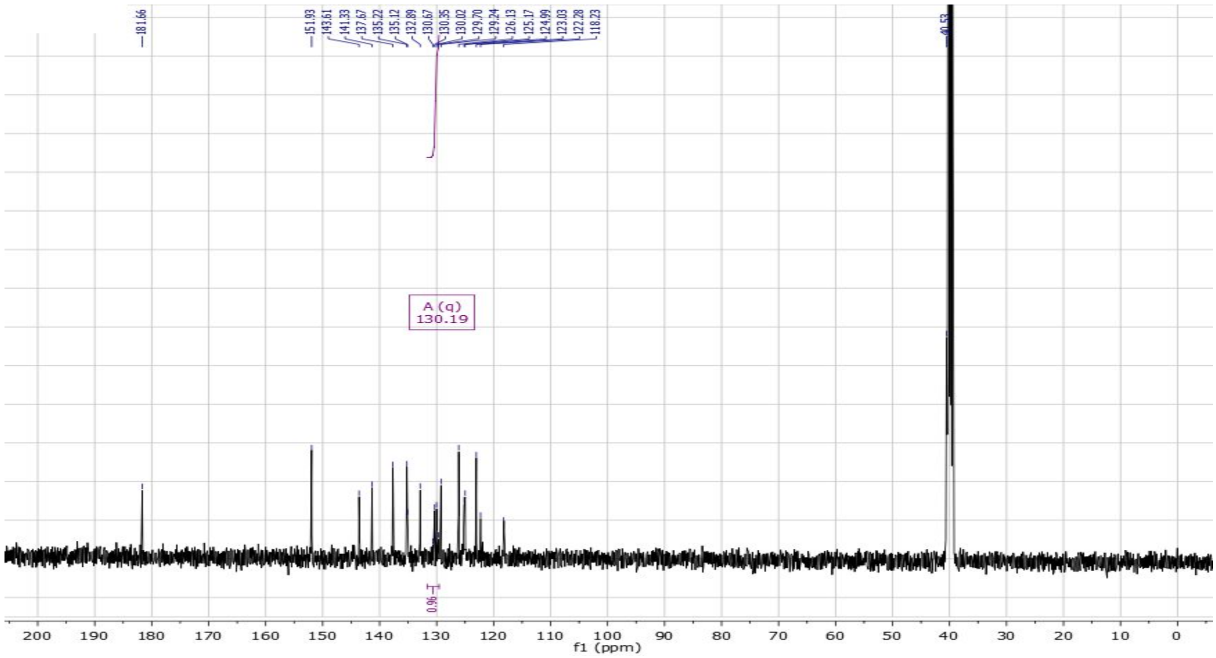

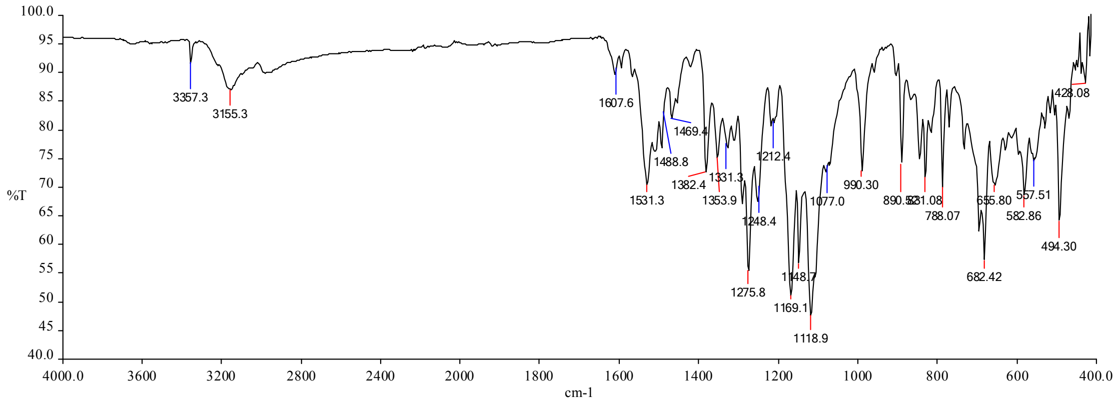

^1H NMR, ^{13}C NMR and IR spectra of **QST10**

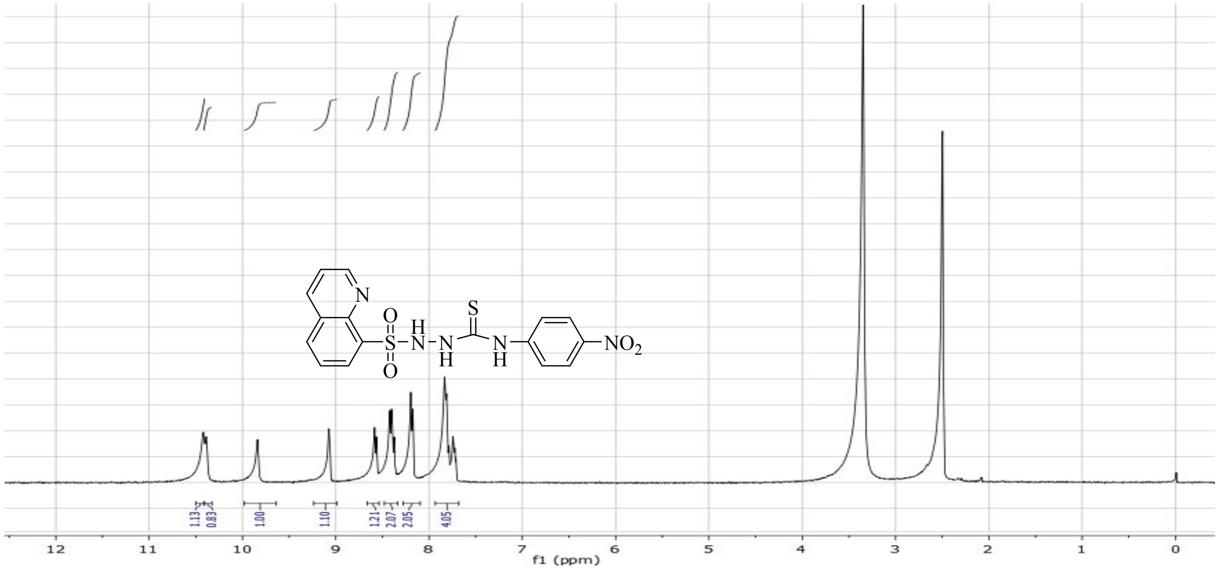

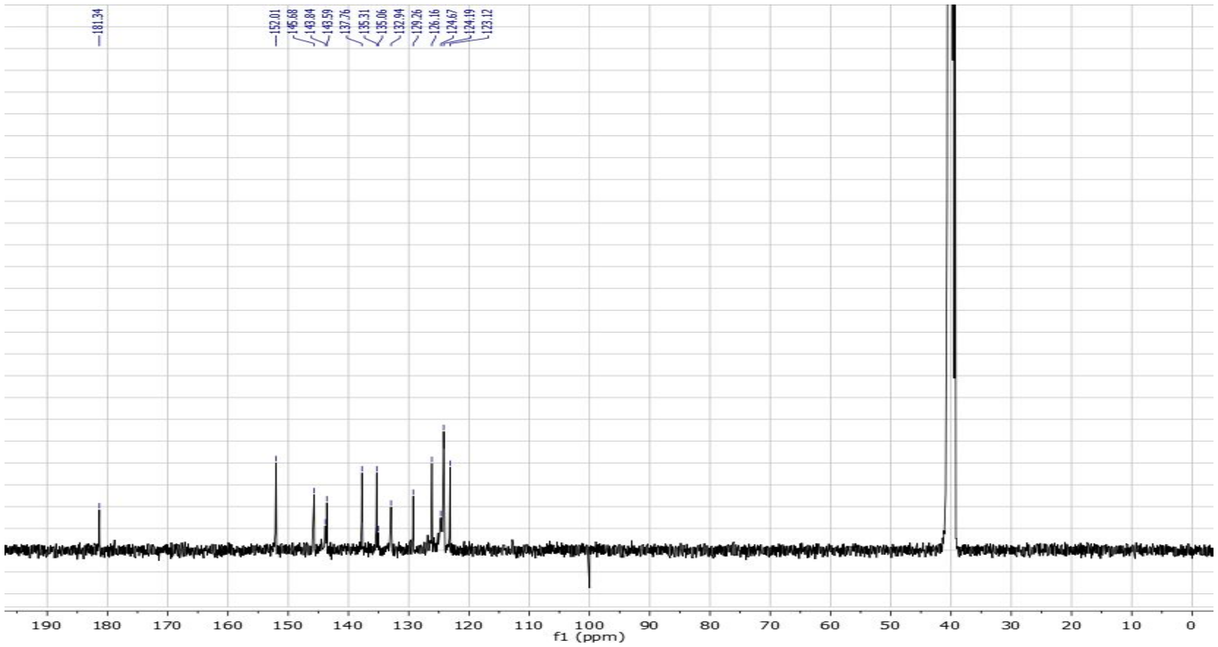

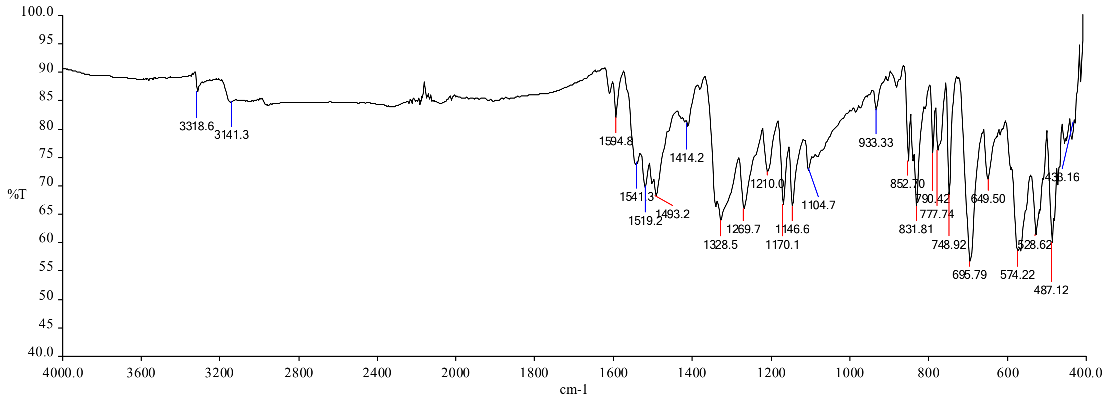

^{1}H NMR, ^{13}C NMR and IR spectra of **QST11**

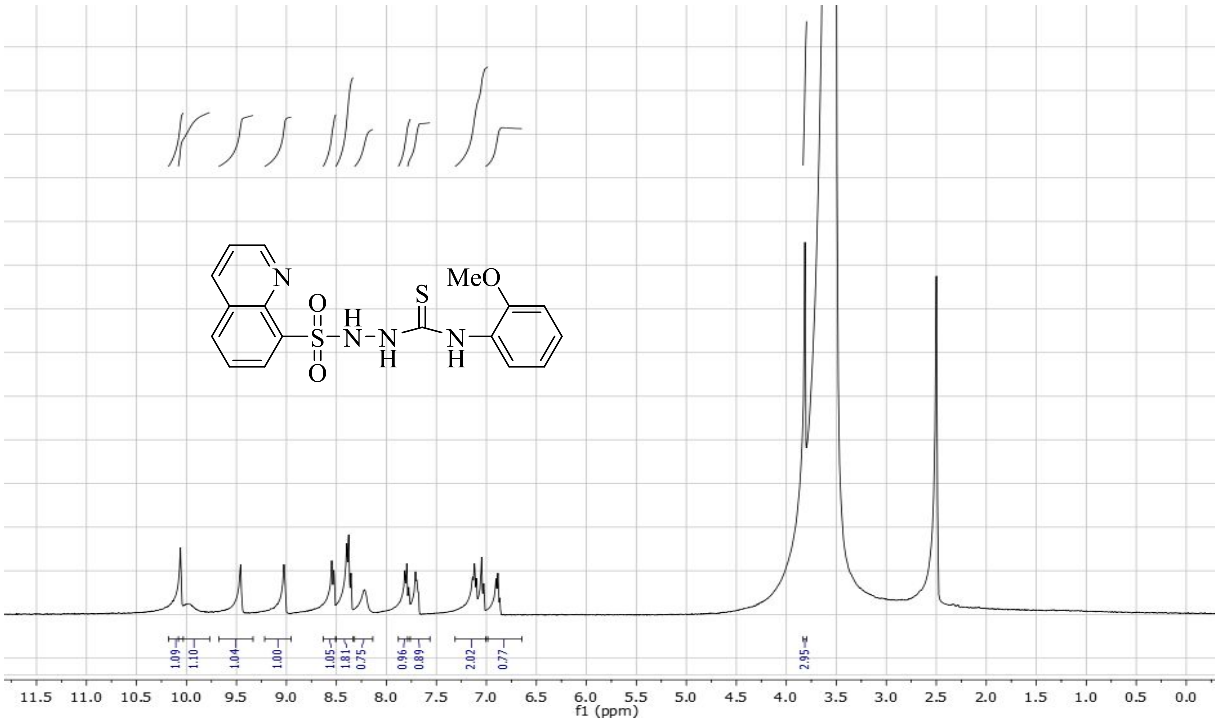

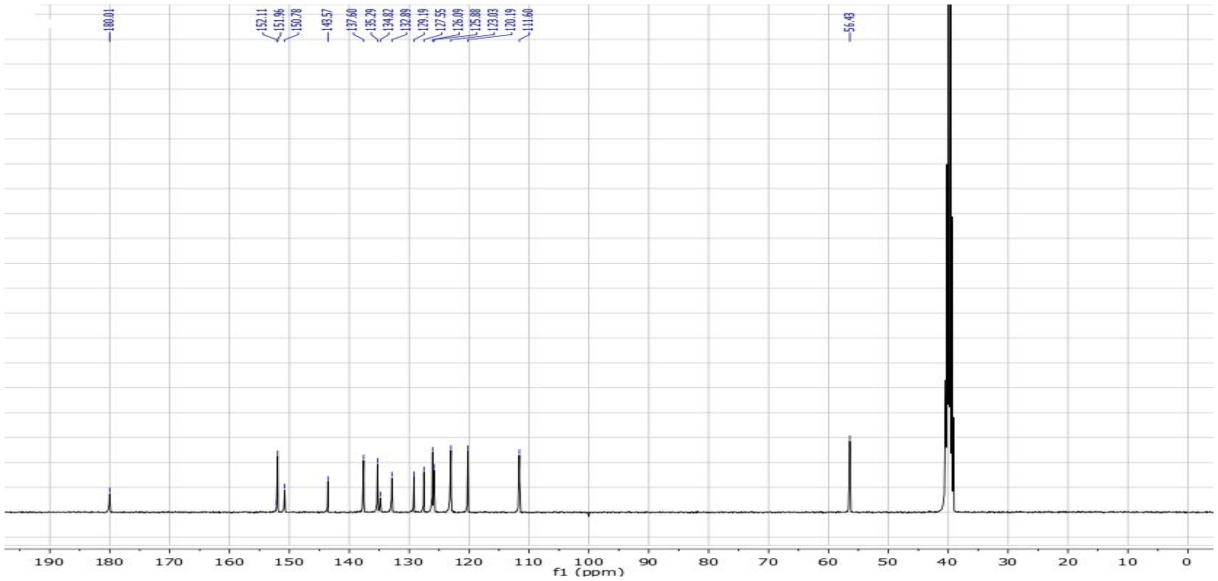

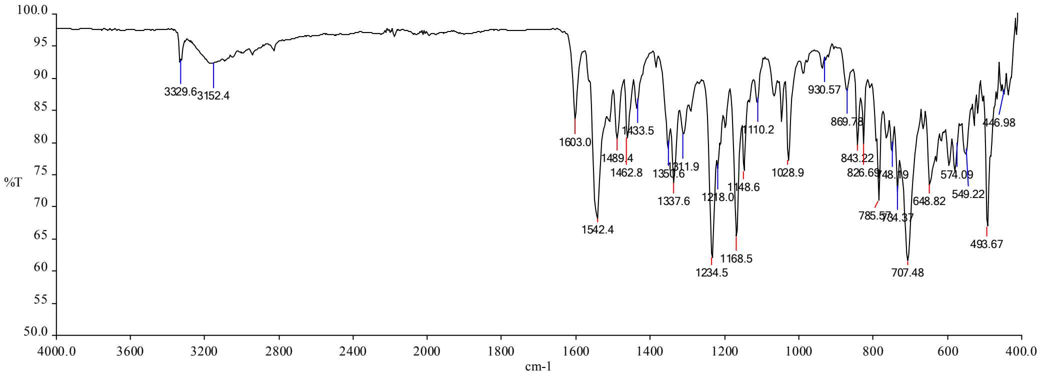

^{1}H NMR, ^{13}C NMR and IR spectra of **QST12**

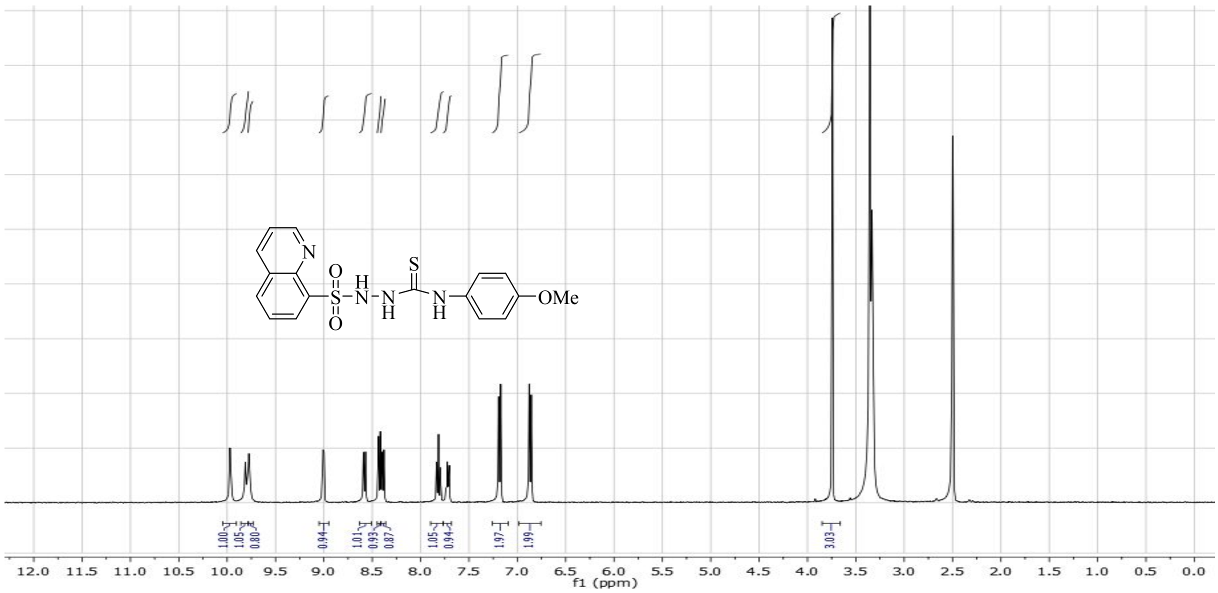

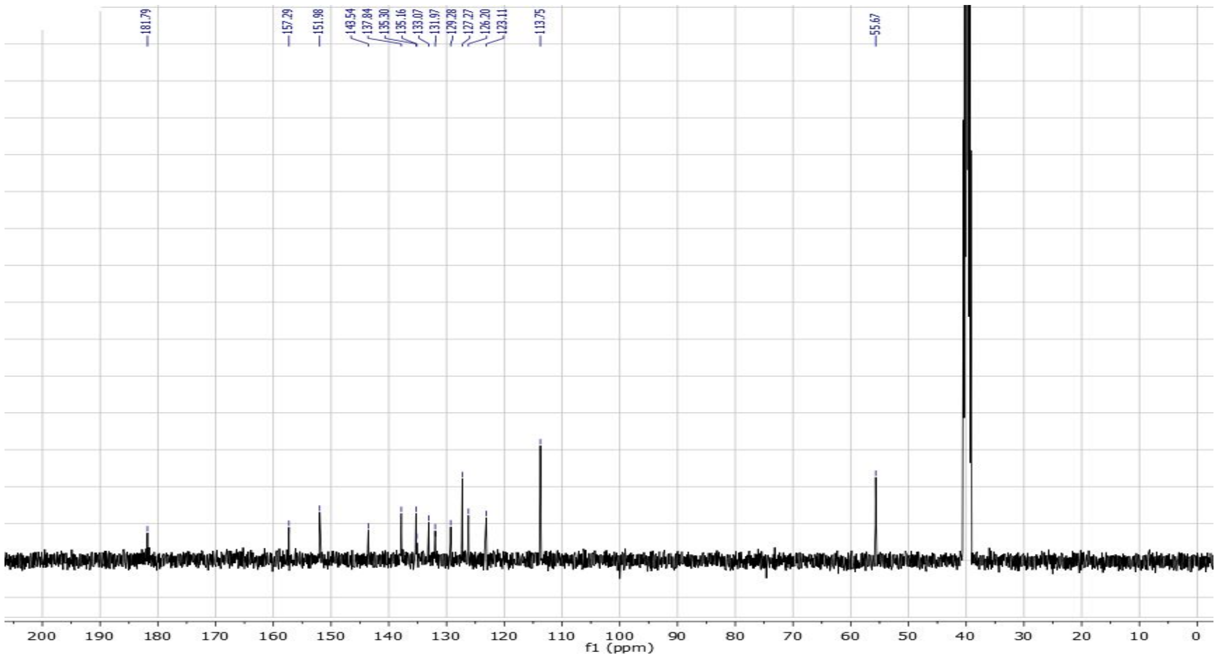

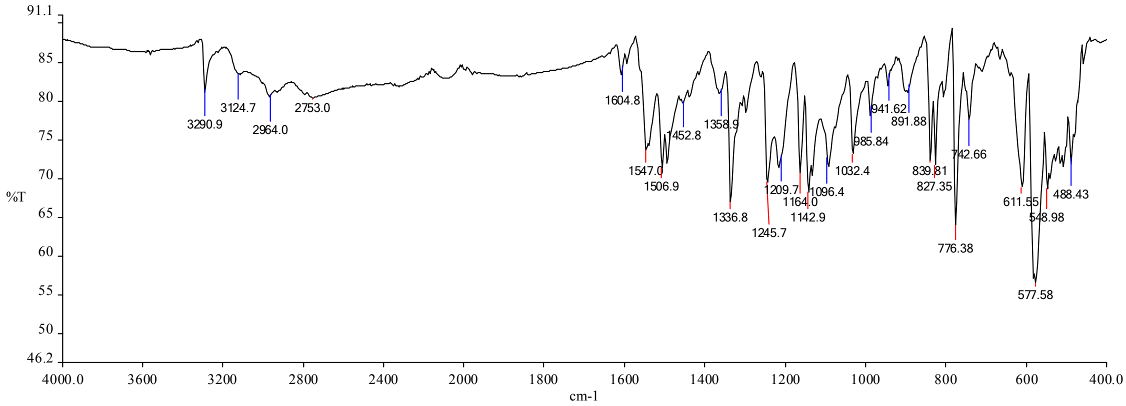

^{1}H NMR, ^{13}C NMR and IR spectra of **QST13**

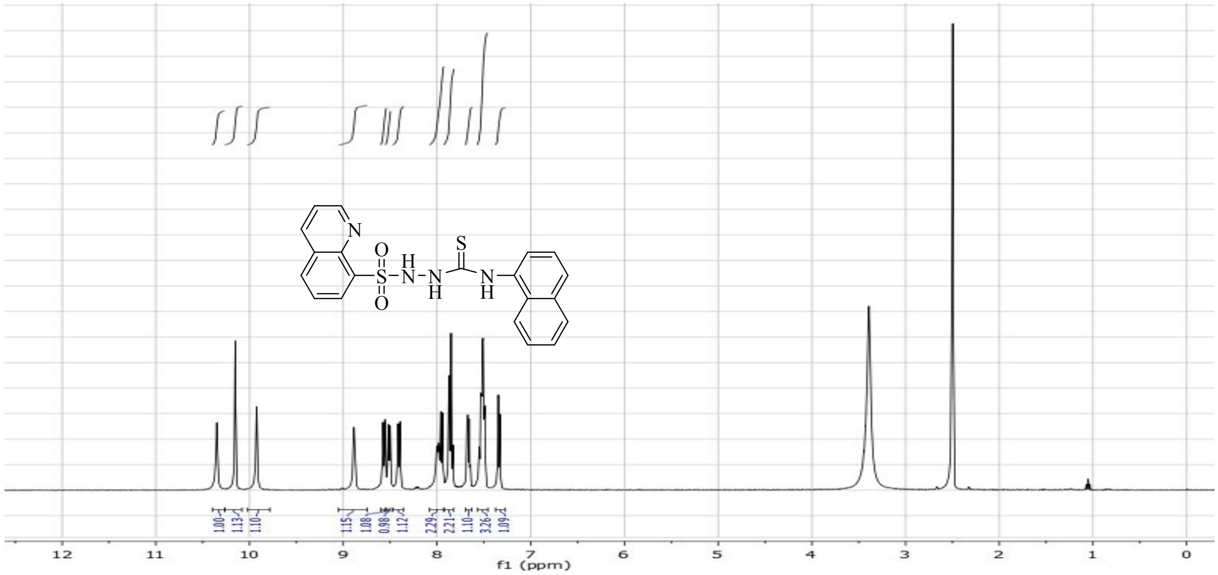

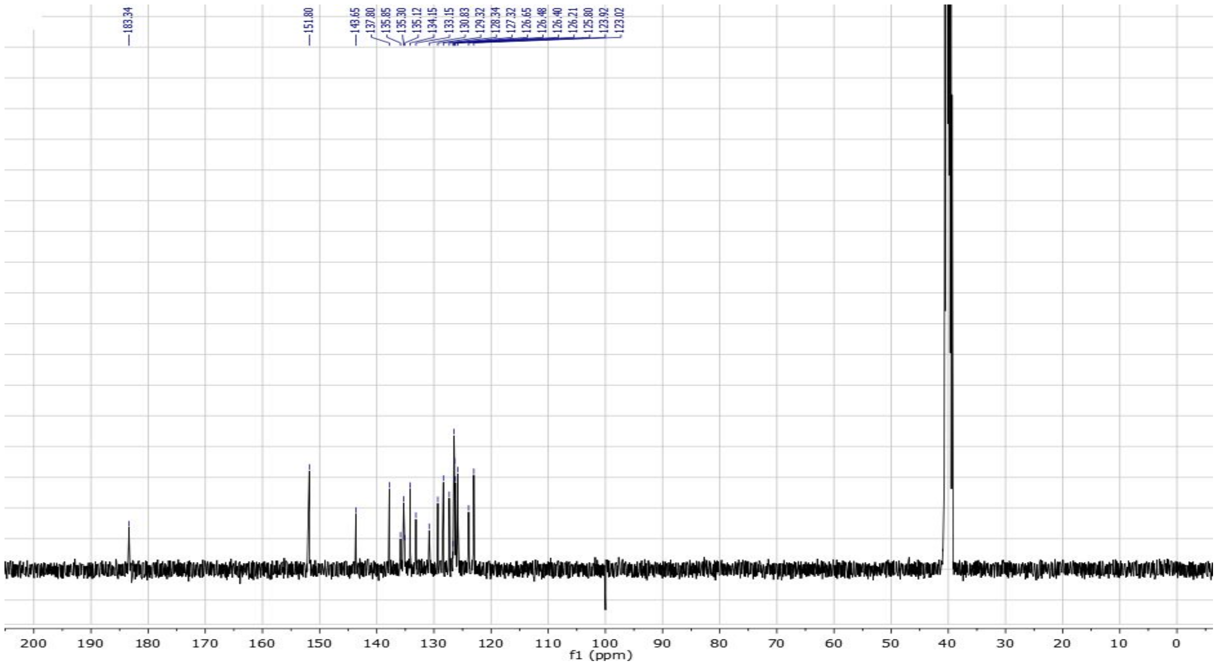

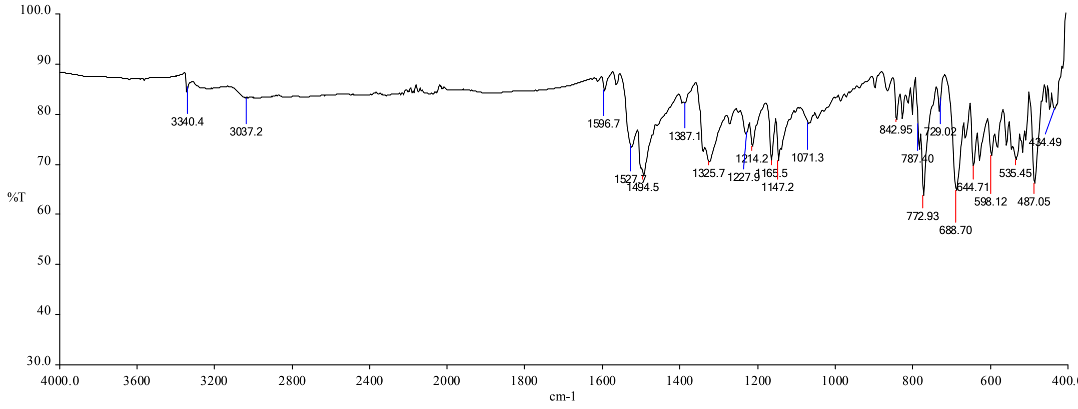

^{1}H NMR, ^{13}C NMR and IR spectra of **QST14**

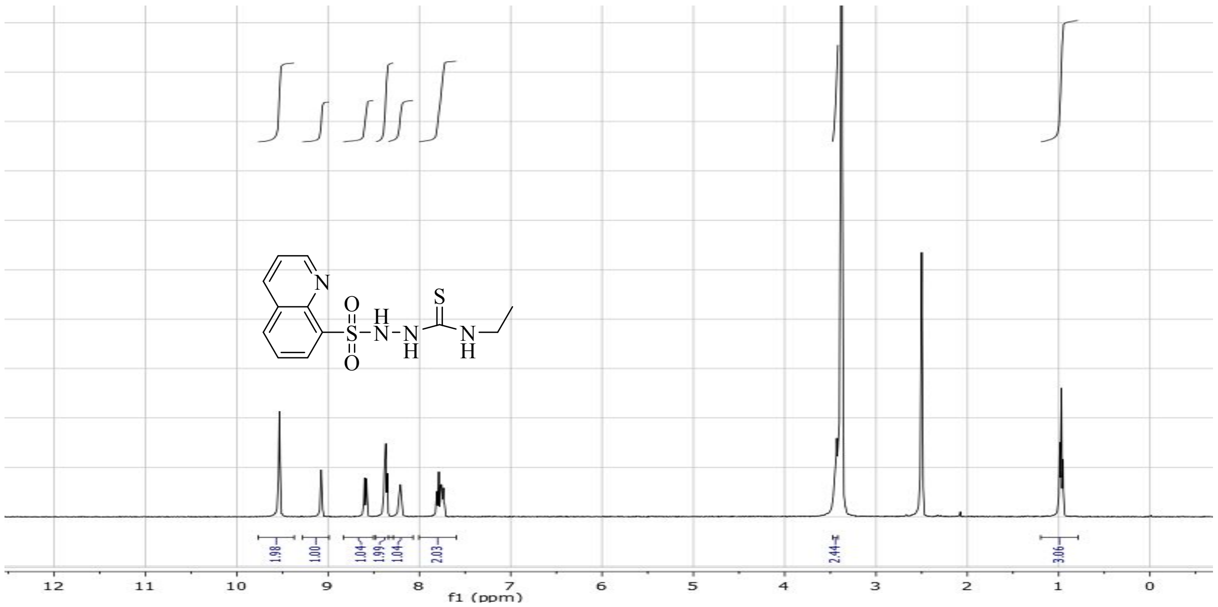

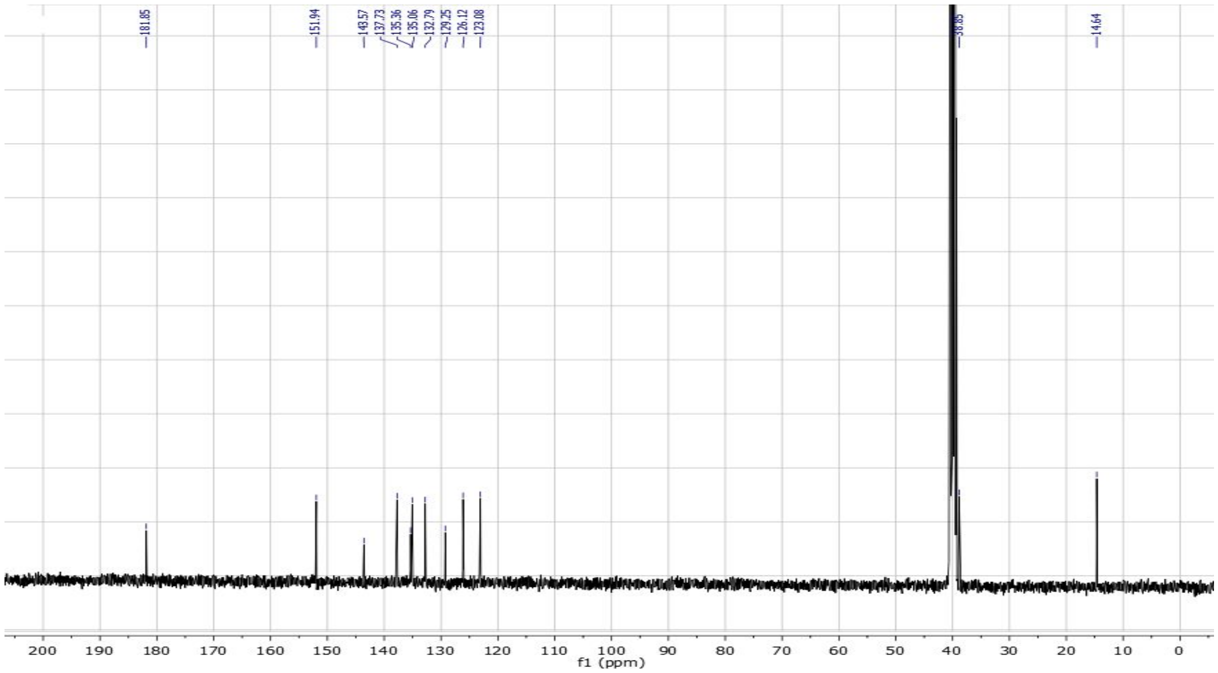

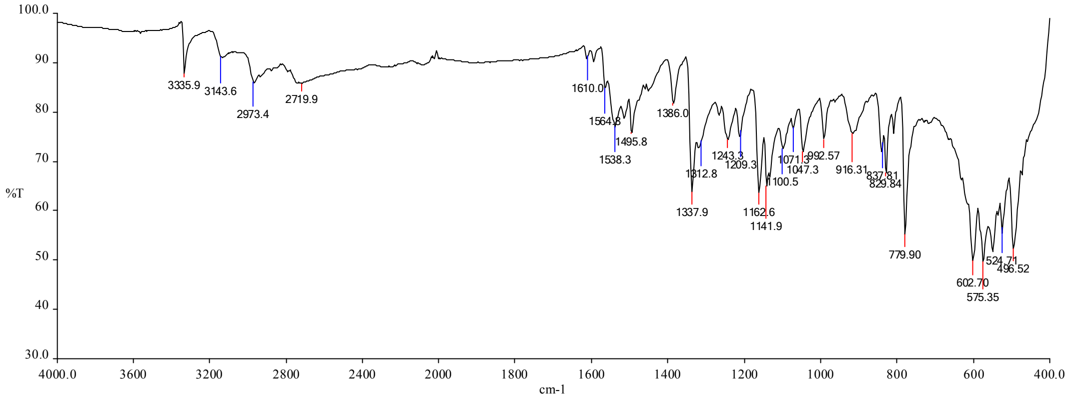

^{15}N NMR spectrum of QST3

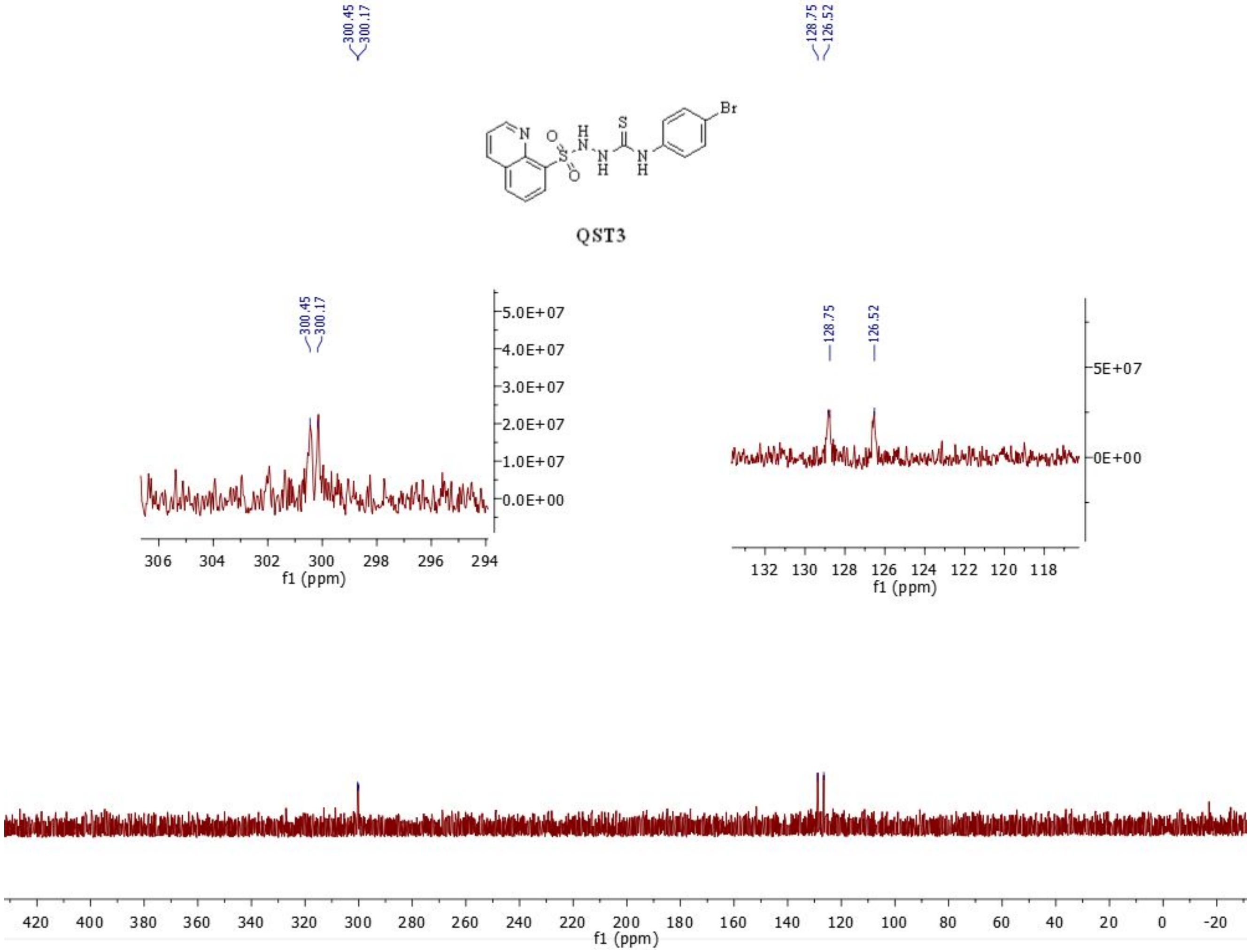

Table S7. Antimicrobial activity (MIC values, μg/mL) of QST compounds on bacterial and *Candida* species.

	S. aureus NCTC 6571	*E. faecium* ATCC 6057	*P. aeruginosa* NCTC 10332	*K. pneumonie* ATCC BAA 2146	*C. albicans* ATCC 10231
**QST 1**	>500	>500	>500	>500	>500
**QST 2**	>250	>500	>500	>500	**250**
**QST 3**	>500	>500	>500	>500	>500
**QST 4**	>500	>500	>500	>500	>500
**QST 5**	>500	>500	>500	>500	>500
**QST 6**	>250	>250	>250	>250	>250
**QST 7**	>500	>500	>500	>500	>500
**QST 8**	**250**	>500	>500	>500	>500
**QST 9**	**250**	>500	>500	>500	>500
**QST 10**	500	>500	>500	>500	**31.25**
**QST 11**	>500	>500	>500	>500	>500
**QST 12**	>500	>500	>500	>500	>500
**QST 13**	>500	>500	>500	>500	>500
**QST 14**	>500	>500	>500	>500	>500
**Vancomycin**	2	<0.25	16	200	/
**Nystatin**	/	/	/	/	0.5
